# Chaotic-Like Transfers of Energy in Hamiltonian PDEs

**DOI:** 10.1007/s00220-021-03956-9

**Published:** 2021-02-08

**Authors:** Filippo Giuliani, Marcel Guardia, Pau Martin, Stefano Pasquali

**Affiliations:** 1 Departament de Matemàtiques, Universitat Politècnica de Catalunya, Barcelona, Spain; 2 BGSMATH Barcelona Graduate School of Mathematics, Barcelona, Spain; 3 Centre de Recerca Matemàtiques, Barcelona, Spain

## Abstract

We consider the nonlinear cubic Wave, the Hartree and the nonlinear cubic Beam equations on ${\mathbb {T}}^2$ and we prove the existence of different types of solutions which exchange energy between Fourier modes in certain time scales. This exchange can be considered “chaotic-like” since either the choice of activated modes or the time spent in each transfer can be chosen randomly. The key point of the construction of those orbits is the existence of heteroclinic connections between invariant objects and the construction of symbolic dynamics (a Smale horseshoe) for the Birkhoff Normal Form truncation of those equations.

## Introduction

A fundamental question in nonlinear Hamiltonian Partial Differential Equations (PDEs) on compact manifolds is to understand how solutions can exchange energy among Fourier modes as time evolves. A way to capture such behaviors is to analyze the invariant objects of the equation (or a “good approximation of it”), such as periodic orbits or invariant tori, and to understand how they structure the global dynamics through their stable and unstable manifolds and their possible intersections. This “dynamical systems” approach works very well, for instance, for PDEs on the torus ${\mathbb {T}}^n$. Such equations can be seen as infinite dimensional systems of ODEs for the Fourier coefficients and classical perturbative arguments can be adapted to the infinite dimensional context for the analysis of stability and instability phenomena. This approach has been classically applied to the analysis of stable motions, that is KAM Theory (the literature is huge, we refer to [4] for an overview on the subject and to the reference therein). However, its application to exchange of energy phenomena is much more recent.

In the last decade there has been a lot of activity in building exchange of energy behaviors in different Hamiltonian PDEs almost exclusively for the nonlinear Schrödinger equation. They can be classified into two groups. The first one are the so-called beating solutions [19–21, 28, 29, 42]. Those are orbits that are essentially supported on a finite numbers of modes and whose energy oscillates between those modes in a certain time range.

The other group are those addressing the problem of Sobolev norm explosion. That is, constructing orbits whose energy is transferred to increasingly higher modes as time evolves [6, 8, 9, 16, 17, 22–27, 31, 32, 35, 38, 39]. Those are solutions whose dynamics is essentially supported in a large number of modes and it is related to *weak turbulence*. J. Bourgain considered this problem one of the key questions in Hamiltonian PDEs for the XXI century [7].

Most of these results rely on analyzing certain truncations of the Hamiltonian PDEs (its first order Birkhoff normal form truncation) and building invariant objects for such models. Note that these first order Birkhoff normal forms are typically non-integrable Hamiltonian systems (at least in dimension greater or equal than 2) with very complicated dynamics. Nevertheless, restricted to suitably chosen invariant subspaces those models are integrable (they have “enough” first integrals in involution), and therefore one can have a very precise knowledge of their orbits in such invariant subspaces. Most of the results cited above strongly rely on this integrability on subspaces to construct unstable motions and exchange of energy solutions. This is somewhat surprising from the point of view of (finite dimensional) dynamical systems where usually unstable motions and drifting orbits must rely on non-integrability and transverse homoclinic orbits.

Can one take advantage of the non-integrability and chaoticity of a normal form truncation to construct new types of beating solutions? Can one exploit this chaoticity/non-integrability to build new type of dynamics in Hamiltonian PDEs? This is the goal of this paper. We consider three different PDEs, a nonlinear Wave equation, a nonlinear Beam equation and the Hartree equation (see (1.1), (1.2) and (1.10) below) and we are able to show the non-integrability and chaoticity (symbolic dynamics) of its Birkhoff normal form. This allows us to obtain different types of exchange of energy behaviors for the actual PDEs in some time scales. In particular,

- Solutions which exchange energy in a chaotic-like way between a given set of modes. By chaotic-like we refer to orbits which oscillate between being supported in two different sets of modes and the “oscillation times” can be chosen “randomly”, see Theorem 1.3 below for the precise statement.
- Chaotic-like transfer of energy phenomenon: those orbits are essentially supported in a finite number of modes and the support is changing as follows. At each transition two modes get deactivated (their modulus becomes essentially constant) and we can choose randomly which new two modes are activated (their modulus starts oscillating) among certain set. See Theorem 1.4 below for the precise statement.

These results provide different types of beating solutions which are significantly different from the previous results [19, 20, 29]. The beating solutions in these papers exchange energy periodically in time and they rely on integrability and existence of action-angle variables. On the contrary, in the present paper the oscillations can be “randomly” chosen: in the first one with respect to the time and in the second one with respect to the choice of activated modes.

Our second result leads to transfer of energy. However, the transfer does not involve arbitrarily high modes and therefore does not lead to explosion of Sobolev norms. The methods in [8] for the construction of solutions exhibiting growth of the norms seem to fit very well for the NLS model [22–27]. Nevertheless, it is not clear how to apply it to other PDEs. We think that the present work could represent a first step to strengthen the strategy in [8] so that is applicable to other PDEs by incorporating tools and mechanisms inspired by the theory of Arnold diffusion. In Sect. 1.2 we relate our results to the approach developed in [8].

The key point to obtain the results in this paper is to consider certain first order truncations of the PDEs which can be treated as nearly integrable Hamiltonian systems. Then, one can apply classical methods in dynamical systems such as Melnikov Theory, shadowing arguments (Lambda lemma), hyperbolic invariant sets and symbolic dynamics.

### Main results

Consider the completely resonant cubic nonlinear Wave equation on the 2-dimensional torus

1.1 $$\begin{aligned} u_{tt}-\Delta u+u^3=0 \qquad u=u(t, x), \quad t\in {\mathbb {R}}, \quad x\in {\mathbb {T}}^2 \end{aligned}$$

and the cubic nonlinear Beam equation

1.2 $$\begin{aligned} u_{tt}+\Delta ^2 u+u^3=0\qquad u=u(t, x), \quad t\in {\mathbb {R}}, \quad x\in {\mathbb {T}}^2. \end{aligned}$$

We prove the existence of special beating solutions for such PDEs, namely solutions that exhibit transfer of energy between Fourier modes. Such solutions *u*(*t*, *x*) are mainly Fourier supported on a finite set of 4-tuples of resonant modes

1.3 $$\begin{aligned} \Lambda :=\{n^{(r)}_j\}^{r=1, \dots , N}_{j=1, \dots , 4}\subset {\mathbb {Z}}^2, \end{aligned}$$

with $N\ge 2$, in the sense that

$$\begin{aligned} u(t, x)=\sum _{j\in \Lambda } a_j(t)\,e^{\mathrm {i} j\cdot x}+R(t, x) \end{aligned}$$

where *R*(*t*, *x*) is small in some Sobolev norm. The transfers of energy between modes in $\Lambda$ are *chaotic-like*, in the following sense. Either

- one can prescribe a finite sequence of times $t_1, \dots , t_n$ and find a solution that exists for *long but finite time* exhibiting transfers of energy among the modes in $\Lambda$ at the prescribed times $t_1, \dots , t_n$

or

(b) one can prescribe a sequence of resonant tuples $\{n^{(r_n)}_{j}\}_{j=1, \dots , 4}^{n=1, \dots , k}\subseteq \Lambda$ and find a solution and a sequence of times $t_1, \dots , t_k$ such that at time zero many modes are “switched off” (modulus of the modes almost constant) and at times $t_n$ the modes $(n^{(r_n)}_{1},n^{(r_n)}_{2},n^{(r_n)}_{3},n^{(r_n)}_{4})$ are “switched on”, in the sense that they start to exchange between them.

Those phenomena are consequence of the presence of (partially) hyperbolic, finite dimensional manifolds which are approximately invariant for the Eqs. (1.1), (1.2) and possess stable and unstable invariant manifolds that intersect transversally within some energy level.

We look for beating solutions in the subspace of functions Fourier supported on

1.4 $$\begin{aligned} {\mathbb {Z}}^2_{\mathrm {odd}}:=\left\{ (j^{(1)}, j^{(2)})\in {\mathbb {Z}}^2 \,: \,\, j^{(1)}\,\,\text {odd}\,\,,\,\,j^{(2)}\,\,\text{ even }\right\} , \end{aligned}$$

which is invariant under the flow of the Eqs. (1.1), (1.2) (see [40]). We restrict the equations to this subspace so that the origin becomes an elliptic fixed point and the variational equation are

1.5 $$\begin{aligned} \ddot{u}_j+\lambda _j^2 u_j=0 \quad j\in {\mathbb {Z}}^2_\mathrm {odd}\end{aligned}$$

where $\lambda _j=|j|$ [for the Wave Eq. (1.1)] and $\lambda _j=|j|^2$ [for the Beam Eq. (1.2)]. Note that, if one does not restrict to ${\mathbb {Z}}^2_{\mathrm {odd}}$, one has to deal with the harmonic 0 which is not elliptic.

Restricted to ${\mathbb {Z}}^2_{\mathrm {odd}}$, the variational equations (1.5) are superposition of decoupled harmonic oscillators, hence all solutions are periodic/quasi-periodic/almost-periodic in time and there is no transfer of energy between the linear modes when time evolves. This implies that the existence of beating solutions (if any) depend on the presence of the nonlinearities. To catch the nonlinear effects in a neighborhood of an elliptic equilibrium we perform a Birkhoff normal form analysis. Namely we construct changes of coordinates^1^ that transform the Hamiltonian of the Eqs. (1.1), (1.2) into a Hamiltonian of the form

1.6 $$\begin{aligned} K=K^{(2)}+K^{(4)}+{\mathcal {R}}, \end{aligned}$$

where $K^{(i)}$ are homogenous terms of degree *i* and ${\mathcal {R}}$ is a function that can be considered as a small perturbation. Then, one can consider the truncated system

1.7 $$\begin{aligned} {{\mathcal {N}}}:=K^{(2)}+K^{(4)}, \end{aligned}$$

called *normal form* [see (3.7) below for the explicit formulas], as a model which describes the effective dynamics of Eqs. (1.1), (1.2) for a certain range of times.

The normal form Hamiltonian ${{\mathcal {N}}}$ possesses many finite-dimensional, symplectic, invariant subspaces of the form $V_\Lambda :=\{ u_j=0\,\, \,\,\forall j\notin \Lambda \}$, where $\Lambda \subset {\mathbb {Z}}^2_\mathrm {odd}$ is a finite set. We shall prove the following.

#### Theorem 1.1

Let $N\ge 2$. There exist sets^2^$\Lambda \subset {\mathbb {Z}}^2_\mathrm {odd}$ of cardinality 4*N* such that $V_\Lambda$ is invariant by the dynamics of ${{\mathcal {N}}}$ and the following holds.

(i) Let $N=2$. Then, the flow $\Phi _t$ associated to ${{\mathcal {N}}}$ in $V_\Lambda$ has the following property. There exists a section $\Pi$ transverse to the flow $\Phi _t$ such that the induced Poincaré map
  $$\begin{aligned} {{\mathcal {P}}}:{\mathcal {U}}=\mathring{{\mathcal {U}}}\subset \Pi \rightarrow \Pi \end{aligned}$$
  has an invariant set $X\subset {\mathcal {U}}$ which is homeomorphic to $\Sigma \times {\mathbb {T}}^5$ where $\Sigma ={{\mathbb {N}}}^{\mathbb {Z}}$ is the set of sequences of natural numbers. Moreover, the dynamics of ${{\mathcal {P}}}:X\rightarrow X$ is topologically conjugated to the following dynamics
  $$\begin{aligned} {{\widetilde{{{\mathcal {P}}}}}}:\Sigma \times {\mathbb {T}}^5\rightarrow \Sigma \times {\mathbb {T}}^5, \qquad {{\widetilde{{{\mathcal {P}}}}}}(\omega , \theta )=(\sigma \omega , \theta +f(\omega )) \end{aligned}$$
  where $\sigma$ is the usual shift $(\sigma \omega )_k=\omega _{k+1}$ and $f:\Sigma \rightarrow {\mathbb {R}}^5$ is a continuous function.
  Namely ${{\mathcal {P}}}$ has a Smale horseshoe of infinite symbols as a factor.
(ii) There exist *N* partially hyperbolic $2(N+1)$-dimensional tori ${\mathbb {T}}_1, \dots , {\mathbb {T}}_N$ invariant for the restriction of the normal form Hamiltonian ${\mathcal {N}}$ at the subspace $V_\Lambda$ which have the following property. Take arbitrarily small neighborhoods $V_i$ of ${\mathbb {T}}_i$ and any sequence $\{p_i\}_{i\ge 1}\subset {{\mathbb {N}}}^{{\mathbb {N}}}$. Then, there exists an orbit *u*(*t*) and a sequence of times $\{t_i\}_{i\ge 1}$ such that
  $$\begin{aligned} u(t_i)\in V_{p_i}. \end{aligned}$$

#### Remark 1.2

The set $\Lambda \subset {\mathbb {Z}}^2$ is the union of *N* resonant tuples (with certain properties). The “shape” of the resonant tuples in ${\mathbb {Z}}^2$ are different for the Beam and Wave Equations. For the Beam equation, as for the cubic nonlinear Schrödinger equation, are rectangles with vertices in ${\mathbb {Z}}^2$. For the Wave equation are modes $n_1,n_2,n_3,n_4\in {\mathbb {Z}}^2$, which satisfy

$$\begin{aligned} n_1-n_2+n_3-n_4=0,\quad | n_1|-|n_2|+|n_3|-|n_4|=0. \end{aligned}$$

Those tuples form a parallelogram inscribed on an ellipse with foci at $F_1=0$ and $F_2=n_1+n_2$ and semi-major axis $a=(|n_1|+|n_2|)/2$.

Let us explain in which sense there are *many sets* $\Lambda \subset {\mathbb {Z}}^2$ for which Theorem 1.1 (and also Theorems 1.3 and 1.4 below) are satisfied. Theorem 1.1 relies on proving the transverse intersection of certain invariant manifolds. This transversality is proven by perturbative methods and, therefore, we need ${\mathcal {N}}|_\Lambda$ to be close to integrable. For the Wave (1.1) and Beam (1.2) equations this relies on choosing appropriate sets $\Lambda$. The precise statement goes as follows. Fix $\varepsilon >0$ (which will measure the closeness to integrability). Then, for any $R\gg 1$, one can choose the resonant tuples in the set $\Lambda$ generically in the annulus

$$\begin{aligned} R(1-\varepsilon )\le |n|\le R (1+\varepsilon ). \end{aligned}$$

Generically means that one has to exclude the zero set of a finite number of algebraic varieties (and the number of those is independent of $\varepsilon$ and *R*).

**Fig. 1:**
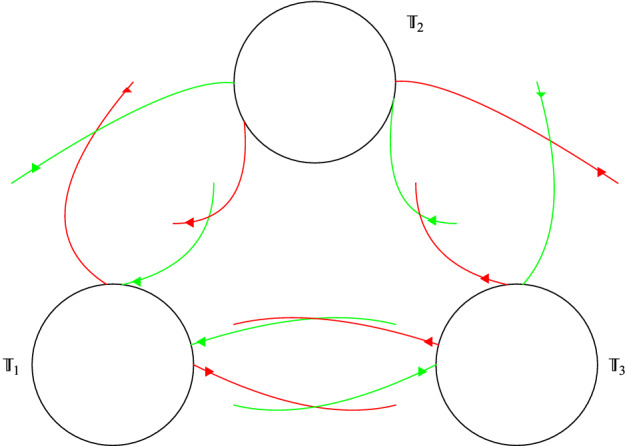
Invariant tori with their stable (green) and unstable (red) invariant manifolds. This transition chain of tori (plus the Lambda lemma) gives the orbits of Item (ii) of Theorem 1.1 which visit the invariant tori with any prescribed orbit

The items (a) and (b) above are consequences respectively of items (i) and (ii) in Theorem 1.1. Let us make some remark on the type of dynamics for the normal form Hamiltonian ${\mathcal {N}}$.

- Item (i) of Theorem 1.1 gives the existence of invariant sets for the Birkhoff normal form truncation which possess chaotic dynamics. Such chaotic dynamics is obtained through the classical Smale horseshoe dynamics for a suitable Poincaré map. This invariant set is constructed in the neighborhood of homoclinic points to an invariant tori orbit (which becomes a periodic orbit for a suitable symplectic reduction). The (infinite) symbols codify the closeness to the invariant manifolds of the periodic orbit, and therefore the larger the symbol is the longer the return time to the section $\Pi$ is. In particular, one can construct orbits which take longer and longer time to return $\Pi$ for higher iterates. Even if the theorem, as stated, gives the existence of one invariant set, one actually can construct a Smale horseshoe at each energy level.
- Item (ii) of Theorem 1.1 gives orbits which visit (possibly infinitely many times) a given set of invariant tori in any prescribed order. The construction of such orbits follows the classical strategy of Arnold Diffusion [1]. That is, is a consequence of the existence of a chain of invariant tori (again periodic orbits in a suitable symplectic reduction) connected by transverse heteroclinic connections (see Fig. 1) plus a classical shadowing argument (Lambda lemma, see for instance [14]).
  This is radically different from the approach in [8, 24]. In these papers, the authors consider the normal form associated to the nonlinear cubic Schrödinger equation. This normal form has “extra integrability”, due to the symmetries of the model, and the considered heteroclinic orbits are not transverse. Therefore, the associated shadowing arguments are more delicate. We refer to [10] for a thorough analysis of non-transverse shadowing arguments. In particular, the authors of this paper show that the number of dimensions needed for the shadowing depend on the number of tori the orbits have to visit (what they called the *dropping the dimension* mechanism).
  As for item (i) one can obtain the explained behavior at each energy level. Indeed, the invariant tori come in families parameterized by the energy level and therefore one can obtain this shadowing behavior at each energy level as well.

Note that the knowledge of the orbits obtained in Theorem 1.1 is *for all time*. If one adds the errors dropped from the original equation, that is ${\mathcal {R}}$ in (1.6), one can obtain orbits for equations (1.1), (1.2) which follow the orbits of Theorem 1.1 for some time scales. Next theorem gives solutions of Eqs. (1.1) and (1.2) which (approximately) behave as those obtained in Item (i) of Theorem 1.1.

#### Theorem 1.3

Fix $0<\varepsilon \ll 1$. Then for a large choice of sets $\Lambda =\{n_i\}_{i=1}^8\subset {\mathbb {Z}}^2$ as in (1.3) there exists $\mathtt {T}_0\gg 1$ such that for all $\mathtt {T}\ge \mathtt {T}_0$ there exists $M_0>0$ such that for all $M\ge M_0$ there exists $\delta _0=\delta _0(M, \varepsilon , \mathtt {T})>0$ such that $\forall \delta \in (0, \delta _0)$ the following holds.

Choose any $k\ge 1$ and any sequence $\{ m_j\}_{j=1}^k$ such that $m_j\ge M_0$ and^3^$\sum _{j=1}^k m_j\le M-k$. Then, there exists a solution *u*(*t*, *x*) of (1.1), (1.2) for $t\in [0, \delta ^{-2} M \mathtt {T}]$ of the form

$$\begin{aligned} u(t, x)=\frac{\delta }{\sqrt{2}}\sum _{i=1}^ 8 |n_i|^{-\kappa /2}\left( a_{n_i}( t)\,e^{\mathrm {i} n_i \cdot x}+\overline{a_{n_i}}(t)\,e^{-\mathrm {i} n_i \cdot x}\right) +R_1(t, x) \end{aligned}$$

where $\kappa =1$ for the Wave equation (1.1) and $\kappa =2$ for the Beam equation (1.2), and $\sup _{t\in [0, \delta ^{-2} M \mathtt {T}]}\Vert R_1 \Vert _{H^s({\mathbb {T}}^2)}\lesssim _s \delta ^{3/2}$ for all $s\ge 0$. The first order $\{ a_{n_i}\}_{i=1\dots 8}$ satisfies

$$\begin{aligned} \begin{aligned} |a_{n_1}(t) |^2&=|a_{n_3} (t) |^2=1- |a_{n_2}(t) |^2=1-|a_{n_4} (t) |^2,\\ |a_{n_5}(t) |^2&=|a_{n_7} (t) |^2=1- |a_{n_6}(t) |^2=1-|a_{n_8} (t) |^2, \end{aligned} \end{aligned}$$

and has the following behavior.

- **First resonant tuple (Periodic transfer of energy):** There exists a $\mathtt {T}$-periodic function *Q*(*t*), independent of $\delta$ and satisfying $\min _{[0,\mathtt {T}]} Q(t) < \varepsilon$ and $\max _{[0,\mathtt {T}]} |Q(t)| > 1-\varepsilon$, such that
  $$\begin{aligned} |a_{n_1}(t) |^2= Q (\delta ^{2} t)+R_2(t)\qquad \text {with} \qquad \sup _{t\in {\mathbb {R}}} |R_2(t) |\le \varepsilon .\end{aligned}$$
- **Second resonant tuple (Chaotic-like transfer of energy):** There exists a sequence of times $\{t_j\}_{j=0}^{k}$ satisfying $t_0=0$ and
  $$\begin{aligned} t_{j+1}=t_j+\delta ^{-2}\mathtt {T}\left( m_j+\theta _j\right) \qquad \text {with}\qquad \theta _j\in (0,1) \end{aligned}$$
  such that
  $$\begin{aligned} |a_{n_5}( t_j) |^2=\frac{1}{2}. \end{aligned}$$
  Moreover, there exists another sequence $\{{\bar{t}}_j\}_{j=1\ldots k}$ satisfying $t_j<{\bar{t}}_j<t_{j+1}$ such that,
  1.8 $$\begin{aligned} \begin{aligned} |a_{n_5}(t) |^2&>\frac{1}{2}\qquad \text {for}\qquad t\in (t_j,{\bar{t}}_j)\\ |a_{n_5}(t) |^2&<\frac{1}{2}\qquad \text {for}\qquad t\in ({\bar{t}}_j,t_{j+1}) \end{aligned} \end{aligned}$$
  and
  1.9 $$\begin{aligned} \sup _{t\in (t_j,{\bar{t}}_j)}|a_{n_5}(t) |^2\ge 1-\varepsilon \qquad \text { and }\qquad \inf _{t\in ({\bar{t}}_j, t_{j+1})}|a_{n_5}(t) |^2\le \varepsilon . \end{aligned}$$

Note that the first order $\{\delta a_{n_i}\}_{i=1\ldots 8}$ are the trajectories obtained in Theorem 1.1-(i) which belong to the horseshoe. This phenomenon is genuinely nonlinear since for the linear equation the *actions* $|a_{n_i}(t)|^2=\text {constant}$.

**Fig. 2:**
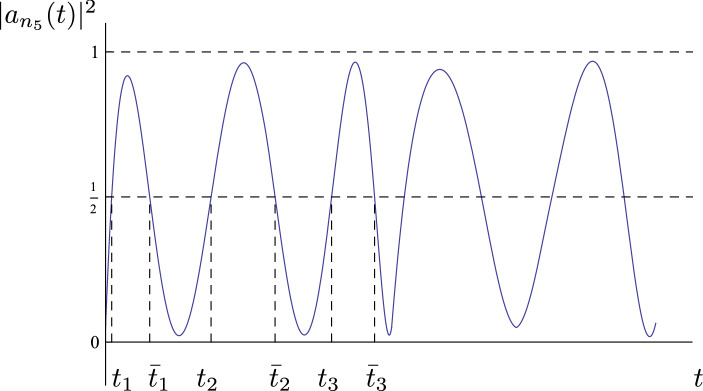
An example of the evolution of the energy $|a_{n_5}(t)|^2$ as time evolves. The energy is a multi-bump like function. It assumes the value 1/2 at the “random” times $t=t_j$ and also at $t=\bar{t}_j$. The randomness in the $t_j$’s prescribes the separation of the bumps. The larger is the increment $t_{j+1}-t_j$, the more separated are the corresponding bumps. This shows that one can obtain very complicated energy transfer behaviors for the second resonant tuple

The first resonant tuple has a periodic beating behavior similar to [20]. On the contrary, the behavior of the second resonant tuple is radically different. The modulus of the modes $a_{n_i}$, $i=5,6,7,8$ “oscillate” from being ${\mathcal {O}}(\varepsilon )$ to being ${\mathcal {O}}(\varepsilon )$-close to 1 (see Fig. 2). However, the sequence of times $\{t_j\}$ in which all the modes in the tuple have the same modulus, that is

$$\begin{aligned} |a_{n_5}( t_j) |^2=|a_{n_6}( t_j) |^2=|a_{n_7}( t_j) |^2= |a_{n_8}( t_j) |^2=\frac{1}{2}, \end{aligned}$$

(and the modulus of $$a_{n_5}$$ and $$ a_{n_7}$$ is increasing) can be chosen randomly as any (large enough) integer multiple of $$\mathtt {T}$$.

Finally, let us explain the role of the constant $\mathtt {T}$ in the theorem. To build the horseshoe in Theorem 1.1, we apply a symplectic reduction to ${\mathcal {N}}|_\Lambda$ [see (1.7)] which leads to a 2 degree of freedom Hamiltonian. For this Hamiltonian we construct a periodic orbit with transverse invariant homoclinic orbits. The time $\mathtt {T}$ is the period of this periodic orbit and can be taken arbitrarily big.

Now we state the second main result of this paper, which gives solutions of Eqs. 1.1 and 1.2 which (approximately) behave as those obtained in Item (ii) of Theorem 1.1.

#### Theorem 1.4

Let $N\ge 2$, $k\gg 1$, $0<\varepsilon \ll 1$. Then for a large choice of a set $\Lambda :=\{n^{(r)}_j\}^{r=1, \dots , N}_{j=1, \dots , 4} \subset {\mathbb {Z}}^2$ as in (1.3) there exist $\delta _0>0$, $T>0$, such that for any $\delta \in (0,\delta _0)$ and any sequence $\omega =(\omega _1, \dots , \omega _k), \omega _i\in \{1, \dots , N \}$, there exists a solution *u*(*t*, *x*) of the (1.1), (1.2) of the form

$$\begin{aligned} u(t, x)=\frac{\delta }{\sqrt{2}}\sum _{n\in \Lambda } |n|^{-\kappa /2}\left( a_{n}(t)\,e^{\mathrm {i} n \cdot x}+\overline{a_{n}}(t)\,e^{-\mathrm {i} n \cdot x}\right) +R_3(t, x) \qquad t\in [0, \delta ^{-2} T] \end{aligned}$$

where $\kappa =1$ for the Wave Eq. (1.1) and $\kappa =2$ for the Beam Eq. (1.2), $\sup _{t\in [0, \delta ^{-2} T]} \Vert R_3(t, x) \Vert _{H^s({\mathbb {T}}^2)}\lesssim _s \delta ^{3/2}$ for all $s\ge 0$, and the first order $\{ a_{n}\}_{n\in \Lambda }$, has the following behavior:

There exist some $\alpha _p,\beta _p$ satisfying

$$\begin{aligned} \alpha _p<\beta _p<\alpha _{p+1} \qquad \text { and }\qquad \beta _{p}-\alpha _p\gtrsim |\ln \varepsilon |, \quad p=1, \dots , k \end{aligned}$$

such that, if one splits the time interval as $[0, \delta ^{-2} T]=I_1\cup J_{1, 2}\cup I_2\cup J_{2, 3}\cup \dots \cup J_{k-1, k }\cup I_k$ with

$$\begin{aligned} I_p=[\delta ^{-2}\alpha _p, \delta ^{-2}\beta _p],\quad J_{p,p+1}=[\delta ^{-2}\beta _p,\delta ^{-2}\alpha _{p+1}], \end{aligned}$$

such that $\{a_{n}\}_{n\in \Lambda }$ satisfies:

- In the **beating-time** intervals $I_p$, there exists $t_p>0$ such that
  $$\begin{aligned} \sup _{t\in I_p} \Big | |a_{n^{(\omega _p)}_1}(t)|^2-Q(\delta ^2 t-t_p) \Big |&\le \varepsilon \\ \sup _{t\in I_i} |a_{n^{(r)}_1}(t)|^2&\le \varepsilon \quad&\text { for }&\qquad r=1, \dots , N, \quad r\ne \omega _p, \end{aligned}$$
  where *Q*(*t*) is the periodic function given by Theorem 1.3.
- In the **transition-time** intervals $J_{p, p+1}$,
  $$\begin{aligned} \sup _{t\in J_{p, p+1}} |a_{n^{(r)}_1}(t)|^2&\ge 1-\varepsilon \qquad \quad&\text { for }&\qquad r= 1, \dots , N \end{aligned}$$

and $|a_{n^{(r)}_1}(t)|^2=|a_{n^{(r)}_3}(t)|^2$ , $|a_{n^{(r)}_j}(t)|^2=1-|a_{n^{(r)}_{1}}(t)|^2$ with $j=2, 4$ and $r=1, \dots , N$.

**Fig. 3:**
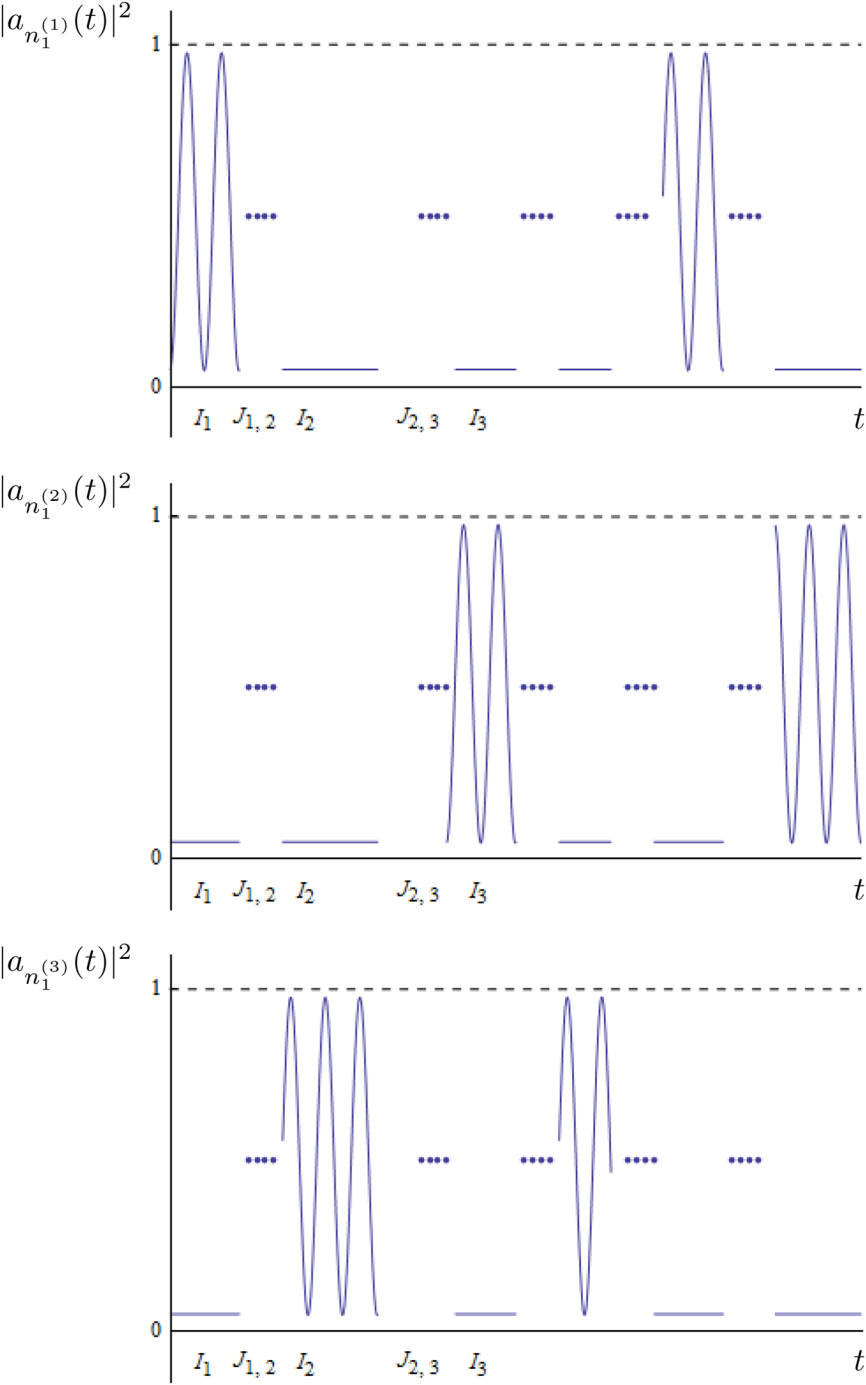
An example of the evolution of $|a_{n_1}^{(j)}(t)|^2$, $j=1, 2, 3$ of a solution obtained by Theorem 1.4 as time evolves. We consider $N=3$ and the sequence of modes which are “activated” is $\omega =\{ 1, 3, 2, 3, 1, 2\ldots \}$

The solutions obtained in this theorem are approximations of those obtained in Item (ii) of Theorem 1.1 and possess two different regimes. The orbits of Theorem 1.1 are obtained by shadowing a sequence of invariant tori (periodic orbits for a suitable symplectic reduction) connected by transverse heteroclinic orbits. Then, what we call *beating-time* intervals are the time intervals where the orbit is in a small neighborhood of each of the periodic orbits. In this regime, (the moduli of) some modes oscillate periodically, whereas the others are at rest. The *transition-time* intervals correspond to time intervals in which the orbit is “traveling” along a heteroclinic orbit and is “far” from all periodic orbits. In this regime, all modes undergo a drastic change to drift along the heteroclinic connection (see Fig. 3).

*Hartree equation.* Similar results hold true also for the Hartree equation

1.10 $$\begin{aligned} \mathrm {i} u_t=\Delta u+(V\star |u |^2)\,u\qquad u=u(t, x), \quad t\in {\mathbb {R}}, \quad x\in {\mathbb {T}}^2 \end{aligned}$$

with a convolution potential $V(x)=\sum _{j\in {\mathbb {Z}}^2} V_j\,e^{\mathrm {i} j \cdot x}$ such that

1.11 $$\begin{aligned} V:{\mathbb {T}}^2\rightarrow {\mathbb {R}}, \quad V(x)=V(-x) \end{aligned}$$

and assuming the following hypothesis. Once fixed the set $\Lambda \subset {\mathbb {Z}}^2$, the Fourier coefficients $V_j$ of the potential with $j=n_1-n_2$ for some $n_1, n_2\in \Lambda$ satisfy

1.12 $$\begin{aligned} \quad V_j=1+\varepsilon \gamma _j \quad \text {with}\quad \varepsilon \ll 1. \end{aligned}$$

Assume that the coefficients $\gamma _j$ satisfy a non-degeneracy condition which is of codimension 1 and take $\varepsilon$ small enough. Then, the Hartree equation has solutions of the form

$$\begin{aligned} u(t, x)=\delta \sum _{n\in \Lambda } a_{n}(t)\,e^{\mathrm {i} n \cdot x}+R(t, x) \end{aligned}$$

where the first order $\{a_{n}\}$ and the remainder *R* satisfy the statements given either in Theorem 1.3 (where $R\rightsquigarrow R_3$) or 1.4 (where $R\rightsquigarrow R_4$) .

Since the obtaining of such behaviors for the Hartree equation is the same as for Wave and Beam equations, in Sects. 3–7 we prove the results together for the three equations.

*Comments to Theorems* 1.3, 1.4

- **Smale Horseshoes in PDEs:** Theorem 1.1 provides a Smale Horseshoe for the Birkhoff normal form. This invariant set is partially hyperbolic and partially elliptic if considered in the whole infinite dimensional phase space. This is the reason why, a priori, this invariant set is not persistent for the full Eqs. 1.1, 1.2, 1.10. As far as the authors know, the existence of Smale horseshoes in Hamiltonian PDEs has been mostly obtained by adding dissipation to the equation which make these sets become fully hyperbolic [3, 5, 13, 30, 33].
- **Beating partially hyperbolic quasiperiodic tori:** The Smale horseshoe obtained in Theorem 1.1 possesses a dense set of periodic orbits. Even if the horseshoe may not persist for the Eqs. 1.1, 1.2, 1.10, KAM Theory should give the persistence of these periodic orbits. In [28], the authors prove the existence of beating KAM Tori. The tori in [28] are elliptic whereas those coming from the horseshoe would be partially elliptic and partially hyperbolic.
- **Non-integrability of** ${\mathcal {N}}|_\Lambda$ **in** (1.7): Theorem 1.1 (and therefore Theorems 1.3 and 1.4) relies on the fact that ${\mathcal {N}}|_\Lambda$ is not integrable and admits invariant tori with transverse homoclinic orbits. On the other hand, the Birkhoff normal form truncation associated to the cubic Nonlinear Schrödinger equation
  1.13 $$\begin{aligned} i u_t=\Delta u-|u|^2 u,\quad x\in {\mathbb {T}}^2 \end{aligned}$$
  is such that ${\mathcal {N}}|_\Lambda$ is integrable. Therefore, the invariant manifolds of the invariant tori coincide and one cannot construct the orbits given in Theorems 1.3 and 1.4 for this equation (at least not with the tools used in the present paper).
- **Unbounded nonlinearities and higher dimensions:** The PDEs analyzed in this paper are semilinear PDEs, namely they have bounded nonlinearities, on the two dimensional torus. However we expect that our results could be extended (with opportune modifications) to models with unbounded nonlinearities and to higher dimensional tori. Usually the unboundedness of the nonlinearity creates problems with the convergence of the normal form transformations. The same issue can arise if one increases the dimension of the spatial domain due to the possible loss of derivatives coming from the small divisors. The reduction to the resonant model ${\mathcal {N}}|_\Lambda$ is obtained by means of a weak version of the Birkhoff normal form procedure, that is not affected by the aforementioned problems of convergence. This method is described in Sect. 3 and it is well established in the KAM theory for quasi-linear PDEs (see for instance [2, 12]).
- **Defocusing and Focusing equations:** To simplify the exposition, the theorems above only refer to the defocusing Eqs. (1.1) and (1.2). However, it can be checked that the sign of the nonlinearity does not play any role and therefore, Theorems 1.1, 1.3 and 1.4 also apply to the focusing equations
  $$\begin{aligned} u_{tt}-\Delta u-u^3=0,\qquad u_{tt}+\Delta ^2 u-u^3=0. \end{aligned}$$

### Transfer of energy and growth of Sobolev norms

The solutions of the Wave equation (1.1)/Beam equation (1.2)/Hartree equation (1.10) obtained in Theorem 1.4 undergo certain transfer of energy between modes. Unfortunately, such transfer of energy does not lead to growth of Sobolev norms [7, 8, 24].

We would like to devote this section to relate our results to that of [8]. In [8], the authors obtain orbits undergoing growth of Sobolev norms for the defocusing nonlinear Schrödinger equation on ${\mathbb {T}}^2$. One of the key points of their proof is to construct, for the Birkhoff normal form truncation, a chain of invariant tori (periodic orbits in certain symplectic reduction, named *toy model*) which are connected by *non-transverse* heteroclinic orbits. To obtain such connections, they strongly rely on the following fact. Even if this toy model is not integrable, it is integrable once restricted to certain invariant subspace (what can be called *two generations model* following [8]). Then the orbits undergoing growth of Sobolev norms are well approximated by orbits which shadow (follow closely) this chain of periodic orbits.

If one wants to use their ideas to obtain similar behavior in other equations such as the Wave (1.1), Beam (1.2) and Hartree (1.10) equations, one has to face several challenges.

First of all, in these equations, the two generations model is not integrable (for the Hartree equation it is not for a generic potential).^4^ This is not surprising. Indeed, typically (at least in finite dimensional Hamiltonian systems) unstable motion (Smale horseshoes, Arnold diffusion) is related to non-integrability. Still, even if non-integrability should “help ” to achieve growth of Sobolev norms it makes the analysis considerably more difficult. The present paper is a first attempt to understand this regime (for the two generations model).

The models we consider are carefully chosen so that they are close to integrable and therefore can be analyzed through perturbative methods. Unfortunately, for the Wave and Beam equation, to be close to integrable we have to choose the modes in $\Lambda$ with very similar modulus and therefore it seems difficult to use the analysis done in this paper to construct orbits undergoing growth of Sobolev norms. For the Hartree equation, one should expect that the ideas developed in this paper could lead to growth of Sobolev norms for a generic potential satisfying (1.11), (1.12).

A second fundamental difference between NLS and the PDEs considered in this paper is about the chain of tori connected by heteroclinic connections considered in [8]. Such structure is not *structurally stable* in the following sense: to have such heteroclinic connections one certainly needs that the connected invariant tori belong to the same level of energy (and to the samel level of other first integrals that the finite dimensional reduction possesses). This does not happen to be the case in other equations besides NLS. Indeed, for the Hartree equation (1.10) with a generic potential *V* the tori considered in [8] belong to different level of energy and the same happens for the Wave and Beam equations for a generic choice of resonant tuples.

Therefore, to achieve growth of Sobolev norms for those equation one certainly needs to consider other invariant objects. The tori considered in Theorem 1.1 are radically different from those in [8]. These tori come in families of higher dimension which are transverse to the first integrals. Moreover, they are indeed connected by heteroclinic orbits. These connections are transverse and, therefore, they are robust. We believe that such objects could play a role if one wants to implement [8] to other PDEs.

## Heuristics and Description of the Paper

The general argument we use in the proofs of Theorems 1.3 and 1.4 follows some of the ideas in the literature [8, 22–24, 27]. The steps are the following. First, a weak Birkhoff normal form procedure simplifies the infinite dimensional Hamiltonian defined by the PDE, removing some non-resonant terms. Second, the normal form is truncated. The truncated normal form admits finite dimensional invariant subspaces. Third, a choice of these subspaces is made, defining a finite dimensional approximation of the PDE, that we call *resonant model*. Some particular finite dimensional orbit of the finite dimensional model is found. Fourth and final, a true solution of the original PDE, close to the finite dimensional one for long enough time, is found. We will use this scheme, with particular choices in each step, particularly when considering the finite dimensional model.

In [8, 27], in the third step, the particular orbit found in the resonant model is obtained relying on the fact that the resonant model is integrable. More precisely, some invariant manifolds of different hyperbolic objects, coincide. Our approach is essentially different, because our resonant model is non-integrable in the sense that the invariant manifolds of several invariant objects—fixed points or periodic orbits—intersect transversally. We take advantage of the non-integrable dynamics of the finite dimensional model to obtain solutions of the truncated normal form with prescribed behavior; indeed, non-integrable dynamics is richer than the integrable one. More details are given below.

As a matter of fact, the proofs of Theorems 1.3 and 1.4 share all these common ingredients and only differ in the finite dimensional phenomena arisen by non-integrability.

Let us give more details concerning our implementation of the strategy. We refer to [18] for further details.

*Step 1: *Each of the PDEs under consideration has a Hamiltonian structure. Let us denote by *H* the Hamiltonian. Given a *complete* (see Definition 3.3) finite subset $\Lambda \subset {\mathbb {Z}}^2$ of resonant modes, to be chosen later, a *weak normal form* scheme is applied to the Hamiltonian. This weak normal form only “removes” a finite number of monomials of degree 4 of the Hamiltonian. Hence it is well defined (the normal form transformation is defined by the flow of a system of ODEs). The monomials to be killed are related to the set $\Lambda$. Although the normal form procedure is not complete and many non-resonant terms of degree 4 are left untouched, for suitable $\Lambda$ the truncated normal form admits a finite dimensional invariant subspace supported on $\Lambda$. This is done in Sect. 3.

Once the Hamiltonian is written in the normal form coordinates, we consider the truncated normal form, disregarding the terms of degree 6 or more. We call this truncated normal form the *resonant model*.

*Step 2: * This step is the core of the paper and can be divided as follows.

- *Construction of the Set* $\Lambda$ (Sect. 4): The set $\Lambda$ is chosen in such a way that its associated subspace of modes (see $V_{\Lambda }$ in (3.11)) is invariant by the flow of the resonant model, but of course satisfies other requirements. Its precise definition depends on the PDE model we consider, but all three instances (Wave, Beam and Hartree equations) of the set $\Lambda$ share some common features. They have exactly 4*N* elements which, using the terminology introduced in [8], encompass two generations (see [29] for the analysis of transfer of energy for a different two generations model given by the quintic NLS). The elements of the set $\Lambda$ are organized in groups of four, pairwise disjoint, each of them forming a parallelogram. The choice of the modes is such that each individual parallelogram is invariant. It also happens that the dynamics of a single parallelogram is integrable, that is, if the rest of the modes are at 0, the dynamics of the four modes in a parallelogram is integrable. At this point is where our choice of the modes differs from other examples in the literature.
- *The dynamics of the finite dimensional model* (Sects. 5, 6, 7): First, we choose our modes in such a way that the dynamics of the resonant model is close to integrable, where closeness to integrability is measured through some parameter $\varepsilon$. The nearly integrability is obtained choosing properly the modes in $\Lambda$. The unperturbed system (where $\varepsilon = 0$) possesses certain invariant objects, namely hyperbolic fixed points and hyperbolic periodic orbits, whose invariant manifolds form heteroclinic or homoclinic separatrices. Our second (generic) condition on the modes is sufficient to ensure that these heteroclinic or homoclinic manifolds split for small $\varepsilon \ne 0$, giving rise to horseshoes and instability phenomena from which we deduce the existence of certain types of orbits. The splitting of these manifolds is measured by means of a suitable set of Melnikov integrals [36]. There is a wide literature on the application of Melnikov Theory to construct homoclinic solutions in PDEs (see, for instance [34, 41, 43]). However, note that in the present paper we apply Melnikov Theory to a finite dimensional model, not to the full PDE.
- *The infinite symbols Smale horseshoe* (Sect. 6): The orbits in Theorem 1.3 give rise from a horseshoe of infinite symbols that can be constructed close to a hyperbolic periodic orbit whose invariant manifolds intersect transversally. The construction of this horseshoe follows the ideas in [37]. The horseshoe can be described as follows. Let $\Gamma =\{1,2,3, \dots \}$ be a denumerable set of symbols and
  2.1 $$\begin{aligned} \Sigma = \{s= (\dots , s_1, s_0, s_1, \dots ) \mid s_i \in \Gamma , \; i \in {{\mathbb {N}}}\}, \end{aligned}$$
  the space of bi-infinite *sequences*, with the product topology. Notice that, unlike what happens when $\Gamma$ is a finite set, $\Sigma$ is not compact. The shift $\sigma : \Sigma \rightarrow \Sigma$ is the homeomorphism on $\Sigma$ defined by $(\sigma (s))_{i} = s_{i-1}$. Following the construction of Moser in [37], given a hyperbolic periodic orbit whose invariant manifolds intersect transversally, it is possible to find a set of coordinates—one of the coordinates is time, in ${\mathbb {T}}$—, a suitable section ${\mathcal {S}}$ that defines a return map $\phi$ and a set *Q* in this section with $\phi (Q) = Q$, such that there exists a homeomorphism $\tau : \Sigma \rightarrow Q$ satisfying $\phi \circ \tau = \tau \circ \sigma$. The set *Q* is in fact the intersection of forward and backward images by $\phi$ of a set of disjoint closed bands $\{{\mathcal {V}}_j, \; j \in {{\mathbb {N}}}\}$, where the index *j* denotes precisely the time between to consecutives passes through ${\mathcal {S}}$ and hence measures the distance to one of the invariant manifolds of the set ${\mathcal {V}}_j$. In this way, ${\mathcal {V}}_j$ tends to the invariant manifold when *j* tends to infinity. The set *Q* is not compact because the return map is not defined in the invariant manifolds.
  The Moser [37] construction implies that the bigger $s_i$ is [see (2.1)], the longer it takes the orbit to come back to $\Sigma$. Since the sequence *s* can be taken randomly, the return times as well.
- *Shadowing of a sequence of periodic orbits* (Sect. 7): the orbits in Theorem 1.4
  travel along a chain of periodic orbits connected by transverse heteroclinic orbits, following the diffusion mechanism described originally by Arnold [1]. This mechanism consists of a sequence - finite or infinite - of partially hyperbolic periodic orbits^5^, $\{{{\mathcal {T}}}_i\}_{i\in I}$, $I\subset {\mathbb {N}}$, such that the unstable manifold of ${{\mathcal {T}}}_i$, $W^u({{\mathcal {T}}}_i)$, intersects *transversally* the stable manifold of ${{\mathcal {T}}}_{i+1}$, $W^s({{\mathcal {T}}}_{i+1})$. Here, since the system we are considering is autonomous, *transversally* means transversality in the energy level, which implies that the intersection of the manifolds is, locally, a single heteroclinic orbit. If a nondegeneracy condition is met, this transversality is sufficient to have a Lambda Lemma that implies that $W^u({{\mathcal {T}}}_{i+1}) \subset \overline{W^u({{\mathcal {T}}}_{i})}$ (see [15]), which in turn implies that for any $i,j \in I$, $i< j$, $W^u({{\mathcal {T}}}_{j}) \subset \overline{W^u({{\mathcal {T}}}_{i})}$. One can then choose arbitrary small neighborhoods of the tori ${{\mathcal {T}}}_i$ and orbits that visit these neighborhoods according to an increasing sequence of times.
  It is worth to remark that the orbits found in the resonant model do exist for any positive time. In the case of the horseshoe with infinite symbols, one obtains orbits that arrive closer and closer to the periodic orbit, in randomly chosen times. In the case of the diffusion orbits, one obtain solutions that wander along the chain of periodic orbits for any positive time, and can be chosen to arrive closer and closer to each periodic orbit.

*Step 3:* The last step of the proof consists in finding a true solution of each PDE shadowing for long enough time the chosen solution of the resonant model. This is accomplished by a standard Gronwall and bootstrap argument. This relies on the Approximation argument given in Sect. 3 with the analysis of the dynamics of the Birkhoff normal form truncation of Sects. 6, 7. In this final step is crucial that the Eqs. (1.1), (1.2) have been restricted to ${\mathbb {Z}}^2_{\mathrm {odd}}$ (see (1.4)) since this implies that the $u=0$ is an elliptic critical point.

## Weak Birkhoff Normal form

### Hamiltonian formalism

In this section we show that the Hamiltonian PDEs (1.1), (1.2) and (1.10) have a Hamiltonian of the same form in an appropriate set of coordinates. We consider spaces of functions defined on ${\mathbb {T}}^2$, hence it is convenient to use the Fourier representation $u(x)=\sum _{j\in {\mathbb {Z}}^2} u_j\,e^{\mathrm {i} j \cdot x}$.

Let us denote by ${\mathcal {P}}$ the phase space and $\Omega$ a symplectic form on it. The vector field $X_H$ of a Hamiltonian *H* is uniquely determined by the formula $d H(u)[\cdot ]=\Omega (X_H(u), \,\cdot )$.

*Hamiltonian structure of equation* (1.10) Let us consider ${\mathcal {P}}:=H^1({\mathbb {T}}^2; {\mathbb {C}})\times H^1({\mathbb {T}}^2; {\mathbb {C}})$ equipped with the symplectic form $\Omega :=\mathrm {i} du \wedge d \bar{u}=\mathrm {i} \sum _{j\in {\mathbb {Z}}^2} d u_j\wedge d\overline{u_j}$. If *V* satisfies (1.11), the equation (1.10) is given by $\partial _tu=X_H(u,{\overline{u}})$ where

3.1 $$\begin{aligned} \begin{aligned} H(u, \bar{u})&=\frac{1}{(2\pi )^2}\left( \int _{{\mathbb {T}}^2} |\nabla u|^2\,dx+ \frac{1}{2}\int _{{\mathbb {T}}^2}(V(x)\star |u |^2)\,|u |^2\,dx\right) \\&=\sum _{j\in {\mathbb {Z}}^2} |j |^2 |u_j |^2+\sum _{j_1-j_2+j_3- j_4=0} V_{j_1-j_2} u_{j_1}\,\overline{u_{j_2}}\, u_{j_3}\,\overline{u_{j_4}}. \end{aligned} \end{aligned}$$

*Hamiltonian structure of equations* (1.1), (1.2) In the following we use the parameter $\kappa \in \{ 1, 2\}$ to treat both cases at the same time. More precisely, $\kappa =1$ if we refer to the Wave equation (1.1) or $\kappa =2$ when we consider the Beam equation (1.2). By setting $v:={\dot{u}}$, we can express these equations as the following system of two first order equations

3.2 $$\begin{aligned} {\left\{ \begin{array}{ll} {\dot{u}}=v,\\ {\dot{v}}=(-1)^{\kappa +1}\Delta ^{\kappa } u-u^3. \end{array}\right. } \end{aligned}$$

We recall the subset ${\mathbb {Z}}^2_{\mathrm {odd}}:=\{ (j^{(1)}, j^{(2)})\in {\mathbb {Z}}^2 \,: \,\, j^{(1)}\,\,\text{ odd }\,\,,\,\,j^{(2)}\,\,\text{ even } \}$. The subspace

3.3 $$\begin{aligned} {\mathcal {U}}_{\mathrm {odd}}:=\left\{ (u, v)\in H^{\kappa }({\mathbb {T}}^2; {\mathbb {R}})\times L^2({\mathbb {T}}^2; {\mathbb {R}}), \,\,u=\sum _{j\in {\mathbb {Z}}^2_{\mathrm {odd}}} u_j\,e^{\mathrm {i}\,j \cdot x}, \,\,v=\sum _{j\in {\mathbb {Z}}^2_{\mathrm {odd}}} v_j\,e^{\mathrm {i}\,j \cdot x} \right\} \end{aligned}$$

is invariant for (3.2). Since $(0, 0)\notin {\mathbb {Z}}^2_{\mathrm {odd}}$ the change of variables $\Xi (u, v)=(\Psi , {\overline{\Psi }})$ defined by

3.4 $$\begin{aligned} \Psi:= & {} \frac{1}{\sqrt{2}} \Big ( |D|^{\kappa /2} u-\mathrm {i} |D|^{-\kappa /2} v \Big ), \qquad {\overline{\Psi }}:=\frac{1}{\sqrt{2}} \Big (|D|^{\kappa /2} u+\mathrm {i} |D|^{-\kappa /2} v \Big ) \nonumber \\ |D |:= & {} (-\Delta )^{1/2}, \end{aligned}$$

is well defined on ${\mathcal {U}}_{{\mathrm {odd}}}$ and it transforms the system (3.2) into the following one

3.5 $$\begin{aligned} {\left\{ \begin{array}{ll} -\mathrm {i}{\dot{\Psi }}=|D|^\kappa \Psi +\frac{1}{4} |D|^{-\kappa /2}\left( \left( |D|^{-\kappa /2}\left( \Psi +{\overline{\Psi }} \right) \right) ^3 \right) \\ \mathrm {i}\dot{{\overline{\Psi }}}=|D|^\kappa {\overline{\Psi }}+\frac{1}{4} |D|^{-\kappa /2}\left( \left( |D|^{-\kappa /2}\left( \Psi +{\overline{\Psi }} \right) \right) ^3 \right) . \end{array}\right. } \end{aligned}$$

The vector field in (3.5) is Hamiltonian with respect to the 2-form $\Omega :=\mathrm {i} d\Psi \wedge d{\overline{\Psi }}$ and Hamiltonian

$$\begin{aligned} H(\Psi ,{\overline{\Psi }}):=\frac{1}{(2\pi )^2}\,\left[ \int _{{\mathbb {T}}^2} |D|^{\kappa } \Psi \,{\overline{\Psi }}\,dx+\frac{1}{4}\int _{{\mathbb {T}}^2} \left( |D|^{-\kappa /2}\left( \frac{\Psi +{\overline{\Psi }}}{\sqrt{2}}\right) \right) ^4\,dx \, \right] . \end{aligned}$$

By considering the Fourier expansion $\Psi =\sum _{j\in {\mathbb {Z}}^2} a_j\,e^{\mathrm {i} j\cdot x},$ we can consider $\Omega =\mathrm {i} \sum _{j\in {\mathbb {Z}}^2_{\mathrm {odd}}} d a_j\wedge d \overline{a_j}$ and

3.6 $$\begin{aligned} \begin{aligned} H&=\sum _{j\in {\mathbb {Z}}^2_{\mathrm {odd}}} |j|^{\kappa } \,a_j\,\overline{a_j}+\frac{1}{16} \sum _{\begin{array}{c} j_i\in {\mathbb {Z}}^2_{\mathrm {odd}},\\ j_1+j_2+j_3+j_4=0 \end{array}} \frac{(a_{j_1}+\overline{a_{-j_1}}) (a_{j_2}+\overline{a_{-j_2}}) (a_{j_3}+\overline{a_{-j_3}})(a_{j_4}+\overline{a_{-j_4}}) }{(|j_1| \,|j_2|\,|j_3|\,|j_4|)^{\kappa /2}}. \end{aligned} \end{aligned}$$

We observe that the Hamiltonians (3.1) and (3.6) have the form^6^

3.7 $$\begin{aligned} H=H^{(2)}+H^{(4)}=\sum _{j\in {\mathbb {Z}}^2_*} \omega (j)\, a_j\,\overline{a_j}+\sum _{\begin{array}{c} j_i\in {\mathbb {Z}}^2_*, \sigma _i\in \{ \pm \},\\ \sigma _1 j_1+\sigma _2 j_2+\sigma _3 j_3+\sigma _4 j_4=0 \end{array}} C^{\sigma _1 \sigma _2 \sigma _3 \sigma _4}_{j_1 j_2 j_3 j_ 4}\, a_{j_1}^{\sigma _1}\,a_{j_2}^{\sigma _2}\,a_{j_3}^{\sigma _3}\,a_{j_4}^{\sigma _4}, \end{aligned}$$

where

- (Hartree): ${\mathbb {Z}}^2_*={\mathbb {Z}}^2$, $\omega (j)=|j |^2$, and the coefficients $C^{\sigma _1 \sigma _2 \sigma _3 \sigma _4}_{j_1 j_2 j_3 j_ 4}$ are defined as
  3.8 $$\begin{aligned} C^{+ - + -}_{j_1 j_2 j_3 j_4}=C^{-+ - +}_{j_1 j_2 j_3 j_4}=V_{j_1-j_2}, \qquad C^{\sigma _1 \sigma _2 \sigma _3 \sigma _4}_{j_1 j_2 j_3 j_ 4}=0 \,\,\,\text{ otherwise }. \end{aligned}$$
- ($$\kappa =1$$ Wave, $$\kappa =2$$ Beam): $${\mathbb {Z}}^2_*={\mathbb {Z}}^2_{\mathrm {odd}}$$, $$\omega (j)=|j |^{\kappa }$$ and, for $$(j_1, j_2, j_3, j_4) $$ such that $$\sigma _1 j_1+\sigma _2 j_2+\sigma _3 j_3+\sigma _4j_4=0$$, we have
  3.9 $$\begin{aligned} \begin{aligned}&C^{\sigma _1 \sigma _2 \sigma _3 \sigma _4}_{j_1 j_2 j_3 j_ 4}=\frac{1}{16 \left( |j_1|| j_2 || j_3|| j_ 4| \right) ^{\kappa /2}}. \end{aligned} \end{aligned}$$

We remark that [using (1.12) for the Hartree equation] the coefficients $C^{\sigma _1\sigma _2\sigma _3\sigma _4}_{j_1 j_2 j_3 j_4}$ are such that

3.10 $$\begin{aligned} \sup _{j_1, j_2, j_3, j_4\in {\mathbb {Z}}_*^2} | C^{\sigma _1\sigma _2\sigma _3\sigma _4}_{j_1 j_2 j_3 j_4} |\le 2. \end{aligned}$$

### Weak Birkhoff normal form

In this section we apply a Birkhoff normal form argument to the Hamiltonian (3.7). We consider the symplectic form $\Omega =\mathrm {i} \sum _{j\in {\mathbb {Z}}_*^2} d a_j\wedge d \overline{a_j}$.

We denote by $\mathrm {ad}_{H^{(2)}}$ the adjoint action of the Hamiltonian $H^{(2)}$. If $F=\sum _{\sigma _1 j_1+\dots +\sigma _n j_n=0} F^{\sigma _1\dots \sigma _n}_{j_1\dots j_n} a_{j_1}^{\sigma _1}\dots a_{j_n}^{\sigma _n}$ is a homogenous, momentum preserving Hamiltonian of degree *n* we have then

$$\begin{aligned} \mathrm {ad}_{H^{(2)}}[F]:=\{ H^{(2)}, F \}=\sum _{\sigma _1 j_1+\dots +\sigma _n j_n=0} \left( \sum _{i=1}^n \sigma _i \omega (j_i) \right) F^{\sigma _1\dots \sigma _n}_{j_1\dots j_n} a_{j_1}^{\sigma _1}\dots a_{j_n}^{\sigma _n}. \end{aligned}$$

We denote by $\Pi _{\mathrm {Ker}(H^{(2)})}$ the projection on the kernel of $\mathrm {ad}_{H^{(2)}}$.

#### Definition 3.1

We say that a *n*-tuple $(\sigma _i, j_i)_{i=1}^n$ is a *n*-resonance if

$$\begin{aligned} \sum _{i=1}^n \sigma _i \omega (j_i)=0, \qquad \sum _{i=1}^n \sigma _i \,j_i=0. \end{aligned}$$

Since there are no regularity issues in what follows we decide to work on the phase space of analytic sequences. We fix $\rho >0$ and define

$$\begin{aligned} W_{\rho }:=\left\{ a=(a_j)_{j\in {\mathbb {Z}}^2_*}\in \ell ^1 : \Vert a \Vert _{\rho }:=\sum _{j\in {\mathbb {Z}}^2_*} |a_j |\,e^{\rho |j |} <\infty \right\} . \end{aligned}$$

We denote by ${\mathcal {B}}_{\rho }(\delta )$ the open ball of radius $\delta >0$ centered at the origin of $W_{\rho }$. We use the notation $A \lesssim B$ to denote $A\le C\, B$ where $C>0$ is a constant possibly depending on the fixed $\rho$.

Let $\Lambda$ be a finite subset of ${\mathbb {Z}}_*^2$. We consider the following splitting $W_{\rho }=V_{\Lambda }\oplus Z_{\Lambda }$ with

3.11 $$\begin{aligned} V_{\Lambda }:=V_{\Lambda , \rho }=\left\{ a\in W_{\rho } : a_j=0\,\,\text{ if } \,\,j\notin \Lambda \right\} , \quad Z_{\Lambda }:=Z_{\Lambda , \rho }=\left\{ a\in W_{\rho }: a_j=0\,\,\text{ if } \,\,j\in \Lambda \right\} . \end{aligned}$$

We define

$$\begin{aligned} {\mathcal {S}}_{n, k}:=\left\{ (\sigma _i, j_i)_{i=1}^n :\,\,\sum _{i=1}^n \sigma _i j_i=0\,\,\text{ such } \text{ that } \text{ the } \text{ number } \text{ of } \text{ indices } \,\,j_i\notin \Lambda \,\, \text{ is } \text{ exactly }\,\, k\right\} . \end{aligned}$$

Given a homogenous *n*-degree, momentum preserving Hamiltonian $F=\sum _{\sigma _1 j_1+\dots +\sigma _n j_n=0} F^{\sigma _1\dots \sigma _n}_{j_1\dots j_n} a_{j_1}^{\sigma _1}\dots a_{j_n}^{\sigma _n}$, we denote by $F^{(n, k)}$ the projection of *F* onto the monomials $a^{\sigma _1}_{j_1}\dots a_{j_n}^{\sigma _n}$ with exactly *k* indices $j_i\notin \Lambda$. Thus $F^{(n, k)}$ is the part of the Hamiltonian *F* which is Fourier supported on ${\mathcal {S}}_{n, k}$.

We denote by

$$\begin{aligned} {\mathcal {S}}_{n, \le k}:=\cup _{i=1}^k {\mathcal {S}}_{n, i}, \quad {\mathcal {S}}_{n, \ge k}:=\cup _{i=k}^n {\mathcal {S}}_{n, i}. \end{aligned}$$

We refer to $F^{(n, \le k)}$ (and $F^{(n, \ge k)}$) the part of the Hamiltonian *H* which is Fourier supported on ${\mathcal {S}}_{n, \le k}$ (and ${\mathcal {S}}_{n, \ge k}$).

#### Remark 3.2

Since we assume that $\Lambda$ is finite, the preservation of momentum implies that the Hamiltonians $F^{(n, \le 1)}$ have compact Fourier support.

#### Definition 3.3

We say that a subset $\Lambda \subset {\mathbb {Z}}^2$ is *complete* if the following holds: given a 4-resonance $(\sigma _i, j_i)_{i=1}^4$ we have that if $j_1, j_2, j_3\in \Lambda$ then $j_4\in \Lambda$.

#### Proposition 3.4

(Weak Birkhoff normal form) Fix $\rho >0$. Let $\Lambda \subset {\mathbb {Z}}^2_*$ be finite and complete and consider the Hamiltonian *H* in (3.7). Then,

(i) There exists $\delta _1>0$ small such that $\forall \delta \in (0, \delta _1)$ there exists an analytic change of coordinates $\Gamma :{\mathcal {B}}_{\rho }(\delta )\subset W_\rho \rightarrow {\mathcal {B}}_{\rho }(2\delta )$ such that
  3.12 $$\begin{aligned} H\circ \Gamma =H^{(2)}+\Pi _{\mathrm {Ker}(H^{(2)})}H^{(4, 0)}+H^{(4, \ge 2)}+{\mathcal {R}} \end{aligned}$$
  where $${\mathcal {R}}$$ satisfies
  $$\begin{aligned} \Vert X_{{\mathcal {R}}}(a) \Vert _{\rho }\lesssim \Vert a \Vert _{\rho }^5\,\qquad \text { for all }\quad a\in {\mathcal {B}}_{\rho }(\delta ). \end{aligned}$$
(ii) Moreover, the map $\Gamma$ is close to the identity, i.e. $\Vert \Gamma (a)-a\Vert _{\rho }\lesssim \Vert a \Vert _{\rho }^3\,$ for all $a\in {\mathcal {B}}_{\rho }(\delta )$.

#### Proof

Let us consider the 4-degree homogenous Hamiltonian

$$\begin{aligned} F=\sum _{\begin{array}{c} (\sigma _i, j_i)\in {\mathcal {S}}_{4, \le 1},\\ \sigma _1 j_1+\sigma _2 j_2+\sigma _3 j_3+\sigma _4 j_4=0 \end{array}} F_{j_1 j_2 j_3 j_4}^{\sigma _1 \sigma _2 \sigma _3 \sigma _4} \,a^{\sigma _1}_{j_1}\,a^{\sigma _2}_{j_2}\,a^{\sigma _3}_{j_3}\,a^{\sigma _4}_{j_4} \end{aligned}$$

where

3.13 $$\begin{aligned} F_{j_1 j_2 j_3 j_4}^{\sigma _1 \sigma _2 \sigma _3 \sigma _4}=\left\{ \begin{aligned}\displaystyle&-\frac{\mathrm {i} C^{\sigma _1 \sigma _2 \sigma _3 \sigma _4}_{j_1 j_2 j_3 j_ 4}}{\sigma _1\omega (j_1)+\sigma _2\omega (j_2)+\sigma _3\omega (j_3)+\sigma _4\omega (j_4)}&\sigma _1\omega (j_1)+\sigma _2\omega (j_2)+\sigma _3\omega (j_3)+\sigma _4\omega (j_4) \ne 0\\&0&\sigma _1\omega (j_1)+\sigma _2\omega (j_2)+\sigma _3\omega (j_3)+\sigma _4\omega (j_4) = 0. \end{aligned}\right. \end{aligned}$$

The function *F* solves the homological equation

3.14 $$\begin{aligned} \{ H^{(2)}, F\}+H^{(4, \le 1)}=\Pi _{\mathrm {Ker}(H^{(2)})} H^{(4, \le 1)}. \end{aligned}$$

By Remark 3.2 the vector field generated by *F* has just a finite number of non zero components. Hence $\Phi _F^t$ is the flow of a ODE with a smooth vector field. We call $\Gamma =(\Phi _F^t)_{|_{t=1}}$ the time-one flow map of *F*.

By Remark 3.2 the denominators in (3.13) have a uniform lower bound, hence by (3.10) the coefficients defined in (3.13) are uniformly bounded. Then by Young’s inequality it is easy to see that $\Vert X_F(a) \Vert _{\rho }\lesssim \Vert a \Vert ^3_{\rho }$ for all $a\in W_{\rho }$.

This implies that for $\delta >0$ small enough $\Vert \Phi _F^t(a)\Vert _{\rho }\le 2 \Vert a \Vert _{\rho }$ for all $t\in [0, 1]$. Thus $\Gamma$ maps ${\mathcal {B}}_{\rho }(\delta )$ to ${\mathcal {B}}_{\rho }(2\delta )$ and

$$\begin{aligned} \Vert \Gamma (a)-a \Vert _{\rho }\le \sup _{s\in [0, 1]}\Vert X_F(\Phi _F^s(a)) \Vert _{\rho }\le \sup _{s\in [0, 1]} \Vert \Phi _F^s(a) \Vert ^3_{\rho }\lesssim \Vert a \Vert _{\rho }^3. \end{aligned}$$

So we have proved item (ii). After the change of coordinates $\Gamma$ the Hamiltonian (3.7) transforms into

$$\begin{aligned} H\circ \Gamma&=H+\{ H, F\}+\int _0^1 (1-t) \{\{ H, F\}, F\}\circ \Phi ^t_F\,dt\\&=H^{(2)}+\Big (H^{(4, \le 1)}+ \{ H^{(2)}, F \}\Big )+H^{(4, \ge 2)}+\{ H^{(4)}, F \}+\int _0^1 (1-t) \{\{ H, F\}, F\}\circ \Phi ^t_F\,dt.\\&{\mathop {=}\limits ^{ (3.14)}}H^{(2)}+\Pi _{\mathrm {Ker}(H^{(2)})} \,H^{(4, \le 1)}+H^{(4, \ge 2)}+\{H^{(4)}, F\}+\int _0^1 (1-t) \{\{ H, F\}, F\}\circ \Phi ^t_F\,dt. \end{aligned}$$

Then, the completeness of $\Lambda$ implies that

$$\begin{aligned} \Pi _{\mathrm {Ker}(H^{(2)})}H^{(4, \le 1)}=\Pi _{\mathrm {Ker}(H^{(2)})}H^{(4, 0)}. \end{aligned}$$

Moreover, we can take ${\mathcal {R}}:=\{H^{(4)}, F\}+\int _0^1 (1-t) \{\{ H, F\}, F\}\circ \Phi ^t_F\,dt$. We observe that $\{ H^{(4)}, F\}$ is a homogenous Hamiltonian of degree 6 . Regarding the integral term, we have that $\Phi ^t_F$ is smooth and $\{\{ H, F \}, F\}$ is the sum of two homogenous Hamiltonians of degree at least 6, hence it is an analytic function on ${\mathcal {B}}_{\rho }(\delta )$ that can be Taylor expanded at $a=0$. The first term of the Taylor expansion of the vector field is a polynomial of degree 5 and the remainder is smaller in a sufficiently small neighborhood of the origin. Again, by the uniform boundness of the coefficients of *H* and *F*, one can obtain the estimate in item (i) by using Young’s inequality. $\quad \square$

Let us consider the time-dependent change of coordinates

3.15 $$\begin{aligned} \Psi (t):\quad \mathtt {a}_j \rightarrow a_j=\mathtt {a}_j\,e^{\mathrm {i} \omega (j)\,t}, \quad \mathtt {a}=(\mathtt {a}_j)_{j\in {\mathbb {Z}}_*^2}\in W_{\rho }. \end{aligned}$$

This change of coordinates leaves resonant monomials unchanged, that is $\Pi _{\mathrm {Ker}(H^{(2)})} F\circ \Psi =\Pi _{\mathrm {Ker}(H^{(2)})} F$. Then, we have that

3.16 $$\begin{aligned} \begin{aligned}&H\circ \Gamma \circ \Psi =H_{\mathrm {Res}}+{\mathcal {R}}'(t), \qquad H_{\mathrm {Res}}:=\Pi _{\mathrm {Ker}(H^{(2)})} \,H^{(4, 0)}+{\mathcal {Q}}(t) \\&{\mathcal {Q}}:=H^{(4, \ge 2)}\circ \Psi (t), \quad {\mathcal {R}}'={\mathcal {R}}\circ \Psi (t). \end{aligned} \end{aligned}$$

Moreover, the functions ${\mathcal {Q}}$ and ${\mathcal {R}}'$ satisfy

3.17 $$\begin{aligned} \Vert X_{{\mathcal {Q}}}(a) \Vert _{\rho }\lesssim \Vert a \Vert _{\rho }^3 \qquad \text { and }\qquad \Vert X_{{\mathcal {R}}'}(a) \Vert _{\rho }\lesssim \Vert a \Vert _{\rho }^5\,\qquad \text { for all }\quad a\in {\mathcal {B}}_{\rho }(\delta ). \end{aligned}$$

Now, if one considers a complete set $\Lambda$ (see Definition 3.3), the associated subspace $V_{\Lambda }$ [see (3.11)] is left invariant by $X_{H_{\mathrm {Res}}}$. Moreover, on $V_\Lambda$, $X_{H_{\mathrm {Res}}}=X_{\Pi _{\mathrm {Ker}(H^{(2)})} \,H^{(4, 0)}}$. This Hamiltonian is scaling invariant in the sense that if *r*(*t*) is a trajectory of this vector field

3.18 $$\begin{aligned} r^\delta (t)=\delta r(\delta ^2 t) \end{aligned}$$

also is. Taking $\delta \ll 1$, in certain time scales, this trajectory $r^\delta (t)$ stays close to the trajectory of the Hamiltonian (3.16) with the same initial condition.

#### Proposition 3.5

Let $T_0$ be a positive number and consider a solution *r*(*t*) of the Hamiltonian system $H_{\mathrm {Res}}$ in (3.16) such that it is defined for $t\in [0,T_0]$ and $r(0)\in V_{\Lambda }$.

Then there exists $\delta _2=\delta _2(T_0)\le \delta _1$ (where $\delta _1$ is given in Proposition 3.4) such that the following holds: for all $0<\delta \le \delta _2$ the rescaled solution $r^\delta$ of the Hamiltonian $H_{\mathrm {Res}}$ given by (3.18) and the solution *u*(*t*) of the Hamiltonian system $H_{\mathrm {Res}}+{\mathcal {R}}'$ with initial condition $u(0)=r^{\delta }(0)=\delta r(0)$ satisfy

3.19 $$\begin{aligned} \Vert r^{\delta }(t)-u(t) \Vert _{\rho }\lesssim \delta ^2 \qquad \forall t\in [0, \delta ^{-2} T_0]. \end{aligned}$$

#### Proof

We define $\xi :=u-r^{\delta }$. Then, $\xi$ satisfies

$$\begin{aligned} {\dot{\xi }}={\mathcal {Z}}_0(t)+{\mathcal {Z}}_1(t)\xi +{\mathcal {Z}}_{21}(t, \xi )+{\mathcal {Z}}_{22}(t, \xi ) \end{aligned}$$

where

$$\begin{aligned}&{\mathcal {Z}}_0(t):=X_{{\mathcal {R}}'}(r^{\delta }(t)),\\&{\mathcal {Z}}_1(t)=D X_{H_{\mathrm {Res}}}(r^{\delta }(t)),\\&{\mathcal {Z}}_{21}(t, \xi ):=X_{H_{\mathrm {Res}}}(r^{\delta }(t)+\xi (t))-X_{H_{\mathrm {Res}}}(r^{\delta }(t))-DX_{H_{\mathrm {Res}}}(r^{\delta }(t))\xi ,\\&{\mathcal {Z}}_{22}(t, \xi ):=X_{{\mathcal {R}}'}(r^{\delta }(t)+\xi (t))-X_{{\mathcal {R}}'}(r^{\delta }(t)). \end{aligned}$$

By the estimate in item (i) of Proposition 3.4, the fact that $H_{\mathrm {Res}}$ is a homogenous Hamiltonian of degree 4 and (3.17) we have the following estimates

3.20 $$\begin{aligned} \begin{aligned}&\Vert {\mathcal {Z}}_0(t) \Vert _{\rho }\lesssim \Vert r^{\delta } \Vert _{\rho }^5 , \quad \Vert {\mathcal {Z}}_1(t) \Vert _{\rho }\lesssim \Vert r^{\delta } \Vert _{\rho }^2 \Vert \xi \Vert _{\rho },\\&\Vert {\mathcal {Z}}_{21}(t, \xi ) \Vert _{\rho }\lesssim \Vert r^{\delta } \Vert _{\rho } \Vert \xi \Vert ^2_{\rho }+\Vert \xi \Vert ^3_{\rho }, \qquad \Vert {\mathcal {Z}}_{22}(t, \xi ) \Vert _{\rho }\lesssim \Vert r^{\delta } \Vert ^4_{\rho } \Vert \xi \Vert _{\rho }. \end{aligned} \end{aligned}$$

Now we use a bootstrap argument to conclude the proof. We assume temporarily that $\Vert \xi (t) \Vert \lesssim \delta ^2$ for $t\in [0, \delta ^{-2} T_0]$. We already know that this is true for $t=0$ since $\xi (0)=0$. Then by Minkowsky inequality, the fact that $\Vert r^{\delta } \Vert _{\rho }\lesssim \delta$ and (3.20) we have that

3.21 $$\begin{aligned} \frac{d}{d t}\Vert \xi \Vert _{\rho }\le \Vert {\mathcal {Z}}_0(t) \Vert _{\rho }+\Vert {\mathcal {Z}}_1(t)\xi \Vert _{\rho }+\Vert {\mathcal {Z}}_{21}(t, \xi )\Vert _{\rho }+\Vert {\mathcal {Z}}_{22}(t, \xi )\Vert _{\rho } \lesssim \delta ^5+\delta ^2 \Vert \xi \Vert _{\rho }. \end{aligned}$$

Thus integrating (3.21) and by using Gronwall lemma we get

$$\begin{aligned} \Vert \xi (t) \Vert _{\rho }\lesssim \delta ^5 t+\delta ^7 e^{\delta ^2 t} \int _0^t s\,e^{-\delta ^2\,s}\,ds= \delta ^5 t+\delta ^3 e^{\delta ^2 t} (1-e^{-\delta ^2 t}(1+\delta ^2 t)) \end{aligned}$$

and since $0<t\le \delta ^{-2} T_0$ we have

$$\begin{aligned} \Vert \xi (t) \Vert _{\rho }\lesssim \delta ^3 T_0+\delta ^3 e^{ T_0}. \end{aligned}$$

Since $T_0$ is independent from $\delta$, we can choose $\delta$ small enough such that $\Vert \xi (t) \Vert _{\rho }\lesssim \delta ^{5/2}$ for $t\in [0, \delta ^{-2} T_0]$. Since this bound is stronger than the bootstrap assumption we can drop such hypothesis and the proof is concluded. $\quad \square$

## Reduction to the Resonant Model

### Lambda set

We introduce a suitable finite and complete (see Definition 3.3) resonant set of modes $\Lambda \subset {\mathbb {Z}}^2$, whose construction is based on the ideas of [8]. This set is constructed such that the associated subspace

4.1 $$\begin{aligned} V_{\Lambda }:=\{ a\in W_{\rho } : a_j=0 \quad \forall j\notin \Lambda \} \end{aligned}$$

is invariant under the flow associated to the Hamiltonian $H_\mathrm {Res}$ in (3.16). Later on we study the dynamics of the Hamiltonian $H_{\mathrm {Res}}$ restricted to initial data supported on $V_{\Lambda }$.

First we introduce the set of resonant tuples for the nonlinear Beam equation (those of the Hartree equation are a subset of it),

4.2 $$\begin{aligned} {\varvec{A}}_{bh}&:= \left\{ (n_1,n_2,n_3,n_4) \in ({\mathbb {Z}}^2)^4: n_1 \pm n_2\pm n_3\pm n_4=0, |n_1|^2\pm |n_2|^2\pm |n_3|^2\pm |n_4|^2=0 \right\} , \end{aligned}$$

and a subset of it, which is going to be used to build the set $\Lambda$,

4.3 $$\begin{aligned} {{\widetilde{{\varvec{A}}}}}_{bh}&:= \left\{ (n_1,n_2,n_3,n_4) \in ({\mathbb {Z}}^2)^4: n_1-n_2+n_3-n_4=0, |n_1|^2-|n_2|^2+|n_3|^2-|n_4|^2=0 \right\} . \end{aligned}$$

Analogously, one can define the resonant tuples for the Wave equation and the corresponding associated subset

4.4 $$\begin{aligned} \begin{aligned} {\varvec{A}}_w&:= \{ (n_1,n_2,n_3,n_4) \in ({\mathbb {Z}}^2)^4: n_1\pm n_2\pm n_3\pm n_4=0, |n_1|\pm |n_2|\pm |n_3|\pm |n_4|=0 \},\\ {{\widetilde{{\varvec{A}}}}}_w&:= \{ (n_1,n_2,n_3,n_4) \in ({\mathbb {Z}}^2)^4: n_1-n_2+n_3-n_4=0, |n_1|-|n_2|+|n_3|-|n_4|=0 \}. \end{aligned} \end{aligned}$$

We consider sets $\Lambda$ whose modes form resonant tuples in ${{\widetilde{{\varvec{A}}}}}_{bh}/{{\widetilde{{\varvec{A}}}}}_{w}$. One could choose the resonant tuples in the bigger sets ${\varvec{A}}_{bh}/{\varvec{A}}_{w}$. However, the particular form of the tuples in ${{\widetilde{{\varvec{A}}}}}_{bh}/{{\widetilde{{\varvec{A}}}}}_{w}$ simplifies the analysis of the dynamics in $V_{\Lambda }$. Indeed, the Hamiltonian $H_\mathrm {Res}$ restricted to $V_{\Lambda }$ will have first integrals which correspond to the mass associated to each resonant tuple (see Sect. 4.3).

Let $N \ge 2$ be an integer, and let ${\varvec{A}}$ be either ${\varvec{A}}_{bh}$ or ${\varvec{A}}_w$ (analogously be ${{\widetilde{{\varvec{A}}}}}$ either ${{\widetilde{{\varvec{A}}}}}_{bh}$ or ${{\widetilde{{\varvec{A}}}}}_w$). We define a set $\Lambda \subset {\mathbb {Z}}^2$ which consists of two disjoint generations, $\Lambda = \Lambda _1 \cup \Lambda _2$, $|\Lambda _1|=|\Lambda _2|=2N$. Define a *nuclear family* to be a set $(n_1, n_2, n_3, n_4) \in {{\widetilde{{\varvec{A}}}}}$ whose elements are ordered, such that $n_1$ and $n_3$ (known as the *parents*) belong to the first generation $\Lambda _1$, and $n_2$ and $n_4$ (known as the *children*) belong to the second generation $\Lambda _{2}$. Note that if $(n_1, n_2, n_3, n_4)$ is a nuclear family, then so are $(n_1, n_4, n_3, n_2)$, $(n_3, n_2, n_1, n_4)$ and $(n_3, n_4, n_1, n_2)$. These families are called trivial permutations of the family $(n_1, n_2, n_3, n_4)$. The first conditions to impose on the set $\Lambda$ were already imposed in the paper [8].

- $1_{\Lambda }$ (*Closure*) If $n_1, n_2, n_3 \in \Lambda$ and there exists $n\in {\mathbb {Z}}^2$ such that $(n_1, n_2, n_3, n) \in {{\widetilde{{\varvec{A}}}}}$ (or any permutation of it), then $n \in \Lambda$. In other words, if three members of a nuclear family are in $\Lambda$, so is the fourth one. This is a rephrasing of the completeness condition (see Definition 3.3).
- $2_{\Lambda }$ (*Existence and uniqueness of spouse and children*) For any $n_1 \in \Lambda _1$, there exists a unique nuclear family $(n_1, n_2, n_3, n_4)$ (up to trivial permutations) such that $n_1$ is a parent of this family. In particular, each $n_1 \in \Lambda _1$ has a unique spouse $n_3 \in \Lambda _1$ and has two unique children $n_2, n_4 \in \Lambda _{2}$ (up to permutation).
- $3_{\Lambda }$ (*Existence and uniqueness of sibling and parents*) For any $n_2 \in \Lambda _{2}$, there exists a unique nuclear family $(n_1, n_2, n_3, n_4)$ (up to trivial permutations) such that $n_2$ is a child of this family. In particular each $n_2 \in \Lambda _{2}$ has a unique sibling $n_4 \in \Lambda _{2}$ and two unique parents $n_1, n_3 \in \Lambda _1$ (up to permutation).
- $4_{\Lambda }$ (*Faithfulness*) Apart from the nuclear families, $\Lambda$ does not contain any other set $(n_1,n_2,n_3,n_4)\in {\varvec{A}}$.

Note that the resonant tuples in $\Lambda$ belong to ${{\widetilde{{\varvec{A}}}}}$. However, condition $4_{\Lambda }$ requires that no other resonant tuples in the (larger set) ${\varvec{A}}$ are possible in $\Lambda$.

In the next two propositions we construct a set $\Lambda$ for the three considered PDEs. In some of the cases we need further conditions. Recall that the Eqs. (1.1), (1.2) have been restricted to ${\mathbb {Z}}^2_{\mathrm {odd}}$ (see (1.4)) so that $u=0$ is an elliptic critical point. Therefore, for these equations the set $\Lambda$ is constructed in ${\mathbb {Z}}^2_{\mathrm {odd}}$.

#### Proposition 4.1

Let $N \ge 2$ and take ${\varvec{A}}={\varvec{A}}_{bh}$. Then there exists a set $\Lambda \subset {\mathbb {Z}}^2$, with $\Lambda = \Lambda _1 \cup \Lambda _2$ and $|\Lambda _j|=2N$, which satisfies properties $1_{\Lambda }$–$4_{\Lambda }$ and the following additional property:

any $n_k,n_{k}',n_h,n_h'\in \Lambda$ such that $n_k \ne n_h$ and $n_k' \ne n_h'$ satisfy

4.5 $$\begin{aligned} n_k - n_h \ne n_{k}' - n_{h}'. \end{aligned}$$

#### Proposition 4.2

Let $N \ge 2$ and take ${\varvec{A}}={\varvec{A}}_{w}, {\varvec{A}}_{bh}$. Then there exists a set $\Lambda \subset {\mathbb {Z}}_\mathrm {odd}^2$, with $\Lambda = \Lambda _1 \cup \Lambda _2$ and $|\Lambda _j|=2N$, which satisfies conditions $1_{\Lambda }$–$4_{\Lambda }$ and the following additional condition. Take any $n,n'\in \Lambda$, then

4.6 $$\begin{aligned} |n| \ne |n'|. \end{aligned}$$

Moreover, if one takes $0<\varepsilon \ll 1$, there exists $R=R(\varepsilon )\gg 1$ so that $\Lambda$ can be chosen to satisfy also

4.7 $$\begin{aligned} \left| |n| - R \right| < R\varepsilon , \; \; \text { for all }\quad n \in \Lambda . \end{aligned}$$

Let us make some comments on the extra conditions imposed on $\Lambda$ in these propositions. Condition (4.6) below is required to apply Melnikov Theory in Sect. 5. Condition (4.7) is used to obtain Hamiltonian systems on $V_{\Lambda }$ [see (4.1)] which are close to integrable for the Beam and Wave equations. For the Beam and Wave equation we also require that the first component of the modes in $\Lambda$ is odd. This is fundamental in the approximation argument (Proposition 3.5) to avoid interactions with the mode $n=0$ which is not elliptic.

We defer the proof of the above propositions to the Appendix 7.3.

#### Lemma 4.3

Consider the Hamiltonian (3.7) given by the Eqs. (1.10), (1.2), (1.1) and the associated $H_{\mathrm {Res}}$ in (3.16) and the set $\Lambda$ obtained in Propositions 4.1 and 4.2. Then $V_{\Lambda }$ is invariant and the restriction of $H_{\mathrm {Res}}$ to $V_{\Lambda }$ [see (4.1)] has the following form

4.8 $$\begin{aligned} \begin{aligned}&\left. (H_{\mathrm {Res}})\right| _{{V_{\Lambda }}}(\{\mathtt {a}_{n}\}_{n\in \Lambda }) =\frac{3}{8}\sum _{\begin{array}{c} j_i\in \Lambda ,\\ j_1-j_2+j_3-j_4=0,\\ |j_1|^{\kappa }-|j_2|^{\kappa }+|j_3|^{\kappa }-|j_4|^{\kappa }=0 \end{array}} C_{j_1\dots j_4}\, \mathtt {a}_{j_1}\,\overline{\mathtt {a}_{j_2}}\,\mathtt {a}_{j_3}\,\overline{\mathtt {a}_{j_4}}\\&\quad = \frac{3}{8}\sum _{n\in \Lambda } C_{nnnn}\,|\mathtt {a}_n |^4+ \frac{3}{4}\sum _{i\ne j, n_i, n_j\in \Lambda } C_{n_i n_j n_i n_j} |\mathtt {a}_{n_i} |^2\,|\mathtt {a}_{n_j} |^2 \\&\quad \quad + \frac{3}{4}\sum _{k=1}^N \,(C_{n_{4k-3} n_{4k-2} n_{4k-1} n_{4k}}+C_{n_{4k-3} n_{4 k} n_{4k-1} n_{4k-2}}\\&\quad \quad +C_{n_{4k-1} n_{4k-2} n_{4k-3} n_{4 k}}+C_{n_{4k-1} n_{4 k} n_{4k-3} n_{4k-2}}) \\&\quad \quad \times \,{\mathrm {Re}}(\mathtt {a}_{n_{4k-3}}\,\overline{\mathtt {a}_{n_{4k-2}}}\,\mathtt {a}_{n_{4k-1}}\,\overline{\mathtt {a}_{n_{4 k}}}) \end{aligned} \end{aligned}$$

with $C_{j_1 j_2 j_3 j_4}=C^{+ - + -}_{j_1 j_2 j_3 j_4}$ [see (3.7), (3.8), (3.9)], namely

$$\begin{aligned} \begin{aligned} \kappa&=2, \qquad C_{j_1 j_2 j_3 j_4}= & {} \,V_{j_1-j_2}=1+{\mathcal {O}}(\varepsilon )\qquad \text {(Hartree)}\\ \kappa&=1, \qquad C_{j_1 j_2 j_3 j_4}= & {} \,\frac{1}{16\sqrt{|j_1 ||j_2 ||j_3 ||j_4 |}}=\frac{1}{R^2}\left( 1+{\mathcal {O}}(\varepsilon )\right) \qquad \text {(Wave)}\\ \kappa&=2,\qquad C_{j_1 j_2 j_3 j_4}= & {} \,\frac{1}{16|j_1 ||j_2 ||j_3 ||j_4 |}=\frac{1}{R^4}\left( 1+{\mathcal {O}}(\varepsilon )\right) \qquad \text {(Beam)}. \end{aligned} \end{aligned}$$

Therefore, these coefficients satisfy $C_{j_1 j_2 j_3 j_4}\ne 0$.

#### Proof

The particular form of Hamiltonian $(H_{\mathrm {Res}})_{|_{V_{\Lambda }}}$ is a direct consequence of the Properties $1_\Lambda$–$4_\Lambda$ satisfied by the set $\Lambda$ and the definition of $H_\mathrm {Res}$ in (3.16). The definition of the coefficients $C_{j_1 j_2 j_3 j_4}$ is given in (3.8), (3.9) and their estimates are consequence of (1.11), (1.12) and (4.7). $\quad \square$

We use the symmetries of the Hamiltonian (4.8) to remove some of the monomials by a gauge transformation. Indeed, since the mass $M:=\sum _{n\in \Lambda } |\mathtt {a}_n |^2$ is a conserved quantity for $(H_{\mathrm {Res}})_{|_{V_{\Lambda }}}$, we can consider the change of coordinates and time reparametrization

4.9 $$\begin{aligned} \alpha _n=\mathtt {a}_n\,e^{\mathrm {i} G t}\qquad \text {and}\qquad t=-(8/3)\mathtt {g}\,\tau \qquad \text {with}\qquad G= \frac{3}{4}\,\mathtt {g}\,M, \end{aligned}$$

for some $\mathtt {g}\in {\mathbb {R}}$ to be chosen. The new system is Hamiltonian with respect to

4.10 $$\begin{aligned} \begin{aligned}&{\widetilde{H}}_{\mathrm {Res}}(\{\alpha _{n}\}_{n\in \Lambda })\\&\quad :=\sum _{n\in \Lambda } |\alpha _{n} |^4+ \sum _{n\in \Lambda } |\alpha _{n} |^4 \left( 1-\frac{C_{n\, n\, n\, n}}{\mathtt {g}}\right) +2\sum _{n_i, n_j\in \Lambda , \,i\ne j} |\alpha _{n_i} |^2\,|\alpha _{n_j} |^2 \left( 1-\frac{C_{n_i n_j n_i n_j}}{\mathtt {g}}\right) \\&\quad \quad -\frac{2}{\mathtt {g}}\,\sum _{k=1}^N \,(C_{n_{4k-3} n_{4k-2} n_{4k-1} n_{4k}}+C_{n_{4k-3} n_{4 k} n_{4k-1} n_{4k-2}}\\&\quad \quad +C_{n_{4k-1} n_{4k-2} n_{4k-3} n_{4 k}}+C_{n_{4k-1} n_{4 k} n_{4k-3} n_{4k-2}}) \\&\quad \quad \times \,{\mathrm {Re}} (\alpha _{n_{4k-3}}\,{\overline{\alpha }}_{n_{4k-2}}\,\alpha _{n_{4k-1}}\,{\overline{\alpha }}_{n_{4 k}}). \end{aligned} \end{aligned}$$

Choosing the constant $\mathtt {g}$ in (4.9) as

4.11 $$\begin{aligned} \mathtt {g}=\,1\qquad \text {(Hartree)},\qquad \mathtt {g}=\,\frac{1}{R^2}\qquad \text {(Wave)}\qquad \mathtt {g}=\,\frac{1}{R^4}\qquad \text {(Beam)}, \end{aligned}$$

then the Hamiltonian system (4.10) takes the following form

4.12 $$\begin{aligned} \begin{aligned} {\widetilde{H}}_{\mathrm {Res}}(\alpha _{n_1}, \dots , \alpha _{ n_{2N} })=&\,\sum _{k=1}^{4N} |\alpha _{n_k} |^4\,+2\,\varepsilon \sum _{1 \le i,j \le 4N} A_{i , j} \,|\alpha _{n_i} |^2\,|\alpha _{n_j} |^2\\&\,- 8 \, \sum _{h=1}^N \mathtt {C}_h \,\text{ Re }( \alpha _{n_{4h-3}} \, \overline{ \alpha _{n_{4h-2}} } \, \alpha _{n_{4h-1}}\, \overline{ \alpha _{n_{4h} } } ), \end{aligned} \end{aligned}$$

where $A = (A_{i , j}) \in {\mathbb {R}}^{4N \times 4N}$ is a symmetric matrix given by

4.13 $$\begin{aligned} \varepsilon A_{j, j}:=\frac{1}{2}-\frac{C_{n_j n_j n_j n_j}}{2 \mathtt {g}}, \qquad \varepsilon A_{i, j}:=1-\frac{C_{n_i n_j n_i n_j}}{\mathtt {g}} \quad i\ne j, \end{aligned}$$

and $$(\mathtt {C}_h)_{h=1,\ldots ,N}$$ satisfies

$$\begin{aligned} \mathtt {C}_{h}=1+{\mathcal {O}}( \varepsilon ), \; \; \forall \, h=1, \dots , N. \end{aligned}$$

The equations of motion read as

4.14 $$\begin{aligned} \mathrm {i}\,\,{\dot{\alpha }}_{n_k}=\left( 2|\alpha _{n_k} |^2 + 2\varepsilon \sum _{1 \le r \le 2N} A_{k , r} |\alpha _{n_r} |^2\right) \,\alpha _{n_k}-8\,\mathtt {C}_h \,\alpha _{n_i}\,\overline{\alpha _{n_l}}\,\alpha _{n_j}, \end{aligned}$$

where $k, i, l, j\in \{4(h-1)+1, 4(h-1)+2, 4(h-1)+3, 4h\}$ give the four modes forming a resonant tuple, $l+k$ is even (namely, following [8], $n_k$ and $n_l$ belong to the same generation) and $h \in \{1,\ldots ,N\}$.

### Invariant subspaces and first integrals of the resonant model

The system associated to Hamiltonian ${\widetilde{H}}_{\mathrm {Res}}$ in (4.12) has large dimension and it is not integrable. Nevertheless, the properties of the set $\Lambda$ and the particular form of the Hamiltonian ${\widetilde{H}}_{\mathrm {Res}}$ ensure that the system associated to ${\widetilde{H}}_{\mathrm {Res}}$ has several invariant subspaces, where one can easily analyze the dynamics. We devote this section to analyze these invariant subspaces and the first integrals of ${\widetilde{H}}_{\mathrm {Res}}$.

Let us split $\Lambda$ both as $\Lambda =\Lambda _1\cup \Lambda _2={{\mathcal {R}}}_1\cup \ldots \cup {{\mathcal {R}}}_N$. The first splitting refers to the two generations and the second refers to the *N* four–wave resonances used to define $\Lambda$ [see (4.2) and (4.4)].

Associated to this set we can consider the following invariant subspaces [recall (4.1)]

$$\begin{aligned} V_{\Lambda _i}=\left\{ \alpha \in V_{\Lambda }: \alpha _j=0\, \text { for }\, j\not \in \Lambda _i\right\} \end{aligned}$$

and, for $$\{i_1,\ldots ,i_k\}\subset \{1,\ldots , N\}$$, $$1<k<N$$,

$$\begin{aligned} V_{i_1,\ldots ,i_k}=\left\{ \alpha \in V_{\Lambda }: \alpha _j=0\, \text { for }\, j\not \in {{\mathcal {R}}}_{i_1}\cup \ldots \cup {{\mathcal {R}}}_{i_k}\right\} . \end{aligned}$$

One can easily check that all those subspaces are invariant under the flow associated to Eq. (4.14). Let us study the corresponding dynamics.

For $V_{\Lambda _1}$ (and analogously for $V_{\Lambda _2}$) one obtains the equation

$$\begin{aligned} \mathrm {i}\,{\dot{\alpha }}_{n_k}=\left( 2|\alpha _{n_k} |^2 + 2\varepsilon \sum _{1 \le r \le 2N , n_r\in \Lambda _1} A_{k , r} |\alpha _{n_r} |^2\right) \,\alpha _{n_k}\qquad \text { for }\,{\alpha }_{n_k}\in \Lambda _1. \end{aligned}$$

Therefore, on $V_{\Lambda _1}$, $|\alpha _{n_r}|^2$ are constants of motion and the phase space is foliated by invariant tori

4.15 $$\begin{aligned} {\mathbb {T}}_{I_1,\ldots I_k}=\left\{ \alpha \in V_{\Lambda _1}: |\alpha _{n_k}|=I_k\right\} \quad \text{ where } \quad I_j>0, \quad j=1, \dots , k. \end{aligned}$$

It can be checked (see Sect. 4.3 below) that these invariant tori are hyperbolic and thus have stable and unstable invariant manifolds.

The dynamics on $V_{i_1,\ldots ,i_k}$ is given as well by Eq. (4.14) just considering the interactions between the modes in the rectangles ${{\mathcal {R}}}_{i_1}, \ldots {{\mathcal {R}}}_{i_k}$.

Hamiltonian ${\widetilde{H}}_{\mathrm {Res}}$ in (4.12) has the first integrals

4.16 $$\begin{aligned} \begin{aligned} S^{(k,+)}_{i,j}= |\alpha _{n_{4(k-1)+i}} |^2 + |\alpha _{n_{4(k-1)+j}} |^2 \qquad i+j\equiv 1 \,\,(\text{ mod }\,\, 2), \quad i, j\in \{1, 2, 3, 4\}, \,\,\, k \in \{1, \ldots , N \},\\ S^{(k,-)}_{i,j}=|\alpha _{n_{4(k-1)+i}} |^2 - |\alpha _{n_{4(k-1)+j}} |^2 \qquad i+j\equiv 0 \,\,(\text{ mod }\,\, 2), \quad i, j\in \{1, 2, 3, 4\}, \,\,\, k \in \{1, \ldots , N \}. \end{aligned} \end{aligned}$$

These constants of motion are in involution. They are not functionally independent but it can be easily checked that the subset of first integrals

4.17 $$\begin{aligned} S^{(k,-)}_{1,3}, S^{(k,-)}_{2,4}, S^{(k,+)}_{3,4},\quad \,\,\, k \in \{1, \ldots , N \} \end{aligned}$$

is functionally independent in the open set $\{\alpha _{n}\ne 0:n\in \Lambda \}\subset V_{\Lambda }$. Certainly they are not functionally independent on the invariant subspaces $V_{\Lambda _1}, V_{\Lambda _2}$ (and in particular are not functionally independent at the tori ${\mathbb {T}}_{I_1,\ldots I_k}$).

### The symplectic reduction

We use the first integrals (4.17) to perform a symplectic reduction to the Hamiltonian (4.12). It can be applied in the open set $\{\alpha _{n}\ne 0:n\in \Lambda \}\subset V_{\Lambda }$ where the first integrals are functionally independent. In this domain, all modes are different from zero and thus one can consider symplectic polar coordinates $(\theta ,I) \in {\mathbb {T}}^{4N} \times (0,+\infty )^{4N}$, given by

4.18 $$\begin{aligned} \begin{aligned} \alpha _{n_k} = \sqrt{I_k} e^{i \theta _k}. \end{aligned} \end{aligned}$$

In these coordinates the Hamiltonian (4.12) takes the form

4.19 $$\begin{aligned} \begin{aligned} H(\theta , I)&= \langle I, I \rangle +2\varepsilon \, \langle A I, I \rangle \\&\quad -8 \sum _{h=1}^N \mathtt {C}_h \sqrt{ I_{4(h-1)+1} \, I_{4(h-1)+2} \, I_{4(h-1)+3} \, I_{4h} } \,\cos (\theta _{4(h-1)+1}-\theta _{4(h-1)+2}\\ {}&\quad +\theta _{4(h-1)+3}-\theta _{4h}) \end{aligned} \end{aligned}$$

and the symplectic form $\Omega _{|_{V_{\Lambda }}}$ becomes the standard one $d\theta \wedge d I=\sum _{k=1}^{4 N} d\theta _k\wedge d I_k$. The Hamiltonian system (4.19) has 4*N* degrees of freedom. We perform a symplectic reduction that leads to an *N* degrees of freedom system. In particular first we consider the restriction of (4.12) to

4.20 $$\begin{aligned} {\mathcal {V}}:= \bigcap _{k=1}^N \left\{ S^{(k,-)}_{1 , 3} = S^{(k,-)}_{2 ,4}= 0 \right\} , \end{aligned}$$

and then we further reduce it to the manifold

4.21 $$\begin{aligned} {\mathcal {W}}:= \bigcap _{k=1}^N \left\{ S^{(k,+)}_{3,4}=1 \right\} \cap {\mathcal {V}}. \end{aligned}$$

We adopt the following notation: we denote by ${\mathbf {0}}_n$ the null matrix of dimension $n\times n$ and by $\mathrm {I}_n$ the identity matrix of dimension $n\times n$. We consider the symplectic linear change of variable $\Psi :{\mathbb {T}}^{4N} \times {\mathbb {R}}^{4N} \rightarrow {\mathbb {T}}^{4N} \times {\mathbb {R}}^{4N}$ defined by

$$\begin{aligned} \begin{pmatrix} \theta \\ I \end{pmatrix} =\Psi \begin{pmatrix} \phi \\ J \end{pmatrix} \end{aligned}$$

with, for $$h=0\ldots N-1$$,

$$\begin{aligned} \begin{pmatrix} \theta _{4h+1}\\ \theta _{4h+2}\\ \theta _{4h+3}\\ \theta _{4h+4} \end{pmatrix} =\begin{pmatrix} 1 &{}\quad 0 &{} \quad 0 &{} \quad 0\\ 0 &{} \quad 1 &{} \quad 0 &{} \quad 0\\ -1 &{} \quad 0 &{} \quad 1 &{} \quad 0\\ 0 &{} \quad -1 &{} \quad 0 &{} \quad 1 \end{pmatrix} \begin{pmatrix} \phi _{4h+1}\\ \phi _{4h+2}\\ \phi _{4h+3}\\ \phi _{4h+4} \end{pmatrix} ,\qquad \begin{pmatrix} I_{4h+1}\\ I_{4h+2}\\ I_{4h+3}\\ I_{4h+4} \end{pmatrix} =\begin{pmatrix} 1 &{} \quad 0 &{} \quad 1 &{} \quad 0\\ 0 &{} \quad 1 &{} \quad 0 &{} \quad 1\\ 0 &{} \quad 0 &{} \quad 1 &{} \quad 0\\ 0 &{} \quad 0 &{} \quad 0 &{} \quad 1 \end{pmatrix} \begin{pmatrix} J_{4h+1}\\ J_{4h+2}\\ J_{4h+3}\\ J_{4h+4} \end{pmatrix} \; =: \; \tilde{\mathtt {B}} \begin{pmatrix} J_{4h+1}\\ J_{4h+2}\\ J_{4h+3}\\ J_{4h+4} \end{pmatrix}. \end{aligned}$$

We consider the restriction of the new Hamiltonian $H\circ \Psi$ at the invariant submanifold ${\mathcal {V}}$ defined in (4.20), which corresponds, in the coordinates (4.18), to the subspace

$$\begin{aligned} \{ J_{4k+1}=J_{4k+2}=0 : k=0, \dots , N-1\}. \end{aligned}$$

The new Hamiltonian does not depend on the angles $\{\phi _i\}_{i\in \{4k+1,4k+2 : k=0,\dots , N-1\}}$ and it reads as ndalign*

$$\begin{aligned}&{\mathsf {H}}\left( \{\phi _{4k+3},\phi _{4k+4},J_{4k+3},J_{4k+4}\}_{k=0}^{N-1}\right) = 2 \sum _{h=0}^{N-1} \sum _{k=3}^4 J_{4h+k}^2 \\&\quad +2\varepsilon \, \sum _{0 \le h,h' \le N-1} \sum _{\begin{array}{c} i=3, 4 \\ k=3, 4 \end{array}} (\mathtt {B}^T A \mathtt {B})_{4h+i, 4h'+k} J_{4h+i} \, J_{4h'+k} \\&\quad -8 \sum _{h=0}^{N-1} \mathtt {C}_{h+1} \,J_{4h+3}\,J_{4h+4}\,\cos (\phi _{4h+3}-\phi _{4h+4}), \end{aligned}$$

where

$$\begin{aligned} \mathtt {B}&= \begin{pmatrix} \tilde{\mathtt {B}} &{}\quad {{\mathbf {0}}}_4 &{}\quad \cdots &{} \quad \cdots &{} \quad {{\mathbf {0}}}_4 \\ {{\mathbf {0}}}_4 &{}\quad \tilde{\mathtt {B}} &{} \quad {{\mathbf {0}}}_4 &{} \quad \cdots &{} \quad {{\mathbf {0}}}_4 \\ \vdots &{}\quad {{\mathbf {0}}}_4 &{} \quad \tilde{\mathtt {B}} &{}\quad {{\mathbf {0}}}_4 &{}\quad \cdots \\ \vdots &{} \quad \vdots &{}\quad \ddots &{} \quad \ddots &{} \quad \vdots \\ {{\mathbf {0}}}_4 &{} \quad \cdots &{}\quad \cdots &{} \quad \cdots &{} \quad \tilde{\mathtt {B}} \end{pmatrix}\in {\mathbb {R}}^{4N \, \times \, 4N}. \end{aligned}$$

The second symplectic reduction is obtained by considering the symplectic linear change of variable $\Phi :{\mathbb {T}}^{2N} \times {\mathbb {R}}^{2N} \rightarrow {\mathbb {T}}^{2N} \times {\mathbb {R}}^{2N}$ as

$$\begin{aligned} \left( \{\phi _{4k+3}\}_{k=0}^{N-1},\{\phi _{4k+4}\}_{k=0}^{N-1}, \{J_{4k+3}\}_{k=0}^{N-1}, \{J_{4k+4}\}_{k=0}^{N-1}\right) =\Phi (\{K_k\}_{k=1}^N,\{{\tilde{K}}_1\}_{k=1}^N,\{\psi _k\}_{k=1}^N,\{{\tilde{\psi }}_1\}_{k=1}^N)\end{aligned}$$

defined by

$$\begin{aligned} \begin{pmatrix} \phi _{4k+3}\\ \phi _{4k+4} \end{pmatrix}=\begin{pmatrix} 1 &{} \quad 1 \\ 0 &{}\quad 1 \end{pmatrix} \begin{pmatrix} \psi _{k+1}\\ {\tilde{\psi }}_{k+1}\end{pmatrix},\qquad \begin{pmatrix} J_{4k+3}\\ J_{4k+4} \end{pmatrix} =\begin{pmatrix} 1 &{} \quad 0 \\ -1 &{}\quad 1 \end{pmatrix}\begin{pmatrix} K_{k+1}\\ {\tilde{K}}_{k+1} \end{pmatrix},\qquad k=0\ldots N-1. \end{aligned}$$

After the reparametrization of time $t\mapsto -4 \,t$, the restriction of the transformed Hamiltonian ${\mathsf {H}}\circ \Phi$ to the subspace

$$\begin{aligned} {\mathcal {W}}={\mathcal {V}} \bigcap _{k=1}^N \{ S^{(k,+)}_{3,4}=1 \}= \bigcap _{k=1}^N \{ {\tilde{K}}_k=1\} \end{aligned}$$

is given (up to constants) by

4.22 $$\begin{aligned} \begin{aligned}&{\mathcal {H}}(\psi _1, \ldots , \psi _N, K_1, \ldots , K_N)= \sum _{j=1}^N K_j (1-K_j)(1 +2 \,\cos (\psi _j))\\&+\varepsilon \left[ \sum _{j=1}^N a_{j} K_j +\sum _{j=1}^N b_{j} K^2_j + \sum _{i, j=1, i< j}^N d_{i j} K_i\,K_j +\sum _{h=1}^N c_j K_j(1-K_j) \,\cos (\psi _j)\right] \end{aligned} \end{aligned}$$

where the coefficients $a_j$, $b_j$ and $d_j$ can be written in terms of the entries of the matrix *A* in (4.12) in the following way

4.23 $$\begin{aligned} \begin{aligned} a_j := -&\sum _{r=1}^N \Big [ A^{(j, r)}_{1, 2}+A^{(j, r)}_{1, 4}+A^{(j, r)}_{ 2, 3}+A^{(j, r)}_{3, 4}-\big ( A^{(j, r)}_{2, 2}+ A^{(r, j)}_{2, 4}+ A^{(r, j)}_{4, 2}+ A^{(j, r)}_{4, 4} \big )\Big ] \\ b_j := -&\Big [ A^{(j, j)}_{1, 1}+2 A^{(j, j)}_{1, 3}+A^{(j, j)}_{3, 3}-2 \big ( A^{(j, j)}_{1, 2} +A^{(j, j)}_{1, 4}+A^{(j, j)}_{2, 3}+A^{(j, j)}_{3, 4}\big )+A^{(j, j)}_{2, 2}+2 A^{(j, j)}_{2, 4}+A^{(j, j)}_{4, 4}\Big ] \\ d_{i j} := -&\Big [ A^{(i, j)}_{1, 1}+ A^{(i, j)}_{1, 3}+A^{(i, j)}_{3, 1}+A^{(i, j)}_{3, 3}+A^{(i, j)}_{2, 2}+A^{(i, j)}_{2, 4}+A^{(i, j)}_{4, 2}+A^{(i, j)}_{4, 4}\\ -&\big ( A^{(i, j)}_{1, 2}+A^{(i, j)}_{2, 1}+A^{(i, j)}_{1, 4}+A^{(i, j)}_{4, 1}+A^{(i, j)}_{2, 3}+A^{(i, j)}_{3, 2}+A^{(i, j)}_{3, 4}+A^{(i, j)}_{4, 3} \big )\Big ], \\ \end{aligned} \end{aligned}$$

with $$A^{(i, j)}_{n, m}:=A_{4(i-1)+n, 4(j-1)+m}$$, $$n, m\in \{ 1, 2, 3, 4 \}$$, $$i, j\in \{ 1, \dots , N \}$$ and

4.24 $$\begin{aligned} c_j:= \frac{2}{\varepsilon }(\mathtt {C}_j-1). \end{aligned}$$

Recall that *A* is symmetric, hence $d_{i j}=d_{j i}$.

#### Remark 4.4

We point out that the variables $K_i$ in (4.22) reads, in the coordinates $\{ \alpha _j \}_j$ [see (4.9)] as

$$\begin{aligned} K_i:=|\alpha _{n_{4 (i-1)+1}}|^2=|\alpha _{n_{4 (i-1)+3}}|^2=1-|\alpha _{n_{4 (i-1)+2}}|^2=1-|\alpha _{n_{4 (i-1)+4}}|^2 \quad \forall i=1, \dots , N. \end{aligned}$$

It can be easily seen that the hyperplanes $\{K_j=0\}$, $\{K_j=1\}$ are invariant under the Hamiltonian (4.22). Indeed one can understand the Hamiltonian (4.22) as defined on the product sphere $(S^2)^N$ by “blowing down” the sets $\{K_j=0\}$, $\{K_j=1\}$ to a point in each sphere. That is, one can consider local coordinates

4.25 $$\begin{aligned} x_j=\sqrt{2K_j}\cos \frac{\psi _j}{2}, \quad y_j=\sqrt{2K_j}\sin \frac{\psi _j}{2} \end{aligned}$$

which blow down $\{K_j=0\}$. Then, the Hamiltonian (4.22) becomes

4.26 $$\begin{aligned} \begin{aligned} {\mathcal {H}}(x_1, \ldots , x_N, y_1, \ldots , y_N)&= \frac{1}{2}\sum _{j=1}^N \left( 3x_j^2-y_j^2\right) -\frac{1}{4}\sum _{j=1}^N \left( 3x_j^2-y_j^2\right) \left( x_j^2+y_j^2\right) \\&\quad +\varepsilon \Bigg [ \frac{1}{2}\sum _{j=1}^N a_{j} \left( x_j^2+y_j^2\right) +\frac{1}{4}\sum _{j=1}^N b_{j} \left( x_j^2+y_j^2\right) ^2 \\ {}&\quad + \frac{1}{4}\sum _{i, j=1, i< j}^N d_{i j} \left( x_i^2+y_i^2\right) \left( x_j^2+y_j^2\right) \\ {}&\quad +\frac{1}{4}\sum _{h=1}^N c_j \left( x_j^2-y_j^2\right) \left( 2-x_j^2-y_j^2\right) \Bigg ]. \end{aligned} \end{aligned}$$

From the particular form of this Hamiltonian, it is clear that $\{x_j=y_j=0\}$ is invariant under the associated flow. In particular the point

4.27 $$\begin{aligned} P_-=\left\{ x_j=0, y_j=0,\quad j=1\ldots N\right\} , \end{aligned}$$

is a saddle (for small $\varepsilon$) with *N* dimensional stable and unstable manifolds. One can analogously blow down $\{K_j=1\}$ by considering the coordinates

$$\begin{aligned} x_j=\sqrt{2(1-K_j)}\cos \frac{\psi _j}{2}, \quad y_j=\sqrt{2(1-K_j)}\sin \frac{\psi _j}{2} \end{aligned}$$

and one also obtains that, for $\varepsilon$ small enough, $P_+=\{x_j=0, y_j=0,\,\, j=1\ldots N\}$ is a saddle with *N* dimensional stable and unstable manifolds. This saddle is the “blow down” of $\{K_1=\ldots =K_N=1\}$.

## Dynamics of the Resonant Model

The reduced Hamiltonian (4.22) for $N=2$ is of the form

5.1 $$\begin{aligned} \begin{aligned} {\mathcal {H}}(\varepsilon ; \psi _1, \psi _2, K_1, K_2)=&{\mathcal {H}}_0(\psi _1, \psi _2, K_1, K_2)+\varepsilon {\mathcal {H}}_1(\psi _1, \psi _2, K_1, K_2)\\ {\mathcal {H}}_0(\psi _1, \psi _2, K_1, K_2)=&\,{\mathcal {H}}_0^{(1)}(\psi _1, K_1)+{\mathcal {H}}_0^{(2)} (\psi _2, K_2)\\ {\mathcal {H}}_0^{(1)}(\psi _1, K_1)=\,&\,K_1(1-K_1)(1+2\cos (\psi _1))\\ {\mathcal {H}}_0^{(2)}(\psi _2, K_2)=\,&\,K_2(1-K_2)(1+2\cos (\psi _2))\\ {\mathcal {H}}_1(\psi _1, \psi _2, K_1, K_2)=\,&\,a_1K_1+b_1K_1^2+a_2 K_2+b_2 K_2^2 +c_1K_1(1-K_1)\cos (\psi _1)\\&+c_2K_2(1-K_2)\cos (\psi _2) +d_{12}K_1K_2. \end{aligned} \end{aligned}$$

Note that the only term which couples the two unperturbed Hamiltonians ${\mathcal {H}}_0^{(1)}$, ${\mathcal {H}}_0^{(2)}$ is $d_{12}K_1K_2$. The Hamiltonian ${\mathcal {H}}$ is reversible with respect to the involution

5.2 $$\begin{aligned} \Upsilon (\psi _1, \psi _2, K_1, K_2)=(-\psi _1,-\psi _2, K_1, K_2). \end{aligned}$$

### Unperturbed dynamics ($\varepsilon =0$)

For $\varepsilon =0$, the Hamiltonian system ${\mathcal {H}}_0$ is the product of the two uncoupled 1-d.o.f systems with Hamiltonian ${\mathcal {H}}_0^{(i)}$, $i=1, 2$ and therefore it is integrable. We analyze the dynamics given by ${\mathcal {H}}_0^{(i)}$. We analyze it only for ${\mathcal {H}}_0^{(1)}$ since both Hamiltonians are equal.

The associated equations of motion are given by

$$\begin{aligned} \begin{aligned} {\dot{\Psi }}_1=&\,(1-2 K_1) (1+2\cos (\psi _1))\\ {\dot{K}}_1=&\,2\sin (\psi _1)\,K_1\,(1-K_1). \end{aligned} \end{aligned}$$

The sets $\{ K_1=0\}$ and $\{K_1=1\}$ are ${\mathcal {H}}_0^{(1)}$-invariant 1-dimensional tori which correspond to the hyperbolic tori (4.15) after symplectic reduction and correspond to saddles in proper “blow down” coordinates [see (4.25), (4.27)). The sets $\{ K_1=0\}$ and $\{K_1=1\}$ possess the hyperbolic equilibrium points $\left( \pm \Psi _{*}, 0\right)$, $\left( \pm \Psi _{*}, 1 \right)$ with

5.3 $$\begin{aligned} \Psi _*=2\pi /3. \end{aligned}$$

Such equilibria are hyperbolic with eigenvalues $\pm \sqrt{3}$. Their invariant manifolds outside of $\{ K_1=0\}$ and $\{K_1=1\}$ correspond to the invariant manifolds of the saddles $P_\pm$ in (4.27).

The tori $\{ K_1=0\}$ and $\{K_1=1\}$ are on the same energy level ${\mathcal {H}}_0^{(1)}=0$ and the saddles $\left( \pm \Psi _{*}, 0\right)$ and $\left( \pm \Psi _{*}, 1 \right)$ are connected through the heteroclinic orbits

$$\begin{aligned} (\psi _1(t), K_1(t))=\left( \pm \Psi _*, \dfrac{1}{1+e^{\mp \sqrt{3} t}}\right) \end{aligned}$$

(see Fig. 4).

**Fig. 4:**
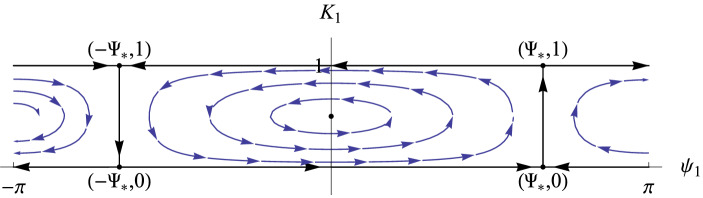
Phase space of the Hamiltonian ${\mathcal {H}}_0^{(1)}$ in (5.1)

For $0<K_1<1$, the dynamics of the Hamiltonian ${\mathcal {H}}_0^{(1)}$ can be also analyzed easily. Consider the “half” of the phase space $(-2\pi /3,2\pi /3)\times (0, 1)\subset {\mathbb {T}}\times (0, 1)$ limited by the heteroclinic orbits (the other “half” is symmetric). It has an elliptic points at $(\psi _1,K_1)=(0,1/2)$ and the rest is foliated by periodic orbits

5.4 $$\begin{aligned} \mathtt {P}_h:=\{{\mathcal {H}}_0^{(1)}=h\} \qquad \text{ with } \qquad h\in (0,3/4). \end{aligned}$$

When $h\rightarrow 0$, the periodic orbits “tend” to the sequence of heteroclinics and $K_1=0,1$ and therefore their period $\mathtt {T}_h\rightarrow +\infty$.

Hence, the dynamics of the 2-dof Hamiltonian ${\mathcal {H}}_0$ in (5.1) has the following features. The invariant tori

5.5 $$\begin{aligned} {\mathbb {T}}_0:=\{ K_1=K_2=0\}, \qquad {\mathbb {T}}_1:=\{ K_1=K_2=1 \} \end{aligned}$$

are two invariant Lagrangian tori for the system (5.1). They possess the equilibrium points

5.6 $$\begin{aligned}&{\mathfrak {e}}_+^{(0)}:=(\Psi _{*}, \Psi _{*} , 0, 0), \quad {\mathfrak {e}}_+^{(1)}:=(\Psi _{*}, \Psi _{*} , 1, 1),\nonumber \\&\quad {\mathfrak {e}}_-^{(1)}:=(-\Psi _{*}, -\Psi _{*} , 1, 1), \quad {\mathfrak {e}}_-^{(0)}:=(-\Psi _{*}, -\Psi _{*} , 0, 0) \end{aligned}$$

connected by the following heteroclinic manifolds

5.7 $$\begin{aligned} \begin{aligned} \gamma _{+}(\tau _1, \tau _2):=(\psi _1^+(\tau _1), \psi _2^+(\tau _2), K_1^+(\tau _1), K_2^+(\tau _2))&=\left( \Psi _{*}, \Psi _{*}, \dfrac{1}{1+e^{-\sqrt{3}\tau _1}}, \dfrac{1}{1+e^{-\sqrt{3} \tau _2}} \right) ,\\ \gamma _{-}(\tau _1, \tau _2):=(\psi _1^-(\tau _1), \psi _2^-(\tau _2), K_1^-(\tau _1), K_2^-(\tau _2))&=\left( -\Psi _*, -\Psi _*, \dfrac{1}{1+e^{\sqrt{3}\tau _1}}, \dfrac{1}{1+e^{\sqrt{3}\tau _2}} \right) . \end{aligned} \end{aligned}$$

In particular $\gamma _+$ connects the points ${\mathfrak {e}}_+^{(0)}$, ${\mathfrak {e}}_+^{(1)}$ and $\gamma _-$ connects ${\mathfrak {e}}_-^{(1)}$ with ${\mathfrak {e}}_-^{(0)}$. The trajectories in the heteroclinic manifolds are just given by $\gamma _{\pm }(\tau _1+t, \tau _2+t)$, $t\in {\mathbb {R}}$.

The 4-dimensional phase space of Hamiltonian ${\mathcal {H}}$ in (5.1) with $\varepsilon =0$ has several three-dimensional invariant subspaces setting either $K_1$ or $K_2$ equal to 0 or 1, and two dimensional invariant subspaces setting either $(\psi _1,K_1)$ or $(\psi _2,K_2)$ at one of the saddles. Thus, one can define the hyperbolic periodic orbits (recall (5.4)]

5.8 $$\begin{aligned} \mathtt {P}^{\sigma ,s}_h := \{ (\psi _1,\psi _2,K_1,K_2) : (\psi _1,K_1) \in \mathtt {P}_h, \,\psi _2= \sigma \Psi _*,\, K_2=k \}, \; \; \sigma = \pm , \; \; s =0,1, \end{aligned}$$

and one could define analogously the other ones placing them at the other saddles.

For $\varepsilon =0$, the Hamiltonian system (5.1) possesses two 2-dimensional heteroclinic manifolds $W^{s}({\mathfrak {e}}_{+}^{(0)})=W^u({\mathfrak {e}}_{+}^{(1)})$, $W^{u}({\mathfrak {e}}_{-}^{(0)})=W^s({\mathfrak {e}}_{-}^{(1)})$. They are certainly not robust under perturbations. We show that, under a generic non-degeneracy condition, those heteroclinic manifolds break down when $0<\varepsilon \ll 1$ creating transverse intersections between some of the stable and unstable invariant manifolds.

### Non-integrable dynamics $(\varepsilon >0)$: 2 resonant tuples

For $\varepsilon >0$, the tori ${\mathbb {T}}_0$ and ${\mathbb {T}}_1$ in (5.5) are still invariant and they still possess saddles which are $\varepsilon$–close to the unperturbed saddles ${\mathfrak {e}}_\pm ^{(j)}$, $j=0,1$. These saddles have 2-dimensional stable and unstable invariant manifolds.

#### Remark 5.1

Abusing notation, we also denote by ${\mathfrak {e}}_\pm ^{(j)}$, $j=0,1$ the saddles of the perturbed Hamiltonian (5.1) with $0<\varepsilon \ll 1$, which are $\varepsilon$-close to those defined by (5.6).

#### Theorem 5.2

Consider the Hamiltonian (5.1) and assume that

5.9 $$\begin{aligned} d_{12}\ne 0\qquad \text {(see}\, \text {(}5.1\text {))}. \end{aligned}$$

Then, there exists $\varepsilon _0>0$ such that for all $\varepsilon \in (0, \varepsilon _0)$, the invariant manifolds $W_{\varepsilon }^{u}({\mathfrak {e}}_{+}^{(0)})$ and $W_{\varepsilon }^s({\mathfrak {e}}_{-}^{(0)})$ of the saddles (5.6) of the Hamiltonian (5.1) intersect transversally along an orbit (within the energy level).

Note that this theorem is not a classical perturbative result. Indeed, for $\varepsilon =0$ the saddles ${\mathfrak {e}}_{\pm }^{(0)}$ did not have any connection since their invariant manifolds coincided with those of ${\mathfrak {e}}_{\pm }^{(1)}$ along heteroclinic connections. Therefore, the prove of Theorem 5.2 is not a direct consequence of Melnikov Theory (is not a theorem about persistence, is a theorem about *new* heteroclinic connections). Thus, we prove this theorem in two steps. First in Sect. 5.2.1 we apply Melnikov Theory to prove the existence of transverse (within the energy level) heteroclinic connections between ${\mathfrak {e}}_{+}^{(0)}$ and ${\mathfrak {e}}_{+}^{(1)}$ (under certain conditions). Then, in Sect. 5.2.2, we use this analysis to prove the existence of the connections given in Theorem 5.2 through a suitable modification of Melnikov Theory.

#### Transversal heteroclinic orbits to saddles

The first step to prove Theorem 5.2 is to prove the existence of heteroclinic intersections between the saddles ${\mathfrak {e}}_{\pm }^{(0)}$ and ${\mathfrak {e}}_{\pm }^{(1)}$. This step is certainly not necessary to obtain homoclinic intersections. Nevertheless, it will make considerably easier the computation of the Melnikov function associated to the homoclinic intersections. To obtain the mentioned heteroclinic intersections, one certainly needs that the saddles belong to the same energy level, that is, ${\mathcal {H}}({\mathfrak {e}}_{\pm }^{(0)})={\mathcal {H}}({\mathfrak {e}}_{\pm }^{(1)})$. By (5.1) this condition is equivalent to

5.10 $$\begin{aligned} a_1+b_1+a_2+b_2+d_{12}=0. \end{aligned}$$

##### Proposition 5.3

The Hamiltonian (5.1) possesses four hyperbolic fixed points ${\mathfrak {e}}_{\pm }^{(0)}$, ${\mathfrak {e}}_{\pm }^{(1)}$ such that the following holds. If (5.10) is satisfied and

5.11 $$\begin{aligned} (a_1+b_1)(a_2+b_2)>0, \end{aligned}$$

there exists $\varepsilon _0>0$ such that for $\varepsilon \in (0, \varepsilon _0)$ the manifolds $W_{\varepsilon }^{u}({\mathfrak {e}}_+^{(0)})$ and $W_{\varepsilon }^{s}({\mathfrak {e}}_+^{(1)})$ intersect transversally along orbits (within the energy level). The same happens for $W_{\varepsilon }^{s}({\mathfrak {e}}_-^{(0)})$ and $W_{\varepsilon }^{u}({\mathfrak {e}}_-^{(1)})$.

**Fig. 5:**
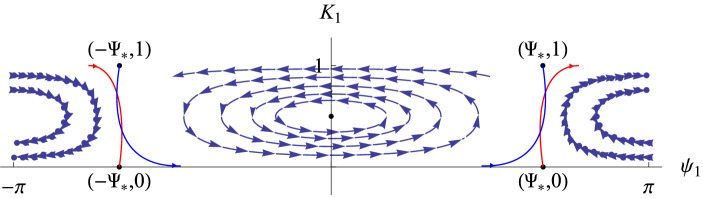
Transverse heteroclinic orbits for $\varepsilon$ small enough

See in Fig. 5 an example of heteroclinic connections. We devote the rest of the section to prove Proposition 5.3.

##### Proof of Proposition 5.3

Thanks to the symmetry (5.2) of the system (5.1), one of the intersections implies the other one. We just deal with the first one.

Consider a compact subset ${\mathbf {K}}$ of ${\mathbb {R}}^2$. Let $\mathbf {\tau }=(\tau _1, \tau _2)\in {\mathbf {K}}$ and $m>0$. We consider the line

$$\begin{aligned} \Sigma (\mathbf {\tau })=\{\gamma _+(\mathbf {\tau })+r\,\nabla {\mathcal {H}}^{(1)}_0 (\gamma _+(\mathbf {\tau })),\,\,\,r\in (-m, m)\}, \end{aligned}$$

which passes through $\gamma _+(\mathbf {\tau })$ and it is orthogonal to $\{{\mathcal {H}}^{(1)}_0={\mathcal {H}}^{(1)}_0(\gamma _+(\mathbf {\tau }))\}$ at $\gamma _+(\mathbf {\tau })$. Since the system has two degrees of freedom and energy conservation, it is enough to measure the distance along this line. It would be equivalent to consider ${\mathcal {H}}^{(2)}_0$. Since $\gamma _+(\mathbf {\tau })\in W_{0}^s({\mathfrak {e}}_+^{(1)})=W_{0}^u({\mathfrak {e}}_+^{(0)})$, if we consider $\varepsilon$ small enough we can ensure that $\Sigma (\mathbf {\tau })$ intersects transversally $W_{\varepsilon }^s({\mathfrak {e}}_+^{(1)})$ and $W_{\varepsilon }^u({\mathfrak {e}}_+^{(0)})$ at just one point, $q_{\varepsilon }^s=q_{\varepsilon }^s(\mathbf {\tau })$ and $q_{\varepsilon }^u=q_{\varepsilon }^u(\mathbf {\tau })$ respectively. Then, the distance between the invariant manifolds in $\Sigma (\mathbf {\tau })$ is given by

5.12 $$\begin{aligned} d(\mathbf {\tau }):=\left\langle \dfrac{ \nabla {\mathcal {H}}^{(1)}_0 (\gamma _+(\mathbf {\tau }))}{\Vert \nabla {\mathcal {H}}^{(1)}_0 (\gamma _+(\mathbf {\tau })) \Vert }, q_{\varepsilon }^s(\mathbf {\tau })-q_{\varepsilon }^u(\mathbf {\tau }) \right\rangle . \end{aligned}$$

Application of the classical Melnikov Theory gives the following result.

##### Lemma 5.4

The function $d(\mathbf {\tau })$ introduced in (5.12) satisfies

$$\begin{aligned} d(\mathbf {\tau })=\dfrac{\varepsilon }{\Vert \nabla {\mathcal {H}}^{(1)}_0 (\gamma _+(\mathbf {\tau })) \Vert } \,\mathcal {M_+}(\mathbf {\tau })+{\mathcal {O}}_{C^1({\mathbf {K}})}(\varepsilon ^2), \qquad \mathbf {\tau }\in {\mathbf {K}}, \end{aligned}$$

where

5.13 $$\begin{aligned} \begin{aligned}&\mathcal {M_+}(\mathbf {\tau }):= \int _{{\mathbb {R}}} \{ {\mathcal {H}}^{(1)}_0 , {\mathcal {H}}_1\} \circ \Phi ^t_{{\mathcal {H}}_0}(\gamma _{+}(\mathbf {\tau }))\,dt = \int _{{\mathbb {R}}} \{ {\mathcal {H}}^{(1)}_0 , {\mathcal {H}}_1\} \circ (\gamma _{+}(\tau _1+t,\tau _2+t))\,dt \end{aligned} \end{aligned}$$

is the so-called *Melnikov function* (see [36]).

Since the Hamiltonian system (5.1) is autonomous, the Melnikov function $\mathcal {M_+}$ depends just on the one-dimensional variable $\tau _1-\tau _2$. That is, there exists a function ${{\mathcal {M}}}_+^{(0)}:{\mathbb {R}}\rightarrow {\mathbb {R}}$ such that

$$\begin{aligned} {{\mathcal {M}}}_+(\tau _1,\tau _2)={{\mathcal {M}}}^{(0)}_+(\tau _1-\tau _2). \end{aligned}$$

By Lemma 5.4, we will deduce Theorem 5.3 by proving that there exists a non-degenerate zero of the function ${\mathcal {M}}^{(0)}_+$ in (5.13).

It is convenient to introduce the Melnikov potential ${\mathcal {L}}_{+}:{\mathbb {R}}^2\rightarrow {\mathbb {R}}$, since it is usually easier to compute. It is defined, up to constants, as a primitive of the Melnikov function, namely

$$\begin{aligned} \partial _{\tau _1} {\mathcal {L}}_+ (\mathbf {\tau })={\mathcal {M}}_+ (\mathbf {\tau }). \end{aligned}$$

We have

$$\begin{aligned} {\mathcal {L}}_+(\mathbf {\tau })=\int _{{\mathbb {R}}}{\mathcal {H}}_1\circ \Phi ^t_{{\mathcal {H}}_0}\left( \gamma _+(\mathbf {\tau })\right) \,dt=\int _{{\mathbb {R}}}{\mathcal {H}}_1\left( \gamma _+(\tau _1+t,\tau _2+t)\right) \,dt. \end{aligned}$$

Recall that we are assuming (5.10), which implies ${\mathcal {H}}_1({\mathfrak {e}}_+^{(0)})={\mathcal {H}}_1({\mathfrak {e}}_+^{(1)})=0$. Therefore, the integrand decays exponentially to zero as $t\rightarrow \pm \infty$.

The Melnikov potential satisfies ${\mathcal {L}}_+(\mathbf {\tau })= {\mathcal {L}}^{(0)}_+(\tau _0)$ where $\tau _0:=\tau _1-\tau _2$ and ${\mathcal {L}}^{(0)}_+$ is called *reduced Melnikov potential*. Then,

$$\begin{aligned} \partial _{\tau _0} {\mathcal {L}}_{+}^{(0)}(\tau _0)={\mathcal {M}}^{(0)}_+(\tau _0). \end{aligned}$$

Hence we shall look for non-degenerate critical points of ${\mathcal {L}}_{+}^{(0)}$, which correspond to non-degenerate zeros of ${\mathcal {M}}_{+}^{(0)}$. The following lemma concludes the proof of Proposition 5.3.

##### Lemma 5.5

There exists a constant ${\tilde{\eta }}\in {\mathbb {R}}$ such that the reduced Melnikov potential ${\mathcal {L}}^{(0)}_+$ is given by

5.14 $$\begin{aligned} {\mathcal {L}}^{(0)}_+(\tau _0) =\tau _0\,\,\dfrac{(a_1+b_1)\,e^{-\sqrt{3}\tau _0}+(a_2+b_2)}{ 1-e^{-\sqrt{3} \tau _0} }+{\tilde{\eta }}. \end{aligned}$$

Therefore, provided (5.11) is satisfied, it possesses a non-degenerate critical point.

##### Remark 5.6

Note that $\Upsilon \gamma _+(\mathbf {\tau })=\gamma _-(-\mathbf {\tau })$, $i=1, 2$ [see (5.7)] where $\Upsilon$ is the involution introduced in (5.2).Then ${\mathcal {L}}_+(\mathbf {\tau })={\mathcal {L}}_-(-\mathbf {\tau })$ and ${\mathcal {L}}^{(0)}_+(\tau _0)={\mathcal {L}}^{(0)}_-(-\tau _0)$ . Therefore, if (5.11) holds, ${\mathcal {L}}_-^{(0)}$ has a non-degenerate critical point.

##### Proof of Lemma 5.5

Using the definition of ${\mathcal {H}}_0$ in (5.1), (5.10), one can write ${\mathcal {L}}_+$ as

$$\begin{aligned} \begin{aligned} {\mathcal {L}}_+(\mathbf {\tau })&= (a_2+b_2)\int _{{\mathbb {R}}} K_1^+(t+\tau _1)(1-K_2^+(t+\tau _2))\,dt +(a_1+b_1)\int _{{\mathbb {R}}} K_2^+(t+\tau _2)(1-K_1^+(t+\tau _1))\,dt+{\tilde{\eta }}\\&=(a_2+b_2)\int _{{\mathbb {R}}} K_1^+(s+\tau _0)(1-K_2^+(s))\,ds +(a_1+b_1)\,\int _{{\mathbb {R}}} K_2^+(s)(1-K_1^+(s+\tau _0))\,ds+{\tilde{\eta }}\\&=:{\mathcal {L}}_+^{(0)}(\tau _0), \end{aligned} \end{aligned}$$

where $\tau _0=\tau _1-\tau _2$ and the constant ${\tilde{\eta }}\in {\mathbb {R}}$ is given by

$$\begin{aligned} \begin{aligned} {\tilde{\eta }}:=&\,\int _{\mathbb {R}}\left( b_1 K_1(t)(K_1(t)-1)+b_2 K_2(t)(K_2(t)-1)\right) \,dt\\&+\,\int _{\mathbb {R}}\left( c_1 K_1(t)(1-K_1(t))\cos \Psi _*+c_2 K_2(t)(1-K_2(t))\cos \Psi _* \right) \,dt. \end{aligned} \end{aligned}$$

For $i, j=1, 2$ we have [recall (5.7)]

5.15 $$\begin{aligned} \begin{aligned} \int _{{\mathbb {R}}} K^+_i(t+\tau _i) (1-K^+_j(t+\tau _j))\,dt&= \int _{{\mathbb {R}}} \dfrac{e^{-\sqrt{3}(t+\tau _j)}}{(1+e^{-\sqrt{3}(t+\tau _i)})(1+e^{-\sqrt{3}(t+\tau _j)}) } \,dt\\&=(\tau _i-\tau _j) \dfrac{1}{1-e^{-\sqrt{3}(\tau _i-\tau _j)}}, \end{aligned} \end{aligned}$$

which gives (5.14). Therefore, we have that

$$\begin{aligned} \lim _{\tau _0\rightarrow +\infty } \partial _{\tau _0} {\mathcal {L}}^{(0)}_+(\tau _0)=a_2+b_2, \qquad \lim _{\tau _0\rightarrow -\infty } \partial _{\tau _0}{\mathcal {L}}^{(0)}_+(\tau _0)=-(a_1+b_1) . \end{aligned}$$

If $(a_1+b_1)(a_2+ b_2)>0$ (see (5.11)) the reduced Melnikov potential ${\mathcal {L}}^{(0)}_{+}$ has at least one critical point. Moreover,

$$\begin{aligned} \,\partial _{\tau _0}^2{\mathcal {L}}^{(0)}_+(\tau _0)=-(a_1+b_1+a_2+b_2) \dfrac{\sqrt{3}}{4}\left( 2-\sqrt{3} \tau _0\coth \left( \dfrac{\sqrt{3}\tau _0}{2} \right) \right) {\mathrm {csch}^2\left( \dfrac{\sqrt{3} \tau _0}{2} \right) } . \end{aligned}$$

By (5.11) this function has constant sign since

$$\begin{aligned} 2-\sqrt{3}\tau _0\,\coth \left( \dfrac{\sqrt{3}\tau _0}{2} \right) < 0 \qquad \forall \tau _0\ne 0,\\ \lim _{\tau _0\rightarrow 0} \left( 2-\sqrt{3}\tau _0\coth \left( \dfrac{\sqrt{3}\tau _0}{2} \right) \right) { \mathrm {csch}^2\left( \dfrac{\sqrt{3} \tau _0}{2} \right) } =-\frac{2}{3}. \end{aligned}$$

Therefore ${\mathcal {L}}_{+}^{(0)}$ is either convex or concave (depending on the sign of $a_1+b_1+a_2+b_2$) and its critical points are non-degenerate. $\quad \square$

#### Transversal homoclinic orbits to saddles: Proof of Theorem 5.2

We use the computation of the heteroclinic Melnikov potential in Lemma 5.5 to prove the existence of homoclinic transversal intersections given by Theorem 5.2.

Since the Hamiltonian (5.1) with $\varepsilon =0$ does not have connections between ${\mathfrak {e}}_{\pm }^{(0)}$, we cannot apply directly Melnikov Theory to obtain such connections for $\varepsilon >0$. Instead, we exploit the usual technique of considering a modified unperturbed Hamiltonian and using two parameters $\varepsilon$ and $\delta$.

We consider the Hamiltonian

5.16 $$\begin{aligned} \begin{aligned} {\mathcal {H}} ={\mathbf {H}}_0+\varepsilon {\mathbf {H}}_1, \quad {\mathbf {H}}_0(\psi _1, \psi _2, K_1, K_2)&={\mathbf {H}}_0^{(1)}+{\mathbf {H}}_0^{(2)},\\ {\mathbf {H}}_0^{(1)}(\psi _1, \psi _2, K_1, K_2)&=K_1(1-K_1)(1+2\, \cos (\psi _1))-\delta K_1^2,\\ {\mathbf {H}}_0^{(2)}(\psi _1, \psi _2, K_1, K_2)&=K_2(1-K_2)(1+2\, \cos (\psi _2))-\delta K_2^2 \end{aligned} \end{aligned}$$

and

5.17 $$\begin{aligned} \begin{aligned} {\mathbf {H}}_1(\psi _1, \psi _2, K_1, K_2):=&\,d_{12}\, K_1 \,K_2 + a_1 K_1+(b_1+1) K_1^2+c_1\,K_1(1-K_1) \cos (\psi _1) \\&\quad +a_2 K_2+(b_2+1) K_2^2+c_2\,K_2(1-K_2) \cos (\psi _2) . \end{aligned} \end{aligned}$$

If one takes $\delta =\varepsilon$, this Hamiltonian coincides with (5.1). Nevertheless, for now we consider $\delta$ and $\varepsilon$ independent parameters. Later one we will take $\delta =\varepsilon$.

If $\delta =0$, then the dynamics of ${\mathbf {H}}_0$ is the same described in Sect. 5.1. If $\delta \ne 0$, the tori defined in (5.5) are ${\mathbf {H}}_0$-invariant; moreover, they belong to different energy levels, since

$$\begin{aligned} {\mathbf {H}}_{0_{| {\mathbb {T}}_0 }}=0, \qquad {\mathbf {H}}_{0_{| {\mathbb {T}}_1 }}=-2\delta . \end{aligned}$$

**Fig. 6:**
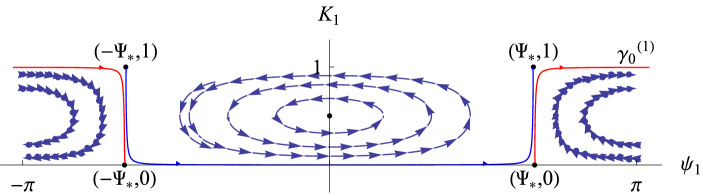
Phase space restricted to the $(\psi _1,K_1)$-coordinates for the Hamiltonian ${\mathcal {H}}$ in (5.16)–(5.17)

The equilibrium points contained in ${\mathbb {T}}_0$ are the saddles ${\mathfrak {e}}_{\pm }^{(0)}$ defined in (5.6). Now we compute the heteroclinic manifold that connects (forward in time) ${\mathfrak {e}}^{(0)}_+$ with ${\mathfrak {e}}^{(0)}_-$ (see Fig. 6). Such orbit corresponds to a homoclinic to the saddle $P_-$ in (4.27) (expressed in the “blow down” coordinates (4.25)).

##### Lemma 5.7

The saddles ${\mathfrak {e}}^{(0)}_+$ with ${\mathfrak {e}}^{(0)}_-$ of Hamiltonian ${\mathbf {H}}_0$ in (5.16) are connected by a two-dimensional heteroclinic manifold parameterized as

5.18 $$\begin{aligned} \begin{aligned} \gamma _0(\mathbf {\tau }):&=(\gamma _0^{(1)}(\tau _1), \gamma _0^{(2)}(\tau _2))=(\psi _1^{(0)}(\tau _1), \psi _2^{(0)}(\tau _2), K_1^{(0)}(\tau _1), K_2^{(0)}(\tau _2)),\\ \psi _j^{(0)}(\tau _j):&=2\,\arctan (\Lambda (\tau _j)), \quad K_j^{(0)}(\tau _j)=\frac{1}{1-\frac{\delta }{3}(1-2\cosh (\sqrt{3}\tau _j))}\quad j=1, 2, \end{aligned} \end{aligned}$$

where $$\Lambda (t):=-\sqrt{3}\,\, \tanh \left( \dfrac{\sqrt{3}}{2} t \right) $$.

##### Proof

Using that ${\mathbf {H}}_0^{(1)}$ is zero when restricted to ${\mathbb {T}}_0$ we get

5.19 $$\begin{aligned} K_1=\frac{1+2 \cos (\psi _1)}{1+2 \cos (\psi _1)+\delta }. \end{aligned}$$

When the angle $\psi _1\in [-\Psi _*, \Psi _*]$ the numerator in (5.19) is positive. Hence $K_1\in (0, 1)$ if $\delta >0$. Plugging (5.19) in the equation for $\psi _1$ we have

$$\begin{aligned} {\dot{\psi }}_1=-(1+2\cos (\psi _1)), \end{aligned}$$

which leads to

5.20 $$\begin{aligned} \psi _1(t)=2\,\arctan (\Lambda (t)), \quad \Lambda (t)=-\sqrt{3}\,\, \tanh \left( \dfrac{\sqrt{3}}{2} t \right) . \end{aligned}$$

By using (5.19) and the trigonometric identity $\cos (2 \arctan (x))=(1-x^2)/(1+x^2)$ we have

5.21 $$\begin{aligned} K_j^{(0)}(t)=\frac{1}{1-\frac{\delta }{3}(1-2\cosh (\sqrt{3}t))}. \end{aligned}$$

Reasoning in the same way for $(\psi _2, K_2)$ we get that the homoclinic orbit to ${\mathbb {T}}_0$ is given by (5.18). $\quad \square$

By reasoning as in the proof of Theorem 5.3 we have that the distance between the manifolds in a suitable section is given by

5.22 $$\begin{aligned} d(\mathbf {\tau })=\dfrac{\varepsilon }{\Vert \nabla {\mathbf {H}}^{(1)}_0 (\gamma _0(\mathbf {\tau })) \Vert } \,{\mathcal {M}}_0(\mathbf {\tau })+{\mathcal {O}}_{C^1({\mathbf {K}})}(\varepsilon ^2), \qquad \mathbf {\tau }\in {\mathbf {K}}, \end{aligned}$$

where the Melnikov function is given by

5.23 $$\begin{aligned} {\mathcal {M}}_0(\mathbf {\tau })=\int _{{\mathbb {R}}} \{ {\mathbf {H}}^{(1)}_0, {\mathbf {H}}_1\}\circ \Phi ^t_{{\mathbf {H}}_0}(\gamma _0(\mathbf {\tau }))\,dt. \end{aligned}$$

It can be easily checked that the ${\mathcal {O}}_{C^1({\mathbf {K}})}(\varepsilon ^2)$ are uniform for $\delta$ small enough.

The associated Melnikov potential is

$$\begin{aligned} {\mathcal {L}}_0(\mathbf {\tau })&=\int _{{\mathbb {R}}} {\mathbf {H}}_1\circ \Phi ^t_{{\mathbf {H}}_0}(\gamma _0(\mathbf {\tau }))\,dt ={d_{12}} \int _{{\mathbb {R}}} K^{(0)}_1(t+\tau _1) \,K^{(0)}_2(t+\tau _2)\,dt+\eta _* \end{aligned}$$

where

$$\begin{aligned} \begin{aligned} \eta _*=&\int _{{\mathbb {R}}} \Big (a_1 K^{(0)}_1(t)+(b_1+1) (K^{(0)}_1(t))^2+c_1\,K^{(0)}_1(t)(1-K^{(0)}_1(t)) \cos \psi ^{(0)}_1(t)\Big )\,dt\\&+\int _{{\mathbb {R}}} \Big (a_2 K^{(0)}_2(t)+(b_2+1) (K^{(0)}_2(t))^2+c_2\,K^{(0)}_2(t)(1-K^{(0)}_2(t)) \cos \psi ^{(0)}_2(t) \Big )\,dt. \end{aligned} \end{aligned}$$

As before we consider the reduced Melnikov potential

5.24 $$\begin{aligned} {\mathcal {L}}_0^{(0)}(\tau _0)={d_{12}} \int _{{\mathbb {R}}} K^{(0)}_1(s+\tau _0) \,K^{(0)}_2(s)\,ds+\eta _*. \end{aligned}$$

We want to deduce that ${\mathcal {L}}_0^{(0)}$ has non-degenerate critical points by using the information on the Melnikov potentials (5.14) of the heteroclinic case.

##### Proposition 5.8

Fix an interval ${\mathcal {I}}\subset {\mathbb {R}}$. There exists $\delta _0>0$ such that $\forall \delta \in (0, \delta _0)$ there exists a real number $\eta$ and a constant $\nu _0>0$ such that, for $\tau _0\in {\mathcal {I}}$,

$$\begin{aligned} {\mathcal {L}}^{(0)}_0(\tau _0)=\eta +d_{1 2}\,\tau _0\,\coth \left( \frac{\sqrt{3}\tau _0}{2} \right) +{\mathcal {O}}_{C^2({\mathcal {I}})}(\delta ^{\nu _0}). \end{aligned}$$

The proof of this proposition is deferred to Sect. 5.2.3. To complete the proof of Theorem 5.2 it is enough use (5.22) and Proposition 5.8 and take $\delta =\varepsilon$. Indeed, the transverse homoclinic points are $\varepsilon$-close to the non-degenerate critical point for ${\mathcal {L}}_0^{(0)}$. By Proposition 5.8, ${\mathcal {L}}_0^{(0)}$ has a non-degenerate critical point $\varepsilon ^{\nu _0}$-close to $\tau _0=0$.

#### Proof of Proposition 5.8

Thanks to the exponential convergence of the homoclinic orbit to the equilibrium points ${\mathfrak {e}}_\pm ^{(0)}$ we have that (recall (5.18))

$$\begin{aligned} \int _{{\mathbb {R}}} K_j^{(0)}(t)\,dt<\infty \qquad i=1, 2. \end{aligned}$$

We write ${\mathcal {L}}^{(0)}_0$ as

5.25 $$\begin{aligned} \begin{aligned} {\mathcal {L}}^{(0)}_0(\tau _0)=-d_{12}\int _{{\mathbb {R}}} K^{(0)}_1(s+\tau _0) \,(1-K^{(0)}_2(s))\,ds+\eta _1, \end{aligned} \end{aligned}$$

where

$$\begin{aligned} \eta _1=\eta _*+d_{12}\int _{\mathbb {R}}K^{(0)}_1(s)ds. \end{aligned}$$

Define the function

$$\begin{aligned} F(\psi _1,\psi _2,K_1,K_2)=K_1 (1-K_2). \end{aligned}$$

By (5.15), we have that

$$\begin{aligned} \begin{aligned} {\mathcal {F}}_+(\tau _1,\tau _2)&= \int _{\mathbb {R}}F(\gamma _+(\tau _1+t,\tau _2+t))dt=(\tau _1-\tau _2)\frac{1}{1-e^{-\sqrt{3}(\tau _1-\tau _2)}},\\ {\mathcal {F}}_-(\tau _1,\tau _2)&= \int _{\mathbb {R}}F(\gamma _-(\tau _1+t,\tau _2+t))dt=-(\tau _1-\tau _2)\frac{1}{1-e^{\sqrt{3}(\tau _1-\tau _2)}}, \end{aligned} \end{aligned}$$

which is just the integral of the function *F* along the heteroclinic orbits $\gamma _\pm$ introduced in (5.7). These functions satisfies ${\mathcal {F}}_\pm (\tau _1,\tau _2)={\mathcal {F}}_\pm (0,\tau _2-\tau _1)$.

Since the homoclinic orbit (5.18) is “close” to the concatenation of $\gamma _+$ and $\gamma _-$ in (5.7), we show that there exists $\nu _0>0$ such that the integral in (5.25) satisfies

$$\begin{aligned} \begin{aligned} \int _{{\mathbb {R}}} K^{(0)}_1(s+\tau _0) \,(1-K^{(0)}_2(s))\,ds&={\mathcal {F}}_+(0,\tau _0)+{\mathcal {F}}_-(0,\tau _0)+{\mathcal {O}}\left( \delta ^{\nu _0}\right) \\&=\tau _0 {\mathrm {coth}} \left( \frac{\sqrt{3}\tau _0}{2} \right) +{\mathcal {O}}\left( \delta ^{\nu _0}\right) . \end{aligned} \end{aligned}$$

The estimate for the error is proved in the following lemma. To state it, we define

5.26 $$\begin{aligned} {\mathfrak {O}}_F(\mathbf {\tau }):=\!\!\int _{{\mathbb {R}}}\left[ F(\gamma _0(\tau _1+t,\tau _2+ t))-F(\gamma _+(\tau _1+t,\tau _2+ t))-F(\gamma _-(\tau _1+t,\tau _2+ t))\right] \,dt. \end{aligned}$$

##### Lemma 5.9

Let ${\mathbf {K}}$ be a compact subset of ${\mathbb {R}}^2$. There exists $\delta _0>0$ small, such that $\forall \delta \in (0, \delta _0)$ and $\tau \in {\mathbf {K}}$ there exists a positive constant $\nu _0\in (0, 1)$ such that the following holds

5.27 $$\begin{aligned} \Vert {\mathfrak {O}}_F \Vert _{C^0({\mathbf {K}})}+ \Vert \partial _{\tau _1} {\mathfrak {O}}_F \Vert _{C^0({\mathbf {K}})}+\Vert \partial ^2_{\tau _1} {\mathfrak {O}}_F \Vert _{C^0({\mathbf {K}})} \lesssim \delta ^{\nu _0}. \end{aligned}$$

This lemma implies Proposition 5.8. We devote the rest of this section to prove Lemma 5.9.

##### Proof of Lemma 5.9

We write the function ${\mathfrak {O}}_F$ in (5.26) as

$$\begin{aligned}&{\mathfrak {O}}_F(\mathbf {\tau }):=\int _{{\mathbb {R}}} F(\gamma _0(\tau _1-p+t, \tau _2-p+t)-F(\gamma _+(\tau _1+t,\\&\tau _2+t)-F(\gamma _-(\tau _1-2 p+t, \tau _2- 2 p+t))\,dt \end{aligned}$$

where

5.28 $$\begin{aligned} p:=\dfrac{1}{\sqrt{3}}\left| \ln \frac{\delta }{3}\right| . \end{aligned}$$

Note that the shifts by the vector (*p*, *p*) do not alter the value of the integral. These shifts are useful to bound the integrand. To obtain such estimates, we need the following lemmas.

##### Lemma 5.10

Let $\sigma _1\in (0, 1)$. Consider $\gamma _\pm , \gamma _0$ in (5.18), (5.7) and

5.29 $$\begin{aligned} {\mathcal {I}}:=\left( -\frac{\sigma _1}{\sqrt{3}}\left| \ln \frac{\delta }{3}\right| , \frac{\sigma _1}{\sqrt{3}} \left| \ln \frac{\delta }{3}\right| \right) . \end{aligned}$$

There exists a constant $\nu \in (0, 1)$ such that

5.30 $$\begin{aligned}&\left\Vert K_i^{(0)}\left( \tau _i\mp p+ t\right) -K_i^{\pm }(\tau _i+ t) \right\Vert _{C^0({\mathcal {I}}\times {\mathbf {K}})}\lesssim \delta ^{\nu }, \qquad i=1, 2, \end{aligned}$$

5.31 $$\begin{aligned}&\left\Vert \sin \left( \psi _i^{(0)}\left( \tau _i\mp p+ t\right) \right) -\sin (\psi _i^{\pm }(\tau + t)) \right\Vert _{C^0({\mathcal {I}}\times {\mathbf {K}})}\lesssim \delta ^{\nu }\qquad i=1, 2. \end{aligned}$$

##### Proof

To simplify the notation let us consider $i=1$. By (5.21) we have that

$$\begin{aligned} K_1^{(0)}\left( t\pm p\right) =\frac{1}{1+e^{\pm \sqrt{3}t}-\frac{\delta }{3}+\frac{\delta ^2}{9} e^{\mp \sqrt{3}t}}. \end{aligned}$$

Thus for $$\sigma \in [-\sigma _1, \sigma _1]$$,

$$\begin{aligned} \left|K_1^{(0)}\left( \frac{\sigma }{\sqrt{3}} \left| \ln \frac{\delta }{3}\right| \pm p\right) -K_1^\mp \left( \frac{\sigma }{\sqrt{3}}\left| \ln \frac{\delta }{3}\right| \right) \right|\lesssim \max \{ \delta , \delta ^{2-\sigma _1} \}. \end{aligned}$$

This gives the bounds (5.30). By using (5.20) and the trigonometric identity $\sin (2\arctan (x))=2 x/(1+x^2)$ we have

$$\begin{aligned} \sin (\psi ^{(0)}_1(t))=-\frac{\sqrt{3}\sinh \left( \sqrt{3}t\right) }{2\cosh \left( \sqrt{3}t\right) -1}=-\frac{\sqrt{3}}{2} \tanh (\sqrt{3} t)\Big ( 1+\frac{1}{2 \cosh (\sqrt{3} t)-1} \Big ). \end{aligned}$$

Then, for $t_\pm =\displaystyle \pm p+\frac{\sigma }{\sqrt{3}}\left| \ln \frac{\delta }{3}\right|$ with $\sigma \in [-\sigma _1, \sigma _1]$, we have

$$\begin{aligned} \tanh (\sqrt{3} t_{\pm })= \pm 1+{\mathcal {O}}\left( \delta ^{2(1-\sigma _1)}\right) ,\qquad \frac{1}{2 \cosh (\sqrt{3} t_{\pm })-1}={\mathcal {O}}\left( \delta ^{1-\sigma _1}\right) . \end{aligned}$$

To prove (5.31) it is enough to use these estimates and (5.7), to obtain

$$\begin{aligned} \sin (\psi ^{(0)}_1(t_\pm ))=\mp \frac{\sqrt{3}}{2}+{\mathcal {O}}\left( \delta ^{1-\sigma _1}\right) =\sin (\psi _i^{\mp }(\tau _i))+{\mathcal {O}}\left( \delta ^{1-\sigma _1}\right) . \end{aligned}$$

$$\square $$

##### Lemma 5.11

There exists $T>0$ independent of $\varepsilon$ such that (see (5.7), (5.18))

$$\begin{aligned} \begin{aligned} \Vert \gamma _+(\tau _1+t, \tau _2+t)-{\mathfrak {e}}_+^{(0)} \Vert _{C^0({\mathbf {K}})}&\lesssim \,e^{\sqrt{3} t}&\quad \text {for}\,\, t<0,&\\ \Vert \gamma _+(\tau _1+t, \tau _2+t)-{\mathfrak {e}}_+^{(1)} \Vert _{C^0({\mathbf {K}})}&\lesssim \,e^{-\sqrt{3} t}&\quad \text {for}\,\, t>0,&\\ \Vert \gamma _-(\tau _1+t, \tau _2+t)-{\mathfrak {e}}_-^{(1)} \Vert _{C^0({\mathbf {K}})}&\lesssim \,e^{\sqrt{3} t}&\quad \text {for}\,\, t<0,&\\ \Vert \gamma _-(\tau _1+t, \tau _2+t)-{\mathfrak {e}}_-^{(0)} \Vert _{C^0({\mathbf {K}})}&\lesssim \,e^{-\sqrt{3} t}&\quad \text {for}\,\, t>0,&\\ \Vert \gamma _0(\tau _1-p+t, \tau _2-p+t)-{\mathfrak {e}}^{(0)}_{+} \Vert _{C^0({\mathbf {K}})}&\lesssim \,e^{\sqrt{3}t}&\quad \text {for}\,\, t<-T,&\\ \Vert \gamma _0(\tau _1+p+t, \tau _2+p+t)-{\mathfrak {e}}^{(0)}_{-} \Vert _{C^0({\mathbf {K}})}&\lesssim \,e^{-\sqrt{3}t}&\quad \text {for}\,\, t>T&. \end{aligned} \end{aligned}$$

##### Proof

The lemma follows by straightforward estimates and the hyperbolicity of the equilibria. $\quad \square$

We split $${\mathbb {R}}=\bigcup _{k=1}^5 {\mathcal {I}}_k$$

$$\begin{aligned} {\mathcal {I}}_1:=(-\infty , \mathtt {a}), \quad {\mathcal {I}}_2:=[\mathtt {a}, \mathtt {b}], \quad {\mathcal {I}}_3:=[\mathtt {b}, \mathtt {c}], \quad {\mathcal {I}}_4:=[\mathtt {c}, \mathtt {d}], \quad {\mathcal {I}}_5:=(\mathtt {d}, +\infty ) \end{aligned}$$

with

$$\begin{aligned} \mathtt {a}=-\frac{3}{4\sqrt{3}} \left| \ln \delta \right| ,\quad \mathtt {b}=\frac{3}{4\sqrt{3}}\left| \ln \delta \right| , \quad \mathtt {c}=2p-\frac{3}{4\sqrt{3}}\left| \ln \delta \right| , \quad \mathtt {d}=2p+\frac{3}{4\sqrt{3}}\left| \ln \delta \right| . \end{aligned}$$

We have

$$\begin{aligned}&\int _{{\mathbb {R}}}\left[ F(\gamma ( \tau _1-p +t, \tau _2-p+t))-F(\gamma _+(\tau _1+t, \tau _2+t))\right. \\&\quad \left. -\,F(\gamma _-( \tau _1-2p +t, \tau _2-2p+t))\right] \,dt=\sum _{j=1}^5\mathtt {T}_j \end{aligned}$$

where

5.32 $$\begin{aligned} \mathtt {T}_1=&\,\int _{{\mathcal {I}}_1} \left[ F(\gamma _0( \tau _1-p +t, \tau _2-p+t))-F(\gamma _+(\tau _1+t, \tau _2+t))\right] \,dt \end{aligned}$$

5.33 $$\begin{aligned} \mathtt {T}_2=&\,\int _{{\mathcal {I}}_2}\left[ F(\gamma _0( \tau _1-p +t, \tau _2-p+t))-F(\gamma _+(\tau _1+t, \tau _2+t))\right] \,dt \end{aligned}$$

5.34 $$\begin{aligned} \mathtt {T}_3=&\,\int _{{\mathcal {I}}_3} F(\gamma _0( \tau _1-p +t, \tau _2-p+t))\,dt-\int _{\mathtt {b}}^{+\infty } F(\gamma _+(\tau _1+t, \tau _2+t))\,dt \end{aligned}$$

5.35 $$\begin{aligned}&- \int ^{\mathtt {c}}_{-\infty } F(\gamma _-(\tau _1-2 p+t, \tau _2-2 p+t))\,dt \nonumber \\ \mathtt {T}_4=&\,\int _{{\mathcal {I}}_4} \left[ F(\gamma _0( \tau _1-p +t, \tau _2-p+t))-F(\gamma _-(\tau _1-2 p+t, \tau _2-2 p+t))\right] \,dt \end{aligned}$$

5.36 $$\begin{aligned} \mathtt {T}_5=&\,\int _{{\mathcal {I}}_5}\left[ F(\gamma _0( \tau _1-p +t, \tau _2-p+t))-F(\gamma _-(\tau _1-2 p+t, \tau _2-2 p+t))\right] \,dt. \end{aligned}$$

By the symmetry of the problem it is sufficient to provide bounds for $|\mathtt {T}_i |$ with $i=1, 2, 3$. The idea is to use the exponentially fast convergence of the orbits $\gamma _0$, $\gamma _{\pm }$ to the saddles (see Lemma 5.11) to get bounds on the integrals over the unbounded intervals and to exploit the closeness of such orbits on the compact intervals using Lemma 5.10.

- **Bound for** $\mathtt {T}_1$ (see (5.32)): Let us call $S:=\{ \gamma _0( \tau _1-p +t, \tau _2-p+t) \}_{t\in {\mathcal {I}}_1}$. *S* is a compact subset of ${\mathbb {R}}^2$. Recalling that $F({\mathfrak {e}}^{(0)}_+)=0$, by Lemma 5.11 and the mean value theorem we have that (recall (5.18))
  5.37 $$\begin{aligned} \begin{aligned} \int _{{\mathcal {I}}_1} |F(\gamma _0( \tau _1-p +t, \tau _2-p+t)) |\,dt&=\int _{{\mathcal {I}}_1} |F(\gamma _0( \tau _1-p +t, \tau _2-p+t))-F({\mathfrak {e}}^{(0)}_+) |\,dt\\&\le \Vert F \Vert _{C^1(S)} \int _{{\mathcal {I}}_1} \Vert \gamma _0( \tau _1-p +t, \tau _2-p+t)-{\mathfrak {e}}_+^{(0)}\Vert \,dt\\&\lesssim \delta ^{3/4}. \end{aligned} \end{aligned}$$
  By Lemma 5.11, the compactness of the orbit $\{\gamma _+(\tau _1+t, \tau _2+t)\}_{t\in {\mathcal {I}}_1}$, $F({\mathfrak {e}}_+^{(0)})=0$ one can reason in the same way to obtain the same bound for the term involving $\gamma _+$.
- **Bound for** ${{\mathtt {T}}}_2$ (see (5.33)): We note that ${\mathcal {I}}_2$ is of the form (5.29). By using the compactness of the orbits and the mean value theorem as in the previous step, we can apply Lemma 5.10 and obtain
  5.38 $$\begin{aligned} |\mathtt {T}_2 |\lesssim \delta ^{\nu }\,|{\mathcal {I}}_2 |\lesssim \delta ^{\nu }\,|\ln \delta |\end{aligned}$$
  where $\nu \in (0, 1)$ is given by Lemma 5.10.
- **Bound for** ${{\mathtt {T}}}_3$ (see (5.34)): We use that $F( 1, 1)=0$. Let us denote $\mathtt {m}:=-(\mathtt {b}-p)=\mathtt {c}-p>0$ (see (5.28) for the definition of *p*). By translating the variable *t* we obtain
  $$\begin{aligned} \int _{{\mathcal {I}}_3} |F(\gamma _0(\tau _1- p+t, \tau _2- p+t)) |\,dt&=\int _{{\mathcal {I}}_3} |F(\gamma _0(\tau _1- p+t, \tau _2- p+t))-F( 1, 1) |\,dt\\&=\int _{-\mathtt {m}}^{\mathtt {m}} |F(\gamma _0(\tau _1+t, \tau _2+t))-F(1, 1) |\,dt\\&\lesssim \Vert F \Vert _{C^1(S)} \sum _{i=1}^2 \int _{-\mathtt {m}}^{\mathtt {m}} | K_i^{(0)} (t)-1 |\,dt. \end{aligned}$$
  Now, using (5.21), one can see that on the interval $[-\mathtt {m}, \mathtt {m}]$, one has that $| K_i^{(0)} (t)-1 |\lesssim \delta ^{3/4}$, which implies
  $$\begin{aligned} \int _{{\mathcal {I}}_3} |F(\gamma _0(\tau _1- p+t, \tau _2- p+t)) |\,dt\lesssim \delta ^{3/4} \,|\ln \delta |. \end{aligned}$$
  By Lemma 5.11 we have
  $$\begin{aligned} \begin{aligned} \int _{\mathtt {b}}^{\infty } |F(\gamma _+(\tau _1+t, \tau _2+t))|\,dt&\lesssim \delta ^{3/4}\\ \int ^{\mathtt {c}}_{-\infty } |F(\gamma _-(\tau _1- 2p+t, \tau _2- 2p+t)) |\,dt&= \int ^{\mathtt {a}}_{-\infty } |F(\gamma _-(\tau _1+t, \tau _2+t)) |\,dt\lesssim \delta ^{3/4}. \end{aligned} \end{aligned}$$
  Hence,
  5.39 $$\begin{aligned} |\mathtt {T}_3|\lesssim \delta ^{3/4} \,|\ln \delta |. \end{aligned}$$

By (5.37), (5.38), (5.39) we have that

$$\begin{aligned} \Vert {\mathfrak {O}}_F \Vert _{C^0({\mathcal {A}})}\lesssim \delta ^{\nu }|\ln \delta |\end{aligned}$$

(changing $\nu$ if necessary). Now we observe that (recall (5.1), (5.16))

$$\begin{aligned} \partial _{\tau _1} F(\gamma _0(\tau _1- p+t, \tau _2- p+t))&=\{ F, {\mathbf {H}}^{(1)}_0\} (\gamma _0(\tau _1- p+t, \tau _2- p+t))\\ \partial _{\tau _1} F(\gamma _+(\tau _1+t, \tau _2+t))&=\{ F, {\mathcal {H}}^{(1)}_0 \}(\gamma _+(\tau _1+t, \tau _2+t)), \\ \partial _{\tau _1} F(\gamma _-(\tau _1-2 p+t, \tau _2-2 p+t))&=\{ F, {\mathcal {H}}^{(1)}_0\}(\gamma _-(\tau _1-2 p+t, \tau _2-2 p+t)). \end{aligned}$$

By the particular form of the Hamiltonians ${\mathcal {H}}^{(1)}_0$ and ${\mathbf {H}}^{(1)}_0$ in (5.1), (5.16) one can check that $\{ F, {\mathcal {H}}^{(1)}_0\}=\{ F, {\mathbf {H}}^{(1)}_0\}$. Let us call $G:=\{ F, {\mathcal {H}}^{(1)}_0\}$. Clearly $G({\mathfrak {e}}_\pm ^{(0)})=G({\mathfrak {e}}_\pm ^{(1)})=0$. Now we can repeat the same strategy to get the bounds for the associated $\mathtt {T}_i$. The only difference is that when we compare the orbits $\gamma _0, \gamma _{\pm }$ on compact intervals we need to use also (5.31). Then we obtain $\Vert {\mathfrak {O}}_{G} \Vert _{C^0({\mathbf {K}})}\lesssim \delta ^{\nu }|\ln \delta |$ (recall (5.26)).

Regarding the second derivatives in $\tau _1$ we have

$$\begin{aligned} \partial _{\tau _1}^2 F(\gamma _0(\tau _1- p+t, \tau _2- p+t))&=\{ G, {\mathbf {H}}^{(1)}_0\} (\gamma _0(\tau _1- p+t, \tau _2- p+t))\\ \partial _{\tau _1}^2 F(\gamma _+(\tau _1+t, \tau _2+t))&=\{ G, {\mathcal {H}}^{(1)}_0 \}(\gamma _+(\tau _1+t, \tau _2+t)), \\ \partial _{\tau _1}^2 F(\gamma _-(\tau _1-2 p+t, \tau _2-2 p+t))&=\{ G, {\mathcal {H}}^{(1)}_0\}(\gamma _-(\tau _1-2 p+t, \tau _2-2 p+t)). \end{aligned}$$

We observe that on a compact set $|\{G, {\mathbf {H}}^{(1)}_0\}-\{ G, {\mathcal {H}}^{(1)}_0\}|\lesssim \delta$. Then we can consider the function $E:=\{G, {\mathcal {H}}^{(1)}_0\}$ and repeat the same arguments above to prove that $\Vert {\mathfrak {O}}_{E} \Vert _{C^0({\mathbf {K}})}$ has a bound like (5.27). We conclude by noting that

$$\begin{aligned}&\Vert {\mathfrak {O}}_F \Vert _{C^0({\mathbf {K}})}+ \Vert \partial _{\tau _1} {\mathfrak {O}}_F \Vert _{C^0({\mathbf {K}})}+\Vert \partial ^2_{\tau _1} {\mathfrak {O}}_F \Vert _{C^0({\mathbf {K}})} \lesssim \Vert {\mathfrak {O}}_F \Vert _{C^0({\mathbf {K}})}\\&\quad +\Vert {\mathfrak {O}}_{G} \Vert _{C^0({\mathbf {K}})}+\Vert {\mathfrak {O}}_{E} \Vert _{C^0({\mathbf {K}})}. \end{aligned}$$

$$\square $$

### Transversal homoclinic orbits to saddles: *N* resonant tuples

In this section we prove the generalization of Theorem 5.2 for the case of multiple resonant tuples. To break integrability we need to impose a non-degeneracy condition on the coefficients $d_{ij}$ in (4.23). To state it we introduce the matrix

5.40 $$\begin{aligned} {\mathcal {D}} = \begin{pmatrix} d_{1,N} + \sum _{j \ne 1} d_{1,j} &{} \quad -d_{1,2} &{}\quad \ldots &{}\quad -d_{1,N-1} \\ -d_{2,1} &{}\quad \ddots &{} \quad \vdots &{}\quad \vdots \\ \vdots &{} \quad \vdots &{}\quad \ddots &{}\quad -d_{N-2,N-1} \\ -d_{N-1,1} &{}\quad \ldots &{}\quad -d_{N-1,N-2} &{}\quad d_{N-1,N} + \sum _{j \ne N-1} d_{N-1,j} \end{pmatrix}. \end{aligned}$$

#### Proposition 5.12

Assume that the matrix ${\mathcal {D}}$ satisfies

5.41 $$\begin{aligned} \det {\mathcal {D}}\ne 0. \end{aligned}$$

Then, there exists $\varepsilon _0>0$ such that for all $\varepsilon \in (0, \varepsilon _0)$ the invariant manifolds $W_{\varepsilon }^-({\mathfrak {e}}^{(0)}_-)$ and $W_{\varepsilon }^+({\mathfrak {e}}^{(0)}_+)$ of the saddles (5.43) of the Hamiltonian (5.42) intersect transversally along an orbit (within the energy level).

#### Remark 5.13

Note that condition (5.41) is satisfied for a generic choice of coefficients $d_{ij}$. Indeed, the determinant of such matrix is a polynomial in the variables $d_{i j}$. Then, it is enough to show that such polynomial is not identically zero. If one consider

$$\begin{aligned} d_{i j}={\left\{ \begin{array}{ll} 1 \quad \text {if}\,\,i=1, \dots , N-1,\,\,j=N,\\ 0 \quad \text {otherwise} \end{array}\right. } \end{aligned}$$

the matrix (5.40) is a multiple of the identity. This means that at some point the polynomial is not zero and therefore it is not-zero for almost every choice of $d_{ij}$. In Sect. 7.2, we prove that condition (5.41) is satisfied for the resonant models associated to the Wave, Beam and Hartree equations that we consider.

#### Proof

We proceed as for the case $N=2$ in Sect. 5.2.2. That is, we introduce a second parameter $\delta$ and we define the Hamiltonian ${\mathcal {H}}=\sum _{j=1}^N{\mathbf {H}}_0^{(j)}+\varepsilon {\mathbf {H}}_1$ given by (recall (4.23))

5.42 $$\begin{aligned} \begin{aligned} {\mathbf {H}}_0^{(j)}(\psi _j, K_j; \delta ) :=&\,K_j (1-K_j)(1 +2 \,\cos (\psi _j))-\delta \, K^2_j ,\\ {\mathbf {H}}_1(\psi _1, \dots , \psi _N, K_1, \dots , K_N) :=&\,\sum _{j=1}^N \big (a_{j} K_j + (b_{j}+1) K^2_j +c_j\,K_j (1-K_j)\,\cos (\psi _j)\big )\\&\,+ \sum _{i, j=1, i< j}^N d_{i j} K_i\,K_j. \end{aligned} \end{aligned}$$

If $\delta =\varepsilon$, it coincides with (4.22).

We proceed as in the proof of Theorem 5.2. For $\varepsilon =0$ the dynamics is the same described in Sect. 5.2.2. In particular it is easy to see that, when $\varepsilon =0$, one can consider the two saddle points (recall that $\Psi _*:=2\pi /3$)

5.43 $$\begin{aligned} {\mathfrak {e}}^{(0)}_{\pm }:=(\pm \Psi _*, \dots , \pm \Psi _*, 0, \dots , 0) \end{aligned}$$

connected by the $\delta$-dependent homoclinic manifolds (recall (5.20), (5.18))

$$\begin{aligned} \gamma _0(\mathbf {\tau }):=(\psi _1^{(0)}(\tau _1), \ldots , \psi _N^{(0)}(\tau _N), K_1^{(0)}(\tau _1), \ldots , K_N^{(0)}(\tau _N)),\qquad \mathbf {\tau }=(\tau _1,\ldots ,\tau _N). \end{aligned}$$

We define the associated Melnikov potential

5.44 $$\begin{aligned} {\mathcal {L}}_{0, N}(\mathbf {\tau }):=\int _{{\mathbb {R}}} {\mathbf {H}}_1\circ \Phi ^t_{{\mathbf {H}}_0} (\gamma _0(\mathbf {\tau }))\,dt= \sum _{i, j=1, i< j}^N d_{i j} \int _{{\mathbb {R}}} K^{(0)}_i(\tau _i+t)\,K^{(0)}_j(\tau _j+t) \,dt+\eta _*, \end{aligned}$$

where

$$\begin{aligned} \eta _*:=\sum _{i=1}^N\int _{{\mathbb {R}}} a_{i} K^{(0)}_i(t) + (b_{i}+1) (K^{(0)}_i)^2(t) +c_i\,K^{(0)}_i (1-K^{(0)}_i(t))\,\cos \psi ^{(0)}_i(t)\,dt. \end{aligned}$$

We note that such function is the sum of terms of the form (5.24). Thanks to the autonomous nature of the system the potential, ${\mathcal {L}}_{0, N}$ depends just on $\tau _1-\tau _N, \dots , \tau _{N-1}-\tau _N$. Thus one can consider the reduced Melnikov potential ${\mathcal {L}}_{0, N}^{(0)}$, which satisfies

$$\begin{aligned} {\mathcal {L}}_{0, N}^{(0)}\left( \tau _1-\tau _N, \dots , \tau _{N-1}-\tau _N\right) ={\mathcal {L}}_{0, N}(\tau _1,\ldots ,\tau _N). \end{aligned}$$

Classical Melnikov Theory ensures that non-degenerate critical points of this reduced Melnikov potential gives rise to transversal (within the energy level) intersections between $W_{\varepsilon }^-({\mathfrak {e}}^{(0)}_-)$ and $W_{\varepsilon }^+({\mathfrak {e}}^{(0)}_+)$.

Denoting $\,\,{{\tilde{\tau }}}:= (\tau _1-\tau _N, \dots , \tau _{N-1}-\tau _N)$, Proposition 5.8 implies that there exists a constant $\eta \in {\mathbb {R}}$ such that

$$\begin{aligned} {\mathcal {L}}_{0, N}^{(0)}({\tilde{\tau }})=\eta +\sum _{i, j=1, i< j}^{N-1} d_{i j}\, ({\tilde{\tau }}_i-{\tilde{\tau }}_j)\coth \left( \frac{\sqrt{3}}{2} ({\tilde{\tau }}_i-{\tilde{\tau }}_j) \right) +\sum _{j=1}^{N-1} d_{j N} {\tilde{\tau }}_j \coth \left( \frac{\sqrt{3}}{2} {\tilde{\tau }}_j \right) +{\mathcal {O}}_{C^2}(\delta ^{\nu _0}) \end{aligned}$$

for some $\nu _0>0$. Since $x\,\coth ((\sqrt{3}/2)x)$ is an even function, the origin $(0, \dots , 0)\in {\mathbb {R}}^{N-1}$ is a critical point of the first order of ${\mathcal {L}}_{0, N}^{(0)}$ (that is, dropping the errors ${\mathcal {O}}_{C^2}(\delta ^{\nu _0})$). The Hessian matrix of the first order of ${\mathcal {L}}_{0, N}^{(0)}$ at the origin is

$$\begin{aligned} {\mathrm {Hess}}= \frac{1}{\sqrt{3}}{\mathcal {D}} \end{aligned}$$

where ${\mathcal {D}}$ is the matrix introduced in (5.40). Then, condition (5.41) implies $\det {\mathrm {Hess}}\ne 0$.

The non-degeneracy of the Hessian implies that the reduced Melnikov potential ${\mathcal {L}}_{0, N}^{(0)}$ has a non-degenerate critical point $\delta ^{\nu _0}$–close to ${{\tilde{\tau }}}=0$. Then, taking $\delta =\varepsilon$ one can use classical Melnikov Theory to ensure the existence of the transverse intersection between invariant manifolds stated in Proposition 5.12. $\quad \square$

## Proof of Theorem 1.3

The goal of this section is to prove Theorem 1.3. The key point of the proof is to construct symbolic dynamics (an infinite symbols Smale horseshoe) for the resonant model (5.1) which has been derived from the equations (1.10), (1.1), (1.2). In Theorem 5.2 we have constructed transverse homoclinic orbits to saddles for (5.1). It is well known that the intersection of invariant manifolds of critical points in flows do not always lead to the existence of symbolic dynamics (see, for instance, [11]). Therefore, the first step of the proof is to obtain transverse homoclinic points to certain periodic orbits. This is done in Sect. 6.1. Then, following [37], in Sect. 6.2 we construct an invariant set of (a suitable Poincaré map of) the flow associated to the Hamiltonian (5.1) whose dynamics is conjugated to a shift of infinite symbols (see Sect. 2). Finally in Sect. 6.3 we complete the proofs of Theorem  1.3 by checking that the non-degeneracy conditions imposed on (5.1) are satisfied for the resonant models obtained from the PDEs (1.1), (1.10) and (1.2).

### Transversality of invariant manifolds of periodic orbits

The main result in this section is the following.

#### Proposition 6.1

Consider the Hamiltonian (5.1) and assume that (5.9) holds. Then there exists $\varepsilon _0>0$ such that for all $\varepsilon \in (0, \varepsilon _0)$ there exists $h_0=h_0(\varepsilon )>0$ such that for all $h\in (0, h_0)$,

(i) The hyperbolic periodic orbits $\mathtt {P}^{\pm , 0 }_h$ in (5.8) persist and have period $\mathtt {T}_h$. That is, Hamiltonian (5.1) has hyperbolic periodic orbits $\mathtt {P}^{\pm , 0 }_{h,\varepsilon }$ that are ${\mathcal {O}}(\varepsilon )$–close to $\mathtt {P}^{\pm , 0}_h$.
(ii) The invariant manifolds $W^u(\mathtt {P}^{+, 0 }_{h,\varepsilon })$ and $W^s(\mathtt {P}^{-, 0 }_{h,\varepsilon })$ intersect transversally along orbits (within the energy level).

Note that in the coordinates introduced in (4.25), the periodic orbits $\mathtt {P}^{+, 0 }_{h,\varepsilon }$ and $\mathtt {P}^{-, 0 }_{h,\varepsilon }$ blow down to the same periodic orbit, which we denote by $\mathtt {P}^{0 }_{h,\varepsilon }$. In the coordinates (4.25), Proposition 6.1 can be restated as that the manifolds $W^u(\mathtt {P}^{0 }_{h,\varepsilon })$ and $W^s(\mathtt {P}^{0 }_{h,\varepsilon })$ intersect transversally within the energy level.

#### Proof of Proposition 6.1

To prove (i) is more convenient to use the cartesian coordinates $\{x_j,y_j\}$ in (4.25) and therefore Hamiltonian ${{\widetilde{H}}}_\mathrm {Res}$ in (4.26) (with $N=2$) to avoid the blow up of $K_j=0$. Then, the invariant subspace $\{K_2=0\}$ corresponds to $\{x_2=y_2=0\}$. The Hamiltonian on this invariant subspace is given by

$$\begin{aligned} \begin{aligned} {\mathcal {H}}( x_1, 0,y_1, 0)=&\frac{1}{2}\left( 3x_1^2-y_1^2\right) - \frac{1}{4} \left( 3x_1^2-y_1^2\right) \left( x_1^2+y_1^2\right) \\&+\varepsilon \Big [ \frac{1}{2} a_{1} \left( x_1^2+y_1^2\right) +\frac{1}{4} b_{1} \left( x_1^2+y_1^2\right) ^2 +\frac{1}{4}c_1 \left( x_1^2+y_1^2\right) \left( 2-x_1^2-y_1^2\right) \Big ]. \end{aligned} \end{aligned}$$

This Hamiltonian is integrable both for $\varepsilon =0$ and $\varepsilon >0$ and has the saddle (0, 0) at the energy level. Integrability and the particular form of ${\mathcal {H}}$ implies that the energy levels close to zero are given by periodic orbits. These periodic orbits are $\varepsilon$-close to those of the unperturbed problem (see (5.1)).

To prove (ii) we proceed as in Sect. 5 by doing approximations of several Melnikov functions and using an auxiliary parameter $\delta$. We follow the notation of Sect. 5.2.2, In particular, we consider the Hamiltonians ${\mathbf {H}}_0$, ${\mathbf {H}}_1$ in (5.16), which taking $\delta =\varepsilon$ also define the Hamiltonian ${\mathcal {H}}$.

By the particular form of Hamiltonian ${\mathbf {H}}_0^{(j)}$, $j=1,2$ (see (5.16), it can be easily checked that it has the saddles $(\pm \Psi _*,0)$ (they correspond to $x_1=y_1=0$ in the blow down coordinates (4.25). These saddles are connected by the homoclinic orbits $\gamma _0^{(j)}$, $j=1, 2$, introduced in (5.18).

Let $h>0$ small, then the Hamiltonian ${\mathbf {H}}_0$ possesses the hyperbolic periodic orbits

$$\begin{aligned} \mathtt {P}^{\pm ,0}_{\delta ,h} =\left\{ \big (\gamma _{\delta ,h}^{(1)}(\tau ),\pm \Psi _*,0 \big ) : \tau \in {\mathbb {R}}\right\} , \end{aligned}$$

where $\gamma _{\delta ,h}^{(k)}$ is the time parametrization of the periodic orbit defined by $\{{\mathbf {H}}_0^{(k)}=h\}$ (see Fig. 6). When $\varepsilon = 0$, the homoclinic manifold $W^u_0(\mathtt {P}_{\delta ,h}^{+,0})\equiv W^s_0(\mathtt {P}_{\delta ,h}^{-,0})$ is parameterized by

6.1 $$\begin{aligned} \Gamma _{\delta ,h,0}(\mathbf {\tau }) := (\gamma _{\delta ,h}^{(1)}(\tau _1),\gamma _{0}^{(2)}(\tau _2)). \end{aligned}$$

#### Remark 6.2

The periodic orbits $\mathtt {P}^{\pm ,0}_{\delta ,h}$ converge pointwise for any fixed $\tau$ to $(\gamma _0^{(1)}(\tau ),\pm \Psi _*,0)$ as $h\rightarrow 0$. Similarly, for fixed $\tau$, the parametrization $\Gamma _{\delta ,h,0}(\mathbf {\tau })$ converges to $\gamma _0(\mathbf {\tau })$ in (5.18) as $h\rightarrow 0$.

When $\varepsilon >0$, the periodic orbits $\mathtt {P}_{\delta ,h}^{\pm ,0}$ persist . Direct application of Melnikov Theory, as in Sect. 5, ensures the following. There exists $\delta _0>0$, $\varepsilon _0>0$ small enough such that for any $\delta \in (0,\delta _0)$ and $\varepsilon \in (0,\varepsilon _0)$ small enough, the distance function between the invariant manifolds $W^u(\mathtt {P}^{+, 0 }_{h,\varepsilon })$ and $W^s(\mathtt {P}^{-, 0 }_{h,\varepsilon })$ in a well chosen transversal section is given by

$$\begin{aligned} d(\mathbf {\tau })=\varepsilon \frac{{\mathcal {M}}_h(\mathbf {\tau })}{\Vert \nabla {\mathbf {H}}_0^{(2)}(\gamma _{0}^{(2)} (\tau _2)) \Vert }+{\mathcal {O}}\left( \varepsilon ^2\right) , \end{aligned}$$

where the error ${\mathcal {O}}(\varepsilon ^2)$ is uniform in $\delta$ and *h* and ${\mathcal {M}}_h$ is the Melnikov function given by

6.2 $$\begin{aligned} {\mathcal {M}}_h(\tau _1,\tau _2)=\int _\mathbb {{\mathbb {R}}}\left\{ {\mathbf {H}}_0^{(2)},{\mathbf {H}} _1\right\} (\Gamma _{\delta ,h,0}(\tau _1+t,\tau _2+t))dt. \end{aligned}$$

We note that as $t\rightarrow \pm \infty$ the $K_2$-component of $\Gamma _{\delta ,h,0}(\tau _1+t,\tau _2+t)$ goes exponentially fast to zero, then by a direct computation it is easy to see that

$$\begin{aligned} \lim _{\tau _2 \rightarrow \pm \infty } \{ {\mathbf {H}}_0^{(2)}, {\mathbf {H}}_1 \}(\gamma _{\delta ,h}^{(1)}(\tau _1),\gamma _{0}^{(2)}(\tau _2))=0 \end{aligned}$$

with exponentially fast convergence and (6.2) is well defined. To obtain the non-degeneracy of the zeros of the Melnikov function, we compare (6.2) to the Melnikov function (5.23) associated to the homoclinic orbits to the saddles ${\mathfrak {e}}_{\pm }^{(0)}$.

Let us consider the reduced Melnikov functions ${\mathcal {M}}_h^{(0)}(\tau _0)={\mathcal {M}}_h(\tau _1, \tau _2)$ and ${\mathcal {M}}_0^{(0)}(\tau _0)={\mathcal {M}}_0(\tau _1, \tau _2)$, where $\tau _0:=\tau _1-\tau _2$ (recall (5.23)).

#### Lemma 6.3

Let ${\mathbf {K}}\subset {\mathbb {R}}$ be a closed interval and let us define

$$\begin{aligned} {\mathfrak {O}}_h(\tau _0):={\mathcal {M}}_0^{(0)}(\tau _0)-{\mathcal {M}}_h^{(0)}(\tau _0). \end{aligned}$$

There exists $\delta _0>0$ small such that $\forall \delta \in (0, \delta _0)$ there exist $h_0=h_0(\delta )$, positive and small, such that $\forall h\in (0, h_0)$ there exists $\nu _*\in (0, 1)$ such that the following holds

$$\begin{aligned} \Vert {\mathfrak {O}}_h \Vert _{C^0({\mathbf {K}})}+ \Vert \partial _{\tau _0} {\mathfrak {O}}_h \Vert _{C^0({\mathbf {K}})} \lesssim \delta ^{\nu _*}. \end{aligned}$$

#### Proof

We consider the splitting

6.3 $$\begin{aligned} {\mathfrak {O}}_h(\tau _0)=&\int _{|t |>\mathtt {c}|\ln \delta |} \{ {\mathbf {H}}_0^{(2)}, {\mathbf {H}}_1 \}(\gamma _0(t,\tau _0+t))- \{ {\mathbf {H}}_0^{(2)}, {\mathbf {H}}_1 \}(\Gamma _{\delta ,h,0}(t,\tau _0+t))\,dt \end{aligned}$$

6.4 $$\begin{aligned}&+\int _{|t |\le \mathtt {c}|\ln \delta |} \{ {\mathbf {H}}_0^{(2)}, {\mathbf {H}}_1 \}(\gamma _0(t,\tau _0+t))- \{ {\mathbf {H}}_0^{(2)}, {\mathbf {H}}_1 \}(\Gamma _{\delta ,h,0}(t,\tau _0+t))\,dt, \end{aligned}$$

where $\mathtt {c}$ is some positive constant. We observe that

$$\begin{aligned} \{ {\mathbf {H}}_0^{(2)}, {\mathbf {H}}_1\}_{|_{\{K_1=0\}}}=0 \qquad \text{ and } \qquad \mathtt {P}_{\delta , h}^{\pm ,0}\subset \{ K_2=0\}. \end{aligned}$$

Then, by the exponential convergence of the flow to the hyperbolic saddles ${\mathfrak {e}}^{\pm }_0$ and the hyperbolic periodic orbits $\mathtt {P}_{\delta , h}^{\pm ,0}$, the term on the r. h. s. of (6.3) is bounded by $C\delta ^{\mathtt {d}}$ where $C, \mathtt {d}>0$ are two constants independent of *h*. Let us call ${\mathcal {I}}:=[-\mathtt {c}|\ln \delta |, \mathtt {c}|\ln \delta |]$. By Remark 6.2 we have

$$\begin{aligned} \lim _{h\rightarrow 0} \sup _{(\tau _0, t)\in {\mathbf {K}}\times {\mathcal {I}}} |\gamma _0(t,\tau _0+t)-\Gamma _{\delta ,h,0}(t,\tau _0+t) |=\lim _{h\rightarrow 0} \sup _{(\tau _0, t)\in {\mathbf {K}}\times {\mathcal {I}}} |(\gamma _0^{(1)}(t)-\gamma _{\delta , h}^{(1)}(t),0) |=0. \end{aligned}$$

Hence there exists $h_0=h_0(\delta )>0$ such that if $h\in (0, h_0)$ then (6.4) is bounded, up to constant factors, by $\delta$. The derivative $\partial _{\tau _0} {\mathfrak {O}}_h$ has the expression (6.3), (6.4) with the double Poisson $\{ {\mathbf {H}}_0^{(2)}, \{ {\mathbf {H}}_0^{(2)}, {\mathbf {H}}_1 \} \}$ instead of $\{ {\mathbf {H}}_0^{(2)}, {\mathbf {H}}_1 \}$. Clearly it is still true that this Poisson vanishes at $\{K_2=0\}$. Then one can repeat the same argument to get a bound as for ${\mathfrak {O}}_h$. $\quad \square$

Lemma 6.3 and Proposition 5.8 imply that ${\mathcal {M}}_h$ has a non-degenerate zero. Then, proceeding as in Sect. 5.2.2 and taking $\varepsilon =\delta$ one obtains transverse heteroclinic orbits between the periodic orbits $\mathtt {P}^{+, 0 }_{h,\varepsilon }$ and $\mathtt {P}^{-, 0 }_{h,\varepsilon }$. This completes the proof of Proposition 6.1. $\quad \square$

### Symbolic dynamics of infinite symbols

To construct symbolic dynamics for Hamiltonian (5.1) we consider a section transverse to the flow, within a given energy level, and the associated Poincaré map.

We proceed as in [37]. Fix $0<\varepsilon \ll 1$ and $0<h\ll 1$. We build an invariant set with points arbitrarily close to the transverse homoclinic orbit to the $\mathtt {T}_h$-periodic orbit $\mathtt {P}^{0 }_{h,\varepsilon }$ obtained in Proposition 6.1.

**Fig. 7:**
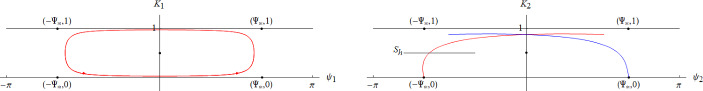
The periodic orbit in the $(\Psi _1,K_1)$-plane, its invariant manifolds and the section ${\mathcal {S}}_h$

We define the section within the energy level $\{{\mathcal {H}}=h\}$,

6.5 $$\begin{aligned} {\mathcal {S}}_h=\{K_2=1/2,\,\, \psi _2\in (-2\pi /3-m,-2\pi /3+m),\,\, {\mathcal {H}}(\Psi _1,\Psi _2,K_1,K_2)=h \}, \end{aligned}$$

for some small $m>0$ (see Fig. 7). This section is transverse to the unperturbed flow ($\varepsilon =0$) and therefore also transverse, for $\varepsilon >0$ small enough, to the perturbed one. In particular, by Proposition 6.1, it contains points in $W^u(\mathtt {P}^{0 }_{h,\varepsilon })\cap W^s(\mathtt {P}^{0 }_{h,\varepsilon })$ (classical perturbative arguments ensure that the perturbed invariant manifolds are ${\mathcal {O}}(\varepsilon )$ to the unperturbed ones).

Denote by $\Phi ^t_{\mathcal {H}}$ the flow associated to the Hamiltonian (5.1). For a point $z\in {\mathcal {S}}_h$, we define $T(z)>0$ the first (forward) return time of the trajectory $\Phi ^t_{\mathcal {H}}(z)$ to this section whenever it is defined. For those points whose forward trajectory never hits again ${\mathcal {S}}_h$ we can take $T(z)=+\infty$. Note that this happens in particular for the points in $W^s(\mathtt {P}^{-, 0 }_{h,\varepsilon })$ (note that by the perturbative results in Sect. 6.1 this intersection is not empty).

Then, we define the open set ${\mathcal {U}}\subset {\mathcal {S}}_h$ as ${\mathcal {U}}=\{z\in {\mathcal {S}}_h:T(z)<+\infty \}$ and the associated Poincaré map $\mathtt {P}:{\mathcal {U}}\subset {\mathcal {S}}_h\rightarrow {\mathcal {S}}_h$ defined by

$$\begin{aligned} \mathtt {P}(z):=\Phi ^{T(z)}_{{\mathcal {H}}} (z). \end{aligned}$$

#### Proposition 6.4

(Existence of Horseshoe) Assume (5.9). Then there exists $\varepsilon _0>0$ such that $\forall \varepsilon \in (0, \varepsilon _0)$ the Poincaré map $\mathtt {P}$ possesses an invariant set $Y\subset {\mathcal {U}}$ whose dynamics is conjugated to the infinite symbols shift. Namely, there exists a homeomorphism $h:\Sigma \rightarrow Y$, where

$$\begin{aligned} \Sigma ={{\mathbb {N}}}^{\mathbb {Z}}=\left\{ \{\omega _k\}_{k\in {\mathbb {Z}}}:\omega _k\in {{\mathbb {N}}}\right\} , \end{aligned}$$

such that $\mathtt {P}|_Y=h\circ \sigma \circ h^{-1}$ where $\sigma :\Sigma \rightarrow \Sigma$ is the shift, that is

$$\begin{aligned} (\sigma \omega )_k=\omega _{k+1},\quad k\in {\mathbb {Z}}. \end{aligned}$$

Moreover, $h^{-1}$ can be defined as follows. Fix $z^*\in Y$ and define $\omega ^*=h^{-1}(z^*)$. Associated to *z* one can define the sequence of hitting times

$$\begin{aligned} t_0=0,\qquad t_k=T\left( \mathtt {P}^{k-1}(z)\right) \quad \text {for}\quad k\ge 1,\qquad t_k=T\left( \mathtt {P}^{k}(z)\right) \quad \text {for}\quad k\le -1. \end{aligned}$$

Then, there exists $C^*\in {\mathbb {N}}$ independent of $z^*$ such that

6.6 $$\begin{aligned} \omega ^*_k=\left\lfloor \frac{t_k-t_{k-1}}{\mathtt {T}_h}\right\rfloor -C^* \end{aligned}$$

where $\mathtt {T}_h$ is the period of the periodic orbit $\mathtt {P}^{\pm , 0 }_{h,\varepsilon }$.

This proposition gives symbolic dynamics for a Poincaré map associated to Hamiltonian (5.1). Note that it is constructed in a way that higher symbols in $\Sigma$ imply longer return times. In particular those can be unbounded. The proof of this proposition follows the same lines as the construction of symbolic dynamics done by Moser in Chapter 3 of [37]. Note that the natural $C^*$ in (6.6) is just to normalize and have as symbols ${{\mathbb {N}}}$ (since the horseshoe is build close the homoclinic orbit, the hitting times satisfy $|t_k-t_{k-1}|\gg 1$).

We remark that condition (5.9) is necessary, indeed the term that breaks the integrability in the Hamiltonian (5.1) has the form $d_{12} \,K_1\,K_2$ (see for instance (5.16), (5.17)). Hence if the condition (5.9) does not hold then the Hamiltonian (5.1) is integrable.

### Application to the Wave, Beam and Hartree equations

To proof Theorem 1.3 (and also the result for the Hartree equation (1.10)) by applying Proposition 6.4, one needs to check that the condition (5.9) is satisfied by the resonant models derived from the Hartree, Beam and Wave equations. To thus end, recall the definitions (3.7), (3.8), (3.9) and the symplectic reduction performed in Sect. 4.3. Next lemmas check condition (5.9) under the hypotheses considered for these three equations.

#### Lemma 6.5

Let us consider Hamiltonian (5.1) associated to either the Wave equation (1.1) or the Beam equation (1.2) and to a set $\Lambda$ satisfying Proposition 4.2. Then, the condition (5.9) is satisfied.

#### Lemma 6.6

Let us consider Hamiltonian (5.1) associated to the Hartree equation (1.10) with a potential *V* as in (1.11) and to a set $\Lambda$ satisfying Proposition 4.1 Then for a generic choice of the $\{\gamma _{n}\}_{n\in \Lambda }$, the condition (5.9) is satisfied.

These lemmas, together with Proposition 6.4, complete the proof of Item (i) of Theorem 1.1.

#### Proof of Lemma 6.5

Recall (4.11), (4.23), (4.13), (3.9). For the Wave and Beam equations (1.1), (1.2),

$$\begin{aligned} d_{12}=\frac{3}{32 \mathtt {g}}\,\sum _{\begin{array}{c} 1 \le i \le 4 \\ 5 \le j \le 8 \end{array}} \frac{(-1)^{i+j}}{|n_i|^{\kappa } |n_j|^\kappa }=\frac{3}{32 \mathtt {g}}\left( \sum _{i=1}^4\frac{(-1)^i}{|n_i|^{\kappa }}\right) \left( \sum _{j=5}^8\frac{(-1)^j}{|n_j|^{\kappa }}\right) \end{aligned}$$

where $\kappa =1$ for the Wave equation and $\kappa =2$ for the Beam equation.

We write $d_{12}$ in a different form. To this end, we introduce the following notations. For each finite set of indexes $I=\{i_1,\ldots ,i_n\} \subset \{ 1, \dots , 8 \}$ and for any pair of positive integers $i_1, i_2 \in \{ 1, \dots , 8 \}$, we define

6.7 $$\begin{aligned} \prod ^{I}= \frac{1}{\prod _{k=1}^n |n_{i_k}|^\kappa },\qquad \Delta _{i_1,i_2}= |n_{i_1}|^\kappa - |n_{i_2}|^\kappa . \end{aligned}$$

Using the identities

$$\begin{aligned} \prod ^{i,j} - \prod ^{i,k} = \prod ^{i,j,k} \Delta _{k,j},\qquad \prod ^{i,j,k} - \prod ^{l,j,k} = \prod ^{i,j,k,l} \Delta _{l,i}, \end{aligned}$$

and the fact that the resonance relations (see (4.2), (4.4)) imply $\Delta _{1,2} = \Delta _{4,3}$, $\Delta _{5,6} = \Delta _{8,7}$, one can see that

6.8 $$\begin{aligned} d_{12}= \Delta _{2,1} \Delta _{6,5} \left( |n_3|^\kappa |n_4|^\kappa - |n_1|^\kappa |n_2|^\kappa \right) \left( |n_7|^\kappa |n_8|^\kappa - |n_5|^\kappa |n_6|^\kappa \right) \prod ^{1,2,3,4,5,6,7,8}. \end{aligned}$$

Therefore $d_{12}$ vanishes if one of the following conditions holds

6.9 $$\begin{aligned} |n_1| = |n_2|,\qquad |n_5| = |n_6|,\qquad |n_1||n_2| = |n_3||n_4|,\qquad |n_5||n_6| = |n_7||n_8|. \end{aligned}$$

#### Remark 6.7

We point out that the conditions (6.9) do not involve at the same time modes belonging to two different 4-tuple resonances.

Condition (4.6) implies that the two first conditions cannot be satisfied. We check now that under the hypotheses of Proposition  4.2, one has

6.10 $$\begin{aligned} |n_1||n_2| \ne |n_3||n_4| \end{aligned}$$

(the condition $|n_5||n_6| \ne |n_7||n_8|$ can be checked analogously).

We start with the Beam equation, that is $\kappa =2$. Arguing by contradiction, assume that $n_1,n_2,n_3,n_4$ satisfy $|n_1||n_2| = |n_3||n_4|$, (4.6) and the resonance condition

6.11 $$\begin{aligned} |n_1|^2-|n_2|^2=-|n_3|^2+|n_4|^2 \end{aligned}$$

The resonance relation can be written as

$$\begin{aligned} (|n_1|-|n_2|)(|n_1|+|n_2|)&= (|n_4|-|n_3|)(|n_4|+|n_3|) \end{aligned}$$

Squaring each side, one has

$$\begin{aligned} (|n_1|^2 + |n_2|^2)^2 - 4 |n_1|^2 |n_2|^2= (|n_3|^2 + |n_4|^2)^2 - 4 |n_3|^2 |n_4|^2. \end{aligned}$$

Therefore, since we are assuming $|n_1||n_2| = |n_3||n_4|$, we get $|n_1|^2 + |n_2|^2= |n_3|^2 + |n_4|^2$, which combined with the resonance relation (6.11) leads to $|n_2|^2= |n_3|^2$, which contradicts assumption (4.6).

For the Wave equation (1.1), that is $\kappa =1$, one can proceed analogously, arguing by contradiction. Assume that $n_1,\ldots ,n_4$ satisfy (4.6), the resonance condition

$$\begin{aligned} |n_1|-|n_2|=-|n_3|+|n_4| \end{aligned}$$

and $|n_1||n_2| = |n_3||n_4|$. Squaring the resonance condition and using this last assumption, one has

$$\begin{aligned}|n_1|^2 + |n_2|^2 = |n_3|^2 + |n_4|^2.\end{aligned}$$

Multiplying both sides by $|n_4|^2$ and using again $|n_1||n_2| = |n_3||n_4|$ one obtains $(|n_1|^2 -|n_4|^2)(|n_4|^2 - |n_2|^2) = 0$, which contradicts (4.6). $\quad \square$

#### Proof of Lemma 6.6

Recall (4.23), (4.13). For the Hartree equation (1.10), $d_{12}$ is of the form

$$\begin{aligned} d_{12}=\sum _{k\in I}\alpha _k V_k \end{aligned}$$

where $$\alpha _k\ne 0$$ and

$$\begin{aligned} I:=\{ k\in {\mathbb {Z}}^2 : k=n_i-n_j \,\,\text{ for } \text{ some }\,\,n_i\in {\mathcal {R}}_1, \,\,n_j\in {\mathcal {R}}_2\}. \end{aligned}$$

We observe that the cardinality of *I* is bounded by $4N(4N-1)/2$. Therefore, by condition (4.5), $d_{12}$ is a polynomial in the $4N(4N-1)/2$ variables $\gamma _k$, $k\in I$. Such polynomial is not identically zero because if we set one of the $\gamma _k$’s equal to one and all the others at zero then $d_{12}\ne 0$. $\quad \square$

### End of the proof of Theorem 1.3

Lemmas 6.5, 6.6 imply that condition (5.9) holds and, therefore, Proposition 6.4 can be applied to the resonant models associated to the Wave (1.1), Beam (1.2) and Hartree (1.10) equations. This proposition gives certain orbits of these resonant models. These orbits will be the first order (up to changes of coordinates) of orbits of equations (1.1), (1.2) and (1.10).

Fix $0<\varepsilon \ll 1$ and $0<h\ll 1$ and consider the periodic orbit $\mathtt {P}^{0 }_{h,\varepsilon }$ given by Proposition 6.1, which has period $\mathtt {T}_h$. By Proposition 6.4 there exist a set $Y\subset {\mathcal {S}}_h$ which is an invariant hyperbolic set (a Smale horseshoe) for the Poincaré map associated to the Hamiltonian ${\mathcal {H}}$ in (5.1). This set can be built arbitrarily close to homoclinic points of $\mathtt {P}^{0 }_{h,\varepsilon }$. Fix $\omega \in \Sigma$ such that $|\omega _k|\ge M_0\,\mathtt {T}_{h}$, where $M_0$ satisfies $M_0\gtrsim \log \varepsilon$ and $\mathtt {T}_h$ is the period of the periodic orbit $\mathtt {P}^{0}_{h, \varepsilon }$. Then, Proposition 6.4 ensures that there exists an orbit $\gamma (t)$ of ${\mathcal {H}}$ with initial condition in *Y*,

$$\begin{aligned} \gamma (t):=(\Psi _1(t), \Psi _2(t), K_1(t), K_2(t)), \quad t\in [0, T] \quad \text{ for } \text{ some }\,\,\,T>0, \end{aligned}$$

which satisfies the following. There exists a sequence of times $\{t_k\}_{k\in {\mathbb {Z}}}$ satisfying (6.6) such that $\gamma (t_k)\in {\mathcal {S}}_h$ where ${\mathcal {S}}_h$ is the section defined in (6.5). Note that, by (6.6), the times $t_k$ satisfy

$$\begin{aligned} t_{k+1}=t_{k}+ \mathtt {T}_h (\omega ^*_k+C^*+\theta _k)\qquad \text { for some }\quad \theta _k\in (0,1) \quad \text { and }C^*\in {{\mathbb {N}}}. \end{aligned}$$

By construction, there exists another sequence of times $\{{\bar{t}}_k\}_{k\in {\mathbb {Z}}}$ with ${\bar{t}}_k\in (t_k,t_{k+1})$ such that $\gamma ({\bar{t}}_k)$ satisfies

$$\begin{aligned} K_2({\bar{t}}_k)=\frac{1}{2},\qquad \left| \Psi _2({\bar{t}}_k)-\frac{2\pi }{3}\right| \ll 1. \end{aligned}$$

The Smale horseshoe, can be built arbitrarily close to the invariant manifolds of $\mathtt {P}^{0}_{h, \varepsilon }$ and therefore, one can ensure that there exist intervals

- $I_k\subset (\overline{t_k},t_{k+1})$ such that, for $t\in I_k$, ${\gamma }(t)$ belongs to a $\varepsilon$-neighborhood of $\mathtt {P}^{0}_{h, \varepsilon }$;
- $J_k\subset (t_k, \overline{t_k})$ such that for $t\in J_k$ the orbit $\gamma (t)$ belongs to a ${\mathcal {O}}(\varepsilon )$-neighborhood of $K_2=1$, since the homoclinic orbit obtained in Proposition 6.1 have points ${\mathcal {O}}(\varepsilon )$-close to $K_2=1$.

This behavior implies estimates (1.8) and (1.9) in Theorem 1.3, once we undo the symplectic reductions, the changes of coordinates and we add the error terms as it is explained below.

By Proposition 6.1 the parameterization of the periodic orbit $\mathtt {P}^{0}_{h, \varepsilon }$ is $\varepsilon$-close to (5.8), hence we have that

$$\begin{aligned} K_1(t)=Q(t)+{\tilde{R}}_2(t) \end{aligned}$$

where *Q*(*t*) is the time parameterization of $\mathtt {P}^{0 }_{h,\varepsilon }$ and thus is $\mathtt {T}_h$-periodic, and $\sup _{t\in [0, T]} |{\tilde{R}}_2(t)|\le \varepsilon$.

By the symplectic reduction performed in Sect. 4.3 there exists *r*(*t*) solution of $H_{\mathrm {Res}}$ in (3.16) with Fourier support $\Lambda$ such that

$$\begin{aligned} |r_{n_1}(t)|^2=| K_{1}(t) |^2, \qquad |r_{n_5}(t)|^2=| K_{2}(t) |^2,\qquad \text {for}\qquad t\in [0,T]. \end{aligned}$$

This can be seen using Remark 4.4, which gives also the behavior of the other actions.

Since the solutions of $H_{\mathrm {Res}}$ are invariant under the scaling (3.18), we can consider $r^{\delta }(t):=\delta r(\delta ^2 t)$. Then, $r^{\delta }(t)$ is also a solution of $H_{\mathrm {Res}}$ for $t\in [0, \delta ^{-2} T]$.

Now it only remains to obtain an orbit for the Eqs. 1.1, 1.2 and 1.10 which is close (up to certain changes of coordinates) to $r^{\delta }(t)$. First step is to apply Proposition 3.5. It ensures that there exists $0<\delta _2\ll 1$ such that for all $\delta \in (0, \delta _2)$, there exists a solution *w*(*t*) of $H\circ \Gamma \circ \Psi =H_{\mathrm {Res}}+{\mathcal {R}}'$ such that $w(t)=r^{\delta }(t)+{\widetilde{R}}(t)$ with ${\widetilde{R}}(0)=0$, $\Vert {\widetilde{R}}(t) \Vert _{\rho }\lesssim \delta ^2$ for $t\in [0, \delta ^{-2} T]$. We note that, by Item (ii) of Proposition 3.4, the Birkhoff map $\Gamma$ is $\delta ^3$-close to the identity. Finally the transformations (3.15) and (4.9) preserve the modulus of the Fourier coefficients. The last change of coordinates that one has to apply (for the Wave (1.1) and Beam (1.2) equations) is passing from complex coordinates (3.4) to the original ones. We remark that by (4.6) if $n_i\in \Lambda$ then $-n_i\notin \Lambda$. Thus

$$\begin{aligned} u_{n_i}=\frac{1}{\sqrt{2 |j|}} \Psi _{n_i} \qquad n_i\in \Lambda . \end{aligned}$$

## Transfer of Beating Effects: Proof of Theorem 1.4

We devote this section to prove Theorem 1.4. First, in Sect. 7.1 we prove the transversality of the stable and unstable invariant manifolds of different periodic orbits of the Hamiltonian (4.22). As a consequence of this transversality, we construct orbits which shadow these invariant manifolds for infinite time. Then, in Sect. 7.2 we prove that the resonant models associated to the Wave, Beam and Hartree equations that we consider fit into the framework of Sect. 7.1 and we complete the proof of Theorem 1.4.

### Heteroclinic connections between periodic orbits and their shadowing

Reasoning as in Proposition 6.1, the Hamiltonian ${\mathcal {H}}$ in (4.22) possesses hyperbolic periodic orbits $\mathtt {P}_{\varepsilon ,h,k}^\pm$ at the energy level *h* whose time parameterization is of the form

$$\begin{aligned} \gamma _{\varepsilon ,h,k}^{\pm ,p}(\tau _k) = (\Psi _{\pm ,\varepsilon ,1}^*, \dots ,\Psi _{\pm ,\varepsilon ,k-1}^*, \Psi _k^{(h)}(\tau _k),\Psi _{\pm ,\varepsilon ,k+1}^*, \dots , \Psi _{\pm ,\varepsilon ,N}^*, 0, \dots ,0, K_k^{(h)}(\tau _k),0, \dots ,0) \end{aligned}$$

where

$$\begin{aligned} \Psi _{\pm ,\varepsilon ,1}^*=\pm \Psi _*+{\mathcal {O}}(\varepsilon ) \end{aligned}$$

(see (5.3)) and $( \Psi _k^{(h)}, K_k^{(h)})$ is $\varepsilon$-close to the periodic orbit $\mathtt {P}_h$ (see (5.4)).

When $\varepsilon = 0$, the invariant manifolds $W^u (\mathtt {P}_{0,h,k}^+)$ and $W^s(\mathtt {P}_{0,h,k}^-)$ coincide.

#### Proposition 7.1

Take any $i,j=1,\ldots , N$, $i\ne j$. Assume that the condition (5.41) is satisfied (see (5.40), (4.23)). Then, there exists $\varepsilon _0>0$ such that for $\varepsilon \in (0, \varepsilon _0)$ and $h_0>0$ such that for any $h \in (0, h_0)$, the manifolds $W^u(\mathtt {P}_{\varepsilon , h, i}^-)$ and $W^s(\mathtt {P}_{\varepsilon , h, j}^+)$ intersect transversally within the energy level.

The transversality of the invariant manifolds allows to construct orbits which shadow them. Note that in the coordinates introduced in (4.25), the periodic orbits $\mathtt {P}_{0,h,k}^+$ and $\mathtt {P}_{\varepsilon ,h,k}^-$ blow down to the same periodic orbit, which we denote by $\mathtt {P}_{0,h,k}$. In the coordinates (4.25), Proposition 7.1 can be restated as that the manifolds $W^u(\mathtt {P}_{\varepsilon , h, i})$ and $W^s(\mathtt {P}_{\varepsilon , h, j})$ intersect transversally along an orbit within the energy level.

#### Definition 7.2

We will say that a family of hyperbolic periodic orbits $\{\mathtt {P}_{\ell }\}_{\ell \in {{\mathbb {N}}}}$ of a system of differential equations, is a *transition chain* if $W^u(\mathtt {P}_{\ell }) \pitchfork W^s(\mathtt {P}_{{\ell +1}})$, for all $\ell \in {{\mathbb {N}}}$.

Note that Proposition 7.1 gives full transversality between the invariant manifolds on the energy level. Thus, recalling that ${\mathcal {H}}(\mathtt {P}_{i_\ell })=h$, from now on, we restrict the flow to this energy level, which is a regular manifold.

#### Corollary 7.3

Let $(i_\ell )_{\ell \in {{\mathbb {N}}}}$, with $i_\ell \in \{1,\dots ,N\}$, be any sequence. Then, if $\varepsilon >0$ is small enough, there exists $h_0$ such that for any $0<h<h_0$, $\{\mathtt {P}_{\varepsilon ,h,i_\ell }\}_{\ell \in {{\mathbb {N}}}}$ is a transition chain of Hamiltonian ${\mathcal {H}}$ on the manifold ${\mathcal {H}}=h$.

#### Proposition 7.4

Let $(i_\ell )_{\ell \in {{\mathbb {N}}}}$, with $i_\ell \in \{1,\dots ,N\}$, be any sequence. Assume that $\varepsilon >0$ is small enough such that $h_0$ in Corollary 7.3 exists. Let $\{\mathtt {P}_{\varepsilon ,h,i_\ell }\}_{\ell \in {{\mathbb {N}}}}$ be a transition chain of Hamiltonian ${\mathcal {H}}$. Let $(\nu _\ell )_{\ell \in {{\mathbb {N}}}}$, with $\nu _\ell >0$, be an arbitrary sequence. Let $N_{\ell } := \{z\mid d(z,\mathtt {P}_{\varepsilon ,h,i_\ell })<\nu _{\ell }\}$. Then, there exists a trajectory $\gamma (t)$ of Hamiltonian ${\mathcal {H}}$ in (4.22) and an increasing sequence $(t_\ell )_{\ell \in {{\mathbb {N}}}}$ of times such that $\gamma (t_\ell ) \in N_{\ell }$, for all $\ell \in {{\mathbb {N}}}$.

#### Proof

Since $\{\mathtt {P}_{\varepsilon ,h,i_\ell }\}_{\ell \in {{\mathbb {N}}}}$ is a transition chain then the Inclination Lemma in [15] (Theorem 4.5) ensures that $W^s_\varepsilon ( \mathtt {P}_{\varepsilon ,h,i_{\ell }})\subseteq \overline{ \cup _{t\le 0} \Phi ^t_{{\mathcal {H}}}(W_{\varepsilon }^{s} (\mathtt {P}_{i_{\ell }+1}))}$ for all $\ell \in {\mathbb {N}}$^7^ .

Let $x\in W^s_\varepsilon (\mathtt {P}_{i_0})$. We can find a closed ball $B_0$ centered at *x* such that

7.1 $$\begin{aligned} \Phi _{{\mathcal {H}}}^{t_0}(B_0)\subset N_0 \end{aligned}$$

for some $t_0>0$. By the inclination Lemma we have that $W_{\varepsilon }^s(\mathtt {P}_{i_1})\cap B_0\ne \emptyset$. Hence we can find a closed ball $B_1$ centered at a point in $W_{\varepsilon }^s(\mathtt {P}_{i_1})\cap B_0$ such that, besides satisfying (7.1),

$$\begin{aligned} \Phi _{{\mathcal {H}}}^{t_1}(B_1)\subset N_1 \end{aligned}$$

for some $t_1>t_0$. Proceeding by induction we can construct a sequence of closed nested balls $B_{{i+1}}\subset B_{i} \subset \dots \subset B_0$ and times $t_{i+1}>t_i>\ldots >t_0$ such that

$$\begin{aligned} \Phi _{{\mathcal {H}}}^{t_j} (B_i)\subset N_{j}, \qquad i\ge j. \end{aligned}$$

Since the balls are compact, the intersection $\cap _{n\ge 0} B_n$ is non-empty, and we can consider $\gamma (t)$ as an orbit with initial datum in that set. $\quad \square$

#### Proof of Proposition 7.1

We proceed as in Sect. 5.2.2 by considering an auxiliary parameter $\delta$ and the Hamiltonian ${\mathcal {H}} = \sum _{j=1}^N {\mathbf {H}}_0^{(j)} + \varepsilon {\mathbf {H}}_1$ defined in (5.42). The Hamiltonian (5.42) has two saddle points,

$$\begin{aligned} {\mathfrak {e}}^{(0)}_{\pm ,\varepsilon }= (\Psi _{\pm , \varepsilon ,1}^*,\dots , \Psi _{\pm , \varepsilon ,N}^*, 0,\dots ,0), \end{aligned}$$

which, for $\varepsilon = 0$ are ${\mathfrak {e}}^{(0)}_{\pm }$ (see (5.43)). For $\varepsilon = 0$ and any $\delta >0$ small, they are connected by the homoclinic manifolds

7.2 $$\begin{aligned} \gamma _0(\mathbf {\tau } ) = (\Psi _1^{(0)}(\tau _1), \ldots , \Psi _N^{(0)}(\tau _N), K_1^{(0)}(\tau _1), \ldots , K_N^{(0)}(\tau _N)), \quad \mathbf {\tau }:=(\tau _1, \dots , \tau _N), \end{aligned}$$

where $\Psi _j^{(0)}$, $K_j^{(0)}$, $j=1,\dots , N$, have been introduced in (5.18). This parametrization of the homoclinic manifold satisfies ${\Phi _{{\mathcal {H}}}^t}_{\mid \varepsilon = 0} \gamma _0(\mathbf {\tau } ) = \gamma _0(\tau _1+t, \dots ,\tau _n+t)$. Fix $1\le k \le N$. The set

$$\begin{aligned} \pi _k = \{(\Psi _1,\dots , \Psi _N, K_1,\dots ,K_N): \;K_\ell = 0, \; \ell \ne k\} \end{aligned}$$

is invariant by the flow of ${\mathcal {H}}$ for any $\varepsilon$ and $\delta$ (this is properly seen in the coordinates (4.25), since then $\pi _k$ corresponds to $x_\ell =y_\ell =0$, $\ell \ne k$, see (4.26)).

The dynamics on the $\pi _k$ plane is integrable and is given by the 1-d.o.f. Hamiltonian ${\mathbf {H}}_0^{(k)} + \varepsilon {{\mathbf {H}}_1}_{\mid \pi _k}$. This Hamiltonian has two saddles $(\Psi _{\pm ,\varepsilon ,\ell }^*,0)$ $\varepsilon$-close to $(\pm \Psi _*,0)=(\pm 2\pi /3,0)$, at the zero energy level. For $h>0$ small, the set $\{{\mathbf {H}}_0^{(k)} + \varepsilon {{\mathbf {H}}_1}_{\mid \pi _k}=h\}$ is a periodic orbit, whose period tends to infinity when *h* goes to 0. Let $(\Psi _k^{(h)}(\tau _k), K_k^{(h)}(\tau _k))$ be a time parametrization of this periodic orbit satisfying

7.3 $$\begin{aligned} \lim _{h\rightarrow 0} (\Psi _k^{(h)}(0), K_k^{(h)}(0)) = (\Psi _k^{(0)}(0), K_k^{(0)}(0)), \end{aligned}$$

where $(\Psi _k^{(0)}, K_k^{(0)})$ are components of the homoclinic manifold introduced in (7.2).

Then, the Hamiltonian ${\mathcal {H}}$ possesses two hyperbolic periodic orbits $\mathtt {P}_{\varepsilon ,h,k}^\pm$ at the energy level *h*, whose time parametrization is given by

7.4 $$\begin{aligned}&\gamma _{\varepsilon ,h,k}^{\pm ,p}(\tau _k) = (\Psi _{\pm ,\varepsilon ,1}^*, \dots ,\Psi _{\pm ,\varepsilon ,k-1}^*, \Psi _k^{(h)}(\tau _k),\Psi _{\pm ,\varepsilon ,k+1}^*, \dots , \nonumber \\&\Psi _{\pm ,\varepsilon ,N}^*, 0, \dots ,0, K_k^{(h)}(\tau _k),0, \dots ,0). \end{aligned}$$

When $\varepsilon = 0$, the invariant manifolds $W^u (\mathtt {P}_{0,h,k}^+)$ and $W^s(\mathtt {P}_{0,h,k}^-)$ coincide. This homoclinic manifold can be parameterized as

7.5 $$\begin{aligned} \gamma _{h,k}(\mathbf {\tau } ) = (\Psi _1^{(0)}(\tau _1), \ldots , \Psi _k^{(h)}(\tau _k), \dots , \Psi _N^{(0)}(\tau _N), K_1^{(0)}(\tau _1), \ldots , K_k^{(h)}(\tau _k), \dots , K_N^{(0)}(\tau _N)), \end{aligned}$$

where $(\Psi _k^{(0)}, K_k^{(0)})$ are components of the homoclinic manifold introduced in (7.2).

Now, fix $i,j \in \{1,\dots , N\}$. For small $\varepsilon >0$, the periodic orbits $\mathtt {P}_{\varepsilon ,h,i}^-$, $\mathtt {P}_{\varepsilon ,h,j}^+$ and their invariant manifolds, $W^s(\mathtt {P}_{\varepsilon ,h,i}^+)$ and $W^u(\mathtt {P}_{\varepsilon ,h,j}^-)$ persist slightly deformed. We show now that the perturbation allows them to intersect.

In order to analyze the possible intersection, we introduce a *N*-dimensional section in the following way. We define, taking into account (5.42),

7.6 $$\begin{aligned} \widetilde{{\mathbf {H}}}_0^{(k)} = {\mathbf {H}}_0^{(k)} + \varepsilon \widehat{{\mathbf {H}}}_0^{(k)}, \qquad k = 1,\dots ,N, \end{aligned}$$

where

$$\begin{aligned} \widehat{{\mathbf {H}}}_0^{(k)}(\psi _k,K_k) = a_{k} K_k + (b_{k}-1) K^2_k +c_k\,K_k (1-K_k)\,\cos (\psi _k) \end{aligned}$$

only depends on $(\psi _k,K_k)$. We have that $\widetilde{{\mathbf {H}}}_0^{(k)}$ is integrable and ${\mathcal {H}}$ can be also written as

7.7 $$\begin{aligned} {\mathcal {H}} = \sum _{k=1}^N {\mathbf {H}}_0^{(k)} + \varepsilon {\mathbf {H}}_1 = \sum _{k=1}^N \widetilde{{\mathbf {H}}}_0^{(k)} + \varepsilon \widetilde{{\mathbf {H}}}_1 \end{aligned}$$

where

7.8 $$\begin{aligned} \widetilde{{\mathbf {H}}}_1(K_1,\dots ,K_n) = \sum _{k, \ell =1, k<\ell }^N d_{k \ell } K_k\,K_\ell . \end{aligned}$$

We consider the *N*-dimensional section

7.9 $$\begin{aligned} \Sigma (\mathbf {\tau })=\left\{ \gamma _{0}(\mathbf {\tau })+\sum _{k=1}^ Nr_k\,{\nabla \widetilde{{\mathbf {H}}}^{(k)}_0}_{\mid \gamma _0(\mathbf {\tau })}, r=(r_1,\dots ,r_{N})\in (-m, m)^{N}\right\} \end{aligned}$$

where $\gamma _0$ is the homoclinic manifold introduced in (7.2). Observe that $\gamma _{0}(\mathbf {\tau })$, which is *N*-dimensional, intersects transversally $\Sigma (\mathbf {\tau })$ at $r=0$. Then, for *h* small, $\gamma _{h,i}$ and $\gamma _{h,j}$ (see (7.5)) intersect transversally $\Sigma (0)$ at points $r_{i}$ and $r_{j}$, close to $\gamma _{0}(\mathbf {\tau })$. Hence, for $\varepsilon$ small enough, the invariant manifolds $W^u(\mathtt {P}_{\varepsilon ,h,i}^-)$ and $W^s(\mathtt {P}_{\varepsilon ,h,j}^+)$ intersect transversally $\Sigma (0)$ at points $r_{\varepsilon ,i}$ and $r_{\varepsilon ,j}$ close to $r_{i}$ and $r_{j}$, respectively.

Let $\gamma _{\varepsilon ,h,i}^u$ and $\gamma _{\varepsilon ,h,j}^s$ be parametrizations of the perturbed invariant manifolds $W^u(\mathtt {P}_{\varepsilon ,h,i}^-)$ and $W^s(\mathtt {P}_{\varepsilon ,h,j}^+)$ such that $\gamma _{\varepsilon ,h,i}^u(0) = r_{\varepsilon ,i}$, $\gamma _{\varepsilon ,h,j}^s(0) = r_{\varepsilon ,j}$ and $\Phi ^t_{{\mathcal {H}}} \gamma (\tau _1,\dots , \tau _n) = \gamma (\tau _1+t,\dots , \tau _N+t)$, for $\gamma = \gamma _{\varepsilon ,h,i}^{u},\gamma _{\varepsilon ,h,j}^{s}$, where $\Phi ^t_{{\mathcal {H}}}$ is the flow of Hamiltonian ${\mathcal {H}}$. Up to a shift in the initial conditions in the periodic orbits, the parameterization of the periodic orbits and the homoclinic manifold satisfy the following property: for any $\tau$ there exists constants $\lambda , K, M>0$ such that

$$\begin{aligned} \begin{aligned} \Vert \gamma _{\varepsilon ,h,k}^{u}(\tau _1+t,\ldots ,\tau _N+t) - \gamma _{\varepsilon ,h,i}^{-,p}(\tau _i+t)\Vert \le&K e^{\lambda t}\qquad&\text { for }\quad t\le M\\ \Vert \gamma _{\varepsilon ,h,k}^{s}(\tau _1+t,\ldots ,\tau _N+t) - \gamma _{\varepsilon ,h,j}^{+,p}(\tau _j+t)\Vert \le&K e^{- \lambda t}\qquad&\text { for }\quad t\ge M. \end{aligned} \end{aligned}$$

Let us remark that ${\mathcal {H}}_{\mid W^u (\mathtt {P}_{\varepsilon ,h,i}^-)}={\mathcal {H}}_{\mid W^s (\mathtt {P}_{\varepsilon ,h,j}^+)}=h$. Therefore, to analyze their intersections it is enough to measure their distance along $(N-1)$-directions of those defining the section $\Sigma$ in (7.9). That is, the manifolds $W^u (\mathtt {P}_{\varepsilon ,h,i}^-)$ and $W^s (\mathtt {P}_{\varepsilon ,h,j}^+)$ intersect transversally along an orbit at the non-degenerate zeros of the vector function (see (7.6))

7.10 $$\begin{aligned} d_{\varepsilon ,h}(\mathbf {\tau }) = \begin{pmatrix} \widetilde{{\mathbf {H}}}_0^{(1)}(\gamma _{\varepsilon ,h,i}^u(\mathbf {\tau }))- \widetilde{{\mathbf {H}}}_0^{(1)}(\gamma _{\varepsilon ,h,j}^s(\mathbf {\tau })) \\ \vdots \\ \widetilde{{\mathbf {H}}}_0^{(N-1)}(\gamma _{\varepsilon ,h,i}^u(\mathbf {\tau }))- \widetilde{{\mathbf {H}}}_0^{(N-1)}(\gamma _{\varepsilon ,h,j}^s(\mathbf {\tau })) \end{pmatrix}. \end{aligned}$$

##### Lemma 7.5

The function $d_{\varepsilon ,h}$ in (7.10) can be written as

7.11 $$\begin{aligned} d_{\varepsilon ,h}(\mathbf {\tau }) = d_{0,h}+ \varepsilon {\mathcal {M}}_h(\mathbf {\tau }) + {\mathcal {O}}\left( \varepsilon ^2\right) , \end{aligned}$$

where the vector $d_{0,h}=(d_{0,h}^1, \ldots d_{0,h}^{N-1})^\top$ is of the form

$$\begin{aligned} d_{0,h}^i=h,\quad d_{0,h}^j=-h\quad \text { and }\quad d_{0,h}^k=0\quad \text { for }\quad k\ne i,j \end{aligned}$$

and $${\mathcal {M}}_h(\mathbf {\tau }) = ( {\mathcal {M}}_{h}^1(\mathbf {\tau }) ,\dots , {\mathcal {M}}_{h}^{N-1}(\mathbf {\tau }))^\top $$, with

$$\begin{aligned} {\mathcal {M}}_{h}^k(\mathbf {\tau }) :=\int _{-\infty }^{0} \{ {\mathbf {H}}_0^{(k)}, \widetilde{{\mathbf {H}}}_1\} \, \circ \, \Phi ^t_{{\mathbf {H}}_0}(\gamma _{h,i}(\mathbf {\tau }))\,dt+\int _0^{\infty } \{ {\mathbf {H}}_0^{(k)}, \widetilde{{\mathbf {H}}}_1\}\, \circ \,\Phi ^t_{{\mathbf {H}}_0}(\gamma _{h,j}(\mathbf {\tau }))\,dt. \end{aligned}$$

##### Proof

We will compute the formula for $\widetilde{{\mathbf {H}}}_0^{(k)}(\gamma _{\varepsilon ,h,i}^u(\mathbf {\tau }))$, $k=1,\dots , N-1$, being the derivation for the one of $\widetilde{{\mathbf {H}}}_0^{(k)}(\gamma _{\varepsilon ,h,j}^s(\mathbf {\tau }))$ analogous.

We first observe that, since ${\mathcal {H}}_{\mid \mathtt {P}_{\varepsilon ,h,i}^-}= h$ and $\widetilde{{\mathbf {H}}}_{1\,\mid \mathtt {P}_{\varepsilon ,h,i}^-}= 0$, $\widetilde{{\mathbf {H}}}_0^{(k)}(\gamma _{\varepsilon ,h,i}^{-,p}(\mathbf {\tau })) = \delta _{ik}h$, being $\delta _{ik}$ the Kronecker’s delta. Then, taking into account (7.7), it is immediate that

$$\begin{aligned} \begin{aligned} \widetilde{{\mathbf {H}}}_0^{(k)}(\gamma _{\varepsilon ,h,i}^u(\mathbf {\tau }))&= \widetilde{{\mathbf {H}}}_0^{(k)}(\gamma _{\varepsilon ,h,i}^u(\mathbf {\tau })) - \widetilde{{\mathbf {H}}}_0^{(k)}(\gamma _{\varepsilon ,h,i}^{-,p}(\mathbf {\tau })) + \delta _{ik} h \\&= \int _{-\infty }^0 \frac{d}{dt} \widetilde{{\mathbf {H}}}_0^{(k)} \circ \Phi _{{\mathcal {H}}}^t \gamma _{\varepsilon ,h,i}^u(\mathbf {\tau })\, dt + \delta _{ik} h \\&= \varepsilon \int _{-\infty }^0 \{ \widetilde{{\mathbf {H}}}_0^{(k)}, \widetilde{{\mathbf {H}}}_1\} \circ \Phi _{{\mathcal {H}}}^t( \gamma _{\varepsilon ,h,i}^u(\mathbf {\tau }))\, dt + \delta _{ik} h \\&= \varepsilon \int _{-\infty }^0 \{ \widetilde{{\mathbf {H}}}_0^{(k)}, \widetilde{{\mathbf {H}}}_1\} \circ \Phi _{{\mathbf {H}}_0}^t ( \gamma _{h,i}(\mathbf {\tau }))\, dt + \delta _{ik} h + {\mathcal {O}}(\varepsilon ^2) \\&= \varepsilon \int _{-\infty }^0 \{ {\mathbf {H}}_0^{(k)}, \widetilde{{\mathbf {H}}}_1\} \circ \Phi _{{\mathbf {H}}_0}^t (\gamma _{h,i}(\mathbf {\tau }))\, dt + \delta _{ik} h + {\mathcal {O}}(\varepsilon ^2), \end{aligned} \end{aligned}$$

where $\gamma _{h, i}$ is defined in (7.5). $\quad \square$

We observe that, since the components of $d_{0,h}$ are either 0 or $\pm h$, if we consider $h\ll \varepsilon$, the main order of the difference in (7.11) is given by ${\mathcal {M}}_h(\mathbf {\tau })$. Thus we shall prove that this function has a non-degenerate zero, so that we can conclude by the Implicit Function Theorem that the manifolds $W^s_\varepsilon (\mathtt {P}^-_{\varepsilon , h, i})$ and $W^u_\varepsilon (\mathtt {P}^+_{\varepsilon , h, j})$ intersect transversally.

To do so, we introduce

7.12 $$\begin{aligned} {\mathcal {M}}_{0}(\mathbf {\tau }) := ( {\mathcal {M}}_0^1(\mathbf {\tau }),\dots , {\mathcal {M}}_0^{N-1}(\mathbf {\tau }))^\top , \end{aligned}$$

where

$$\begin{aligned} {\mathcal {M}}_{0}^k(\mathbf {\tau }) := \int _{-\infty }^{\infty } \{ {\mathbf {H}}_0^{(k)}, \widetilde{{\mathbf {H}}}_1\}\,\circ \,\Phi ^t_{{\mathbf {H}}_0}(\gamma _{0}(\mathbf {\tau }))\,dt, \end{aligned}$$

where $\gamma _{0}$ was introduced in (7.2) (see also (5.18)), which is the Melnikov function associated to the homoclinic between ${\mathfrak {e}}^{(0)}_{\pm }$. We observe that the derivative of the Melnikov potential (5.44) with respect to the variable $\tau _k-\tau _N$ coincides with the Melnikov integral ${\mathcal {M}}_0^k(\mathbf {\tau })$ in (7.12): Indeed, recall that the Melnikov Potential integral associated to $({\mathbf {H}}_1-\widetilde{{\mathbf {H}}}_1)$ is constant, and equivalently

$$\begin{aligned} {\mathcal {M}}_{0}^k(\mathbf {\tau }) = \int _{-\infty }^{\infty } \{ {\mathbf {H}}_0^{(k)}, \widetilde{{\mathbf {H}}}_1\}\,\circ \,\Phi ^t_{{\mathbf {H}}_0}(\gamma _{0}(\mathbf {\tau }))\,dt= \int _{-\infty }^{\infty } \{ {\mathbf {H}}_0^{(k)}, {\mathbf {H}}_1\}\,\circ \,\Phi ^t_{{\mathbf {H}}_0}(\gamma _{0}(\mathbf {\tau }))\,dt. \end{aligned}$$

Then, by Proposition 5.12, if condition (5.41) is satisfied and $\varepsilon >0$ is small enough, ${\mathcal {M}}_{0}(\mathbf {\tau })$ has a non-degenerate zero.

##### Lemma 7.6

Let $\varepsilon >0$ and assume that the condition (5.41) in Proposition 5.12 is satisfied. Then, there exists $h_0$ such that for any $0<h<h_0$, ${\mathcal {M}}_{h}(\mathbf {\tau })$ has a non-degenerate zero.

This lemma implies Proposition 7.1; indeed, one can proceed as in Sect. 5.2.2 by taking $\delta =\varepsilon$ and applying Implicit Function Theorem. We devote the rest of the Section to prove Lemma 7.6.

##### Proof of Lemma 7.6

We have that, for any $1\le k \le N-1$,

$$\begin{aligned}&\int _{-\infty }^0 \{ {\mathbf {H}}_0^{(k)}, \widetilde{{\mathbf {H}}}_1\}\,\circ \,\Phi ^t_{{\mathbf {H}}_0}(\gamma _{h,i}(\mathbf {\tau }))\,dt +\int _0^{\infty } \{ {\mathbf {H}}_0^{(k)}, \widetilde{{\mathbf {H}}}_1\}\,\circ \,\Phi ^t_{{\mathbf {H}}_0}(\gamma _{h,j}(\mathbf {\tau }))\,dt-{\mathcal {M}}_0^{k}(\mathbf {\tau })\\&\quad =\int _{-\infty }^0 \Big (\{ {\mathbf {H}}_0^{(k)}, \widetilde{{\mathbf {H}}}_1\}\,\circ \,\Phi ^t_{{\mathbf {H}}_0}(\gamma _{h,i}(\mathbf {\tau }))-\{ {{\mathbf {H}}}_0^{(k)}, \widetilde{{\mathbf {H}}}_1\}\,\circ \,\Phi ^t_{{\mathbf {H}}_0}(\gamma _{0}(\mathbf {\tau }))\,\Big )\,dt\\&\quad \quad +\int _0^{\infty }\Big (\{ {\mathbf {H}}_0^{(k)}, \widetilde{{\mathbf {H}}}_1\}\,\circ \,\Phi ^t_{{\mathbf {H}}_0}(\gamma _{h,j}(\mathbf {\tau }))-\{ {\mathbf {H}}_0^{(k)}, \widetilde{{\mathbf {H}}}_1\}\,\circ \,\Phi ^t_{{\mathbf {H}}_0}(\gamma _{0}(\mathbf {\tau }))\,\Big )\,dt \\&\quad = I_{h,i}(\mathbf {\tau })+I_{h,j}(\mathbf {\tau }). \end{aligned}$$

We prove that, for any compact set ${\mathbf {K}}\subset {\mathbb {R}}^{N}$, $\Vert I_{h,k}\Vert _{C^1({\mathbf {K}})}$ tends to 0 as $h\rightarrow 0$, for $k=i,j$. We give the argument for $I_{h,i}$, being the one for $I_{h,j}$ analogous. The claim follows immediately from this convergence.

Let ${\mathbf {K}}\subset {\mathbb {R}}^{N}$ be a compact set. If *h* is small enough, the parametrization $\gamma _{h,i}$ is well defined; since the period of the periodic orbit tends to infinity when *h* goes to 0, $\gamma _{h,i}$ intersects $\Sigma (\mathbf {\tau })$ at a point close to $r=0$, for all $\mathbf {\tau } \in {\mathbf {K}}$.

For a given $T>0$, we split the integral $I_{h,i}$ as

7.13 $$\begin{aligned} \begin{aligned} I_{h,i} (\mathbf {\tau }) =&\int _{-T}^0 \Big (\{ {\mathbf {H}}_0^{(k)}, \widetilde{{\mathbf {H}}}_1\}\,\circ \,\Phi ^t_{{\mathbf {H}}_0}(\gamma _{h,i}(\mathbf {\tau })) -\{ {\mathbf {H}}_0^{(k)}, \widetilde{{\mathbf {H}}}_1\}\,\circ \,\Phi ^t_{{\mathbf {H}}_0}(\gamma _{0}(\mathbf {\tau }))\,\Big )\,dt \\&+ \int _{-\infty }^{-T} \Big (\{ {\mathbf {H}}_0^{(k)}, \widetilde{{\mathbf {H}}}_1\}\,\circ \,\Phi ^t_{{\mathbf {H}}_0}(\gamma _{h,i}(\mathbf {\tau }))-\{ {\mathbf {H}}_0^{(k)}, \widetilde{{\mathbf {H}}}_1\}\,\circ \,\Phi ^t_{{\mathbf {H}}_0}(\gamma _{0}(\mathbf {\tau }))\,\Big )\,dt. \end{aligned} \end{aligned}$$

From (5.42), (7.8)

7.14 $$\begin{aligned} \{ {\mathbf {H}}_0^{(k)}, \widetilde{{\mathbf {H}}}_1\} = - 2 K_k(1-K_k) \sin \psi _k \sum _{\ell \ne k} d_{\ell ,k} K_\ell . \end{aligned}$$

In particular,

$$\begin{aligned} \{ {\mathbf {H}}_0^{(k)}, \widetilde{{\mathbf {H}}}_1\}_{\mid {\mathfrak {e}}^{(0)}_{-}} = \{ {\mathbf {H}}_0^{(k)}, \widetilde{{\mathbf {H}}}_1\}_{\mid \mathtt {P}^-_{0, h, i}} = 0. \end{aligned}$$

The hyperbolic character of the periodic orbits implies the existence of constants $C, \lambda >0$ such that, for $\ell \ne i$ and for all $\mathbf {\tau }\in {\mathbf {K}}$,

$$\begin{aligned} |\pi _{K_\ell } \gamma _{h,i}(\mathbf {\tau }) | \le C e^{-\lambda (\tau _\ell + t)}, \qquad t \ge 0. \end{aligned}$$

Also, with the same $C, \lambda >0$, for any $1 \le \ell \le N$,

$$\begin{aligned} |\pi _{K_\ell } \gamma _{0}(\mathbf {\tau }) | \le C e^{-\lambda (\tau _\ell + t)}, \qquad t \ge 0. \end{aligned}$$

Hence, by (7.14), for any $\nu >0$, since ${\mathbf {K}}$ is compact, there exists $T>0$ such that, for any $\mathbf {\tau } \in {\mathbf {K}}$,

$$\begin{aligned} \left| \int _{-\infty }^{-T} \{ {\mathbf {H}}_0^{(k)}, \widetilde{{\mathbf {H}}}_1\}\,\circ \,\Phi ^t_{{\mathbf {H}}_0}(\gamma _{h,i}(\mathbf {\tau }))\,dt \right| , \, \left| \int _{-\infty }^{-T} \{ {\mathbf {H}}_0^{(k)}, \widetilde{{\mathbf {H}}}_1\}\,\circ \,\Phi ^t_{{\mathbf {H}}_0}(\gamma _{0}(\mathbf {\tau }))\,dt \right| \le \nu . \end{aligned}$$

To bound the other part of $I_{h,i}$ in (7.13), we observe that, from (6.1),

$$\begin{aligned}&\gamma _{h,i}(\mathbf {\tau }) - \gamma _{0}(\mathbf {\tau }) = (0, \ldots , \Psi _i^{(h)}(\tau _i)\\&\quad -\Psi _i^{(0)}(\tau _i), \dots , 0, 0, \ldots , K_i^{(h)}(\tau _i)-K_i^{(0)}(\tau _i), \dots , 0). \end{aligned}$$

We remark that, as *h* goes to 0, the period of the periodic orbit $\mathtt {P}_{0,h,i}^-$ goes to $\infty$. Then, the choice of the parametrization of the periodic orbit (7.3) implies that, taking *h* small enough, $\lim _{h\rightarrow 0}(\Psi _i^{(h)}(\tau _i+t), K_i^{(h)}(\tau _i+t)) = (\Psi _i^{(0)}(\tau _i+t), K_i^{(0)}(\tau _i+t))$, for all $(t, \mathbf {\tau }) \in [-T,0]\times {\mathbf {K}}$ and, furthermore, this convergence is the $C^k$-norm on $[-T,0]\times {\mathbf {K}}$. In particular, this implies that, if *h* is small enough,

$$\begin{aligned} \left| \int _{-T}^0 \Big (\{ {\mathbf {H}}_0^{(k)}, \widetilde{{\mathbf {H}}}_1\}\,\circ \,\Phi ^t_{{\mathbf {H}}_0}(\gamma _{h,i}(\mathbf {\tau })) -\{ {\mathbf {H}}_0^{(k)}, \widetilde{{\mathbf {H}}}_1\}\,\circ \,\Phi ^t_{{\mathbf {H}}_0}(\gamma _{0}(\mathbf {\tau }))\,\Big )\,dt \right| < \nu . \end{aligned}$$

$$\square $$

### Application to the Wave, Beam and Hartree equations: Proof of Theorem 1.1-(ii)

Recall the matrix (5.40). We now check the condition (5.41) in Proposition 5.12 for the resonant models associated to the equations (1.1), (1.2) and (1.10). For the Wave and Beam equations this corresponds to choosing suitable sets $\Lambda$ (actually a suitable modification of those obtained in Proposition 4.2). For the Hartree equation this corresponds to imposing a non-degeneracy condition to the potential *V*.

#### Lemma 7.7

Let us consider either the Wave equation (1.1) or the Beam equation (1.2). Then, there exists a set $\Lambda \subset {\mathbb {Z}}^2$ satisfying Propositions (4.2) such that the associated Hamiltonian (5.1) satisfies condition (5.41).

#### Lemma 7.8

Let us consider Hamiltonian (5.1) associated to the Hartree equation (1.10) with a potential *V* as in (1.11) and to a set $\Lambda$ satisfying Proposition 4.1 Then, for a generic choice of the $\{\gamma _{n}\}_{n\in \Lambda }$, the condition (5.41) is satisfied.

These two lemmas allow us to complete the proof of Item (ii) of Theorem  1.1

#### Proof of Item (ii) of Theorem 1.1

Lemmas 7.7 and Lemma 7.8 ensure that the non-degeneracy condition (5.41) of Proposition 7.1. Therefore, any pair of periodic orbits $\mathtt {P}_{\varepsilon ,h,i}$, $\mathtt {P}_{\varepsilon ,h,j}$ have transverse heteroclinic connections. This implies that all infinite sequences of such periodic orbits form a transition chain in the sense of Definition 7.2. Then, to complete the proof of Item (ii) of Theorem 1.1 it is enough to apply Proposition 7.4. $\quad \square$

We devote the rest of this section to prove Lemmas 7.7 and 7.8. Lemma 7.8 is proved following the same argument of the proof of Lemma 6.6. To the prove Lemma 7.7, we consider a set $\Lambda _0\subset {\mathbb {Z}}^2$ satisfying Proposition 4.2 and we modify it slightly. By modification, we refer to construct a set $\Lambda \in {\mathbb {Q}}^2$ arbitrarily close to $\Lambda _0\subset {\mathbb {Z}}^2$ and then to scale it so that the set belongs to ${\mathbb {Z}}^2$.

#### Proof of Lemma 7.7

Let us call $n^{(i)}_k:=n_{4 (i-1)+k}$ for $i=1,\dots , N$, $k=1, \dots , 4$. Recall the expression of the coefficients $d_{i j}$ in (4.23). By using (4.13) and Lemma 4.3 we obtain

7.15 $$\begin{aligned} d_{i j}=\frac{3}{32 \mathtt {g}} \,\sum _{\begin{array}{c} 1 \le r, s \le 4 \end{array}} \frac{(-1)^{r+s}}{|n^{(i)}_r|^{\kappa } |n^{(j)}_s|^\kappa }, \end{aligned}$$

where $\kappa =1, 2$ corresponds respectively to the Wave and Beam equations. We define (recall formulas (6.7))

7.16 $$\begin{aligned} P_r=\Delta _{n^{(r)}_{1}, n^{(r)}_{2}} (|n_1^{(r)}|^{\kappa } |n^{(r)}_2|^{\kappa }-|n^{(r)}_3|^{\kappa } |n^{(r)}_4|^{\kappa }) \prod _r^{1, 2, 3, 4} \quad \text {where}\quad \prod _r^{1, 2, 3, 4}&:= \frac{1}{ \prod _{i=1}^4 |n_i^{(r)}|^{\kappa } }. \end{aligned}$$

We remark that by the resonance relations (4.3) and (4.4) we have $\Delta _{n^{(r)}_{1}, n^{(r)}_{2}}=\Delta _{n^{(r)}_{4}, n^{(r)}_{3}}$. Then we can express the right hand side of (7.15) in terms of the $P_r$’s in the following way:

$$\begin{aligned} d_{i j}=\frac{3}{32 \mathtt {g}}\, P_{i} P_j. \end{aligned}$$

Then, the determinant of the matrix ${\mathcal {D}}$ in (5.40) is of the form

$$\begin{aligned} \det ({\mathcal {D}}) = \left( \frac{3}{32\mathtt {g} }\right) ^{N-1}\left( \prod _{k=1}^{N-1} P_k \right) \; \text {det} \; \begin{pmatrix} P_N + \sum _{j \ne 1} P_j &{} \quad -P_2 &{} \quad \ldots &{}\quad -P_{N-1} \\ -P_1 &{}\quad \ddots &{}\quad \vdots &{} \quad \vdots \\ \vdots &{} \quad \vdots &{}\quad \ddots &{} \quad -P_{N-1} \\ -P_1 &{}\quad \ldots &{}\quad -P_{N-2} &{}\quad P_{N} + \sum _{j \ne N-1} P_j \end{pmatrix}. \end{aligned}$$

This determinant can be written as

7.17 $$\begin{aligned} \det ({\mathcal {D}}) = \left( \frac{3}{32\mathtt {g} }\right) ^{N-1}\left( \prod _{k=1}^{N} P_k \right) \left( \sum _{k=1}^{N}P_k\right) ^{N-2}. \end{aligned}$$

Indeed, it is enough to modify the matrix in two steps. First replace the last column by the sum of all columns. Then, the last column is the vector with all components equal to $P_N$. Second, subtract the last row to the other rows. Then, it is very easy to obtain (7.17).

Recall that in the proof of Lemma 6.5 we have shown that the sets $\Lambda$ of Proposition 4.2 satisfy

$$\begin{aligned} |n_1^{(r)}|^{\kappa } |n^{(r)}_2|^{\kappa }-|n^{(r)}_3|^{\kappa } |n^{(r)}_4|^{\kappa }\ne 0 \end{aligned}$$

(see (6.10)). Moreover, in Proposition 4.2 it shown that they also satisfy (4.6). These three properties imply

7.18 $$\begin{aligned} P_k\ne 0\quad \text { for all}\quad k=1,\ldots , N \end{aligned}$$

Therefore, by (7.17), to prove $\det ({\mathcal {D}}) \ne 0$, it only remains to check that

7.19 $$\begin{aligned} \sum _{k=1}^{N}P_k\ne 0. \end{aligned}$$

If the set $\Lambda$ obtained in Proposition 4.2 satisfies this property, the proof is complete. Now, we show that if the set $\Lambda$ obtained in these propositions satisfies $\sum _{k=1}^{N}P_k= 0$, one can modify it slightly so that the new one satisfies (7.19). Assume thus that $\Lambda$ satisfies $\sum _{k=1}^{N}P_k= 0$ and (7.18). Then, we modify the first resonant tuple $(n_1^{(1)},n_2^{(1)},n_3^{(1)},n_4^{(1)})$ to obtain a set $\Lambda \subset {\mathbb {Q}}^2$ which satisfies (7.19). We consider the family of resonant tuples in ${\mathbb {Q}}^2$, given by

$$\begin{aligned} (\lambda n_1^{(1)}, \lambda n_2^{(1)}, \lambda n_3^{(1)}, \lambda n_4^{(1)}), \qquad \lambda \in {\mathbb {Q}}{\setminus }\{0\}. \end{aligned}$$

Then, by (7.16),

$$\begin{aligned} P_1(\lambda n_1^{(1)}, \lambda n_2^{(1)}, \lambda n_3^{(1)}, \lambda n_4^{(1)})=\lambda ^{-\kappa } P_1(n_1^{(1)},n_2^{(1)},n_3^{(1)},n_4^{(1)}). \end{aligned}$$

Then, since $P_1\ne 0$, $P_1$ is strictly decreasing in $\lambda$ and therefore $\sum _{k=1}^{N}P_k= 0$ can only happen for $\lambda =1$. Thus, one can modify the first rectangle by taking $\lambda \in {\mathbb {Q}}$ arbitrarily close to 1 and then blowing up the *N* rectangles so that the new rectangles belong to ${\mathbb {Z}}^2$. It is clear that with this modification (for $\lambda$ close enough to 1) the properties in Proposition 4.2 are still satisfied.

### End of the proof of Theorem 1.4

Lemmas 7.7, 7.8 imply that the assumptions required by Proposition 7.1 hold. Then we can use Proposition 7.4 to deduce dynamical results on the resonant models of equations (1.1), (1.2) and (1.10).

Let us fix $N\ge 2$, $k\gg 1$ and a sequence $\omega _1, \dots , \omega _k$ with $\omega _p\in \{1, \dots , N\}$ for $k=1\ldots k$. We apply Proposition 7.4 choosing $\nu _{\ell }=\varepsilon$ for all $\ell =1, \dots , k$. Then there exist $T>0$ and an orbit

$$\begin{aligned} \gamma (t)=(\Psi _1(t), \dots , \Psi _N(t), K_1(t), \dots , K_N (t)), \quad t\in [0, T] \end{aligned}$$

of the Hamiltonian ${\mathcal {H}}$ (see (4.22)) which has the following behavior: There exists some $\alpha _p,\beta _p$ satisfying $\alpha _p<\beta _p<\alpha _{p+1}$ such that, if one splits the time interval as $[0, T]={{\mathcal {I}}}_1\cup {{\mathcal {J}}}_{1, 2}\cup {{\mathcal {I}}}_2\cup {{\mathcal {J}}}_{2, 3}\cup \dots \cup {{\mathcal {J}}}_{k-1, k }\cup {{\mathcal {I}}}_k$ with

$$\begin{aligned} {{\mathcal {I}}}_p=[\alpha _p,\beta _p],\quad {{\mathcal {J}}}_{p,p+1}=[\delta ^{-2}\beta _p,\delta ^{-2}\alpha _{p+1}], \end{aligned}$$

the orbit $\gamma (t)$ has two different regimes

- *Beating regime:* For $t\in [\alpha _{p}, \beta _{p}]$, $\gamma (t)$ belongs to an $\varepsilon$-neighborhood of the periodic orbit $\mathtt {P}_{\varepsilon , h, \omega _{p}}$. The orbit ${\gamma }(t)$ spends ${\mathcal {O}}(\log \varepsilon )$-time inside this neighborhood and then it leaves it.
- *Transition regime:* For $t\in (\beta _{p}, \alpha _{p+1})$, the orbit $\gamma (t)$ shadows the heteroclinic connection between two hyperbolic periodic orbits $\mathtt {P}_{\varepsilon , h, \omega _{p}}$ and $\mathtt {P}_{\varepsilon , h, \omega _{p+1}}$.

By (7.4), the time parameterization of the periodic orbit $\mathtt {P}_{\varepsilon , h, \omega _{p}}$ satisfies

$$\begin{aligned} K_p(t)=Q(t), \qquad K_i(t)=0\quad \text {for}\quad i\ne p, \end{aligned}$$

where *Q*(*t*) is a periodic orbit. Then, the orbit $\gamma (t)$ satisfies that for $t\in [\alpha _{p}, \beta _{p}]$

$$\begin{aligned} |K_p(t)-Q(t-t_p)|\le \varepsilon ,\qquad | K_i(t) |^2\le \varepsilon \qquad \forall \quad i\ne p, \end{aligned}$$

for some $$t_p>0$$.

In the time interval $(\beta _{\ell }, \alpha _{\ell +1})$, the travel along the heteroclinic connection implies that all the actions $|K_i|^2$ experience a change of order ${\mathcal {O}}(1)$ (see the proof of Proposition 7.1).

By the symplectic reduction performed in Sect. 4.3 there exists *r*(*t*) solution of $H_{\mathrm {Res}}$ in (3.16) with Fourier support $\Lambda$ such that the actions $|r_{n_1^{(\omega _i)}}|^2$ satisfy

$$\begin{aligned} |r_{n_1^{(\omega _i)}}(t)|^2=| K_{i}(t) |^2\qquad \text {for}\qquad t\in [0,T]. \end{aligned}$$

This can be seen using Remark 4.4, which gives also the behavior of the other actions.

Since the solutions of $H_{\mathrm {Res}}$ are invariant under the scaling (3.18), we can consider $r^{\delta }(t):=\delta r(\delta ^2 t)$. Then, $r^{\delta }(t)$ is also a solution of $H_{\mathrm {Res}}$ for $t\in [0, \delta ^{-2} T]$.

Now it only remains to obtain an orbit for the Eqs. 1.1, 1.2 and 1.10 which is close (up to certain changes of coordinates) to $r^{\delta }(t)$. First step is to apply Proposition 3.5. It ensures that there exists $0<\delta _2\ll 1$ such that for all $\delta \in (0, \delta _2)$, there exists a solution *w*(*t*) of $H\circ \Gamma \circ \Psi =H_{\mathrm {Res}}+{\mathcal {R}}'$ such that $w(t)=r^{\delta }(t)+{\widetilde{R}}(t)$ with ${\widetilde{R}}(0)=0$, $\Vert {\widetilde{R}}(t) \Vert _{\rho }\lesssim \delta ^2$ for $t\in [0, \delta ^{-2} T]$. We note that, by Item (ii) of Proposition 3.4, the Birkhoff map $\Gamma$ is $\delta ^3$-close to the identity. Finally the transformations (3.15) and (4.9) preserve the modulus of the Fourier coefficients. The last change of coordinates that one has to apply (for the Wave (1.1) and Beam (1.2) equations) is passing from complex coordinates (3.4) to the original ones. We remark that by (4.6) if $n_i\in \Lambda$ then $-n_i\notin \Lambda$. Thus

$$\begin{aligned} u_{n_i}=\frac{1}{\sqrt{2 |j|}} \Psi _{n_i} \qquad n_i\in \Lambda . \end{aligned}$$

$$\square $$

## A the Set $\Lambda$: Proof of Propositions 4.1 and 4.2

The proofs of Propositions 4.1 and 4.2 are modifications of the proof of the construction of the set $\Lambda \subset {\mathbb {Z}}^2$ in [8]. Note that the resonances of the cubic nonlinear Schrödinger equation considered in [8] are the tuples contained in ${{\widetilde{{\varvec{A}}}}}_{bh}$ in (4.3). We summarize the ideas in that paper and explain the main modifications.

      In [8], the set $\Lambda$ is first constructed in ${\mathbb {Q}}^2$ and then scaled to ${\mathbb {Z}}^2$. The placement of the modes in ${\mathbb {Q}}^2$ is done inductively: first one places the modes in $\Lambda _1$, then those in $\Lambda _2$, checking at each placement that conditions $1_\Lambda$–$4_\Lambda$ are fulfilled. To this end, one has to ensure that the imposed non-degeneracy conditions are open and dense in ${\mathbb {Q}}^2$ and then for “most of the placements” are satisfied. More concretely, the placement goes as follows

- *First generation*: In order to place the first generation we have to chose 2*N* points in ${\mathbb {Q}}^2$. We choose them inductively checking that they satisfy the non-degeneracy conditions. Condition $2_\Lambda$ and $3_\Lambda$ are satisfied if all the points are chosen different and $1_\Lambda$ will be satisfied by construction. The condition $4_\Lambda$ is equivalent to check that each new point does not make a right angle with two of the modes already placed. That is, consider any segment whose endpoints are two points already chosen. Then, this new point cannot belong either to a line orthogonal to this segment and containing one of the points nor to the circle having this segment as a diameter.
- *Second generation*: The set $\Lambda _1$ is divided into pairs of modes, which are the parents of the *N* nuclear families. For each of these pairs $n_1,n_3\in \Lambda _1\subset {\mathbb {Q}}^2$, we place a pair of points $n_2,n_4\in \Lambda _2$ in such a way that they form a rectangle with the other pair. That is, we consider the circle having as a diameter the segment between $n_1$ and $n_3$. Then, the new modes $n_2$,$n_4$ have to be endpoints of another diameter of this circle. To ensure that $n_2,n_4\in {\mathbb {Q}}^2$ it is enough to chose an angle between the two diameters which has rational tangent. Note that those angles are dense. The choice is done checking that the non-degeneracy conditions are verified $1_\Lambda$–$4_\Lambda$ following the same arguments as for the first generation.

This placement is *generic* in the following sense

1. The first generation is placed generically in ${\mathbb {Q}}^2$, that is anywhere except in the zero set of one polynomial.
2. The placement angles $\theta$ for the second generation are any angle such that $\tan \theta \in {\mathbb {Q}}$ except a finite number of values.

We use this scheme developed in [8] to prove Proposition 4.1.

### Proof of Proposition 4.1

It is a direct consequence of the scheme developed in [8]. Indeed, the only extra condition added with respect to [8] is (4.5), which is certainly satisfied by a generic placement in ${\mathbb {Q}}^2$. Indeed, in placing inductively the new points one has only to avoid a finite number of points. $\quad \square$

*Proof of Proposition* 4.2*(Beam case:* ${\varvec{A}}={\varvec{A}}_{bh}$*)* The set $\Lambda$ from Proposition 4.2 has three differences with respect to the one in [8]: properties (4.6) and (4.7) and the fact that the condition $4_\Lambda$ requires that the modes in $\Lambda$ do not satisfy any of the resonance conditions in ${\varvec{A}}_{bh} {\setminus } {{\widetilde{{\varvec{A}}}}}_{bh}$ in (4.2)–(4.3). One can easily check that a generic placement satisfies (4.6) and the $4_\Lambda$ condition. Indeed, in placing the new modes one has to avoid circles centered at zero with radius equal to the norm of the already placed modes and the circles and hyperbolas defined by (4.2) when two modes are fixed.

      To build a set $\Lambda$ having property (4.7), we also follow the ideas in [8]. We first construct a prototype embedding. That is a “bad” set $\Lambda _0\in {\mathbb {Q}}^2$ which is the union of *N* rectangles but which however does not satisfy the non-degeneracy conditions. For instance, consider

$$\begin{aligned} \Lambda _0=\cup _{i=1}^N{\mathcal {R}}_i,\qquad {\mathcal {R}}_i=\{(\pm 1,0),(0,\pm 1)\}. \end{aligned}$$

This embedding satisfies (4.7) but does not satisfy conditions $1_\Lambda - 4_\Lambda$ nor (4.6) (in particular is not injective). However, by genericity one can chose points in ${\mathbb {Q}}^2$ which are $\varepsilon /4$-close to those of $\Lambda _0$ which define a set $\Lambda$ satisfying that all points are different and also conditions $1_\Lambda -4_\Lambda$.

      Finally, one needs to apply a scaling and a translation to obtain a set $\Lambda \subset {\mathbb {Z}}_\mathrm {odd}\times {\mathbb {Z}}$. Indeed, consider $R\gg 1$ such that $R\Lambda \subset {\mathbb {Z}}^2$ and $R\varepsilon \gg 1$. Then, we define

$$\begin{aligned} \Lambda '=2R\Lambda +(1,0) \end{aligned}$$

Then, one can check that for $n'\in \Lambda '$, which is of the form $n'=2Rn+(1,0)$ with $n\in \Lambda$, taking *R* large enough,

$$\begin{aligned} \left| |n'|-2R\right| =2R\left| \left| n+\left( \frac{1}{2R}, 0\right) \right| -1\right| \le \frac{R\varepsilon }{2}+{\mathcal {O}}(1)\le R\varepsilon . \end{aligned}$$

$$\square $$

*Proof of Proposition* 4.2*(Wave case:* ${\varvec{A}}={\varvec{A}}_w$*)* To prove Proposition 4.2 one has to take into account that the resonance condition for the Wave equation (1.1) given in (4.4) is different from that of the cubic nonlinear Schrödinger, Hartree (1.10) and Beam (1.2) equations (see (4.2)). Now four resonant modes $(n_1,n_2,n_3,n_4)\in {\mathbf {A}}_w$ form a parallelogram whose vertices are on an ellipse with one focus at zero. Indeed, if one fixes the modes $n_1$ and $n_3$, then $n_2,n_4$ must belong to the ellipse defined by

A.1 $$\begin{aligned} \left\{ n\in {\mathbb {Q}}^2:|n| + |n-(n_1+n_3)| = |n_1|+|n_3|\right\} , \end{aligned}$$

that is, the ellipse with foci at 0 and $n_1+n_3$ and such that the sum of distances from any point of the ellipse to the two foci is given by $|n_1|+|n_3|$. Note that the case $n_1=-n_3$ trivially corresponds to the circle with center 0 and radius $|n_1|$.

      We need to consider *N* ellipses of this type with dense rational points to apply the genericity arguments as in the previous cases. The standard ellipse

A.2 $$\begin{aligned} \frac{x^2}{a^2}+\frac{y^2}{b^2}=1 \end{aligned}$$

has dense rational points provided $a,b\in {\mathbb {Q}}$. To obtain ellipses of the form (A.1) from (A.2) one needs to apply a translation (one could also apply a rotation, but there is no need for it). To ensure that the transformed ellipse has dense rational points it is enough to ensure that the foci of the standard ellipse (A.2) are rational. Assuming that $a>b$, the foci are given by $F_\pm =(\pm c,0)=(\pm \sqrt{a^2-b^2},0)$.

      Therefore, to build ellipses ${\mathcal {E}}_j$ with dense rational points , $j=1\ldots N$, it is enough to consider *N* different rational Pythagorean triples $\{(a_j,b_j,c_j)\}_{j=1}^N$, that is $a_j^2=b_j^2+c_j^2$, $a_j,b_j,c_j\in {\mathbb {Q}}$, $a_j>b_j$. Then, one can apply a translation to place one of the foci at 0. Let us denote by $F_j$ the focus of the ellipse ${\mathcal {E}}_j$ which is not at the origin.

      Having fixed these ellipses, one can prove Proposition 4.2 following the scheme of [8] explained above. One first places each pair of the first generation in one of the ellipses. To place one pair ${\mathcal {E}}_j$ it is enough to chose one rational point $n_{j_1}\in {\mathcal {E}}_j$. Then, the other mode is obtained through the equation

$$\begin{aligned} n_{j_1}+n_{j_3}=F_j\qquad \text { (see}\,\mathrm{(}A.1\mathrm{))}. \end{aligned}$$

Since the ellipses have dense rational points, one can place the points such that the conditions $2_\Lambda -4_\Lambda$ and (4.6) are satisfied as follows. Let us assume that we have placed all modes of the first generation for the ellipses ${\mathcal {E}}_j$, $j=1\dots j^*-1$ and we want to place the first generation modes in the ellipse ${\mathcal {E}}_{j^*}$. We show that we only need to avoid a finite number of points.

1. For property (4.6), we need to avoid the intersection points of ${\mathcal {E}}_{j^*}$ with all the circles centered at the origin and radius equal to the norm of the already placed modes.
2. For properties $2_\Lambda$, $3_\Lambda$, we need to avoid the points at the intersection of ${\mathcal {E}}_{j^*}$ with the other ellipses ${\mathcal {E}}_j$, $j=1\ldots j^*-1, j^*+1\ldots N$.
3. For property $4_\Lambda$, one needs to avoid placing a mode such that with two previous modes $m,m'$ and an extra mode may create a nuclear family. To this end we have to avoid the following points:
  - Case (i)—$m, m'$ are non adjacent vertices of the parallelogram: One has to avoid the intersection points between ${\mathcal {E}}_{j^*}$ and the ellipse defined by $m,m'$, that is
    $$\begin{aligned} |n|+|n-(m+m')|=|m|+|m'| \end{aligned}$$
    Note that this ellipse is different from ${\mathcal {E}}_{j^*}$ since by Item 2 above, $m,m'\not \in {\mathcal {E}}_{j^*}$.
  - Case (ii)—$m, m'$ are adjacent vertices of the parallelogram: One has to avoid the intersection points between ${\mathcal {E}}_{j^*}$ and the hyperbolas defined by $m,m'$, that is
    $$\begin{aligned} |n|-|n-(m+m')|=\pm |m|\mp |m'|. \end{aligned}$$
  - One can deal analogously with the conditions which arise from avoiding the resonances conditions in ${\varvec{A}}_w$ given by
    $$\begin{aligned} n_1+n_2+n_3-n_4=0,\quad |n_1|+|n_2|+|n_3|-|n_4|=0, \end{aligned}$$
    which either define ellipses or hyperbolas.
  Note that the two new placed modes and one already placed mode cannot be part of a nuclear family since the already placed mode does not belong to the ellipse defined by the two new modes.

One can proceed analogously to place the second generation. Note that this construction implies Property $1_\Lambda$.

To build a set $\Lambda$ satisfying also condition (4.7) it is enough to chose the rational Pythagorean triples $\{(a_j,b_j,c_j)\}_{j=1}^N$ such that $|a_j-1|,|b_j-1|,c_j\ll \varepsilon$ in such a way that the ellipses are $\varepsilon$-close to the unit circle. Note that this is possible since, in particular, rational Pythagorean triples are dense in the unit circle.

This construction gives a set $\Lambda$ in ${\mathbb {Z}}^2$. Note that one cannot scale and translate to construct a set $\Lambda$ in ${\mathbb {Z}}^2_\mathrm {odd}$ as in the proof of Proposition 4.2 for the Beam case. Indeed, the resonance condition (4.3) is not invariant by translation. Instead, we refine the construction of the set $\Lambda$ in ${\mathbb {Q}}^2$ by choosing more carefully the modes.

To this end, we recall that the rational modes on the unit circle are given by

$$\begin{aligned} z=\left( \frac{p_1}{q}, \frac{p_2}{q}\right) =\left( \frac{m^2-n^2}{m^2+n^2} ,\frac{2mn}{m^2+n^2}\right) ,\quad m,n\in {\mathbb {Z}}. \end{aligned}$$

If one choses *m* odd and *n* even one obtains a point $z\in {\mathbb {Q}}^2$ whose denominator is odd and their numerators are odd in the first component and even in the second component. Certainly such points are dense in the unit circle. After a blow up by *q* (or any odd multiple of it), one obtains a point in ${\mathbb {Z}}^2_\mathrm {odd}$.

We show that one can construct a set $\Lambda \subset {\mathbb {Q}}^2$ as just done keeping track of the rational numbers to show that all of them can be chosen of the form

A.3 $$\begin{aligned} z=\left( \frac{{\text {odd}}}{{\text {odd}}},\frac{{\text {even}}}{{\text {odd}}}\right) . \end{aligned}$$

Indeed, one can choose the ellipses ${\mathcal {E}}_j$ with rational Pythagorean triples $\{(a_j,b_j,c_j)\}_{j=1}^N$, $a_j,b_j,c_j\in {\mathbb {Q}}$, such that $a_j$, $b_j$ are of the form $\text {odd}/\text {odd}$ and $c_j$ is $\text {even}/\text {odd}$. Then, the rational points on the ellipse ${\mathcal {E}}_j$ are of the form

$$\begin{aligned} z=\left( c_j+a_j\frac{m^2-n^2}{m^2+n^2} ,b_j\frac{2mn}{m^2+n^2}\right) ,\quad m,n\in {\mathbb {Z}}. \end{aligned}$$

Choosing *m* odd and *n* even, one has a point *z* of the form (A.3). Since points of this form are dense in ${\mathcal {E}}_j$ one can proceed the construction such that all points in $\Lambda \subset {\mathbb {Q}}^2$ are of the form (A.3).

Finally, it only remains to multiply by the least common divisor of all points in $\Lambda$ to obtain a set in ${\mathbb {Z}}^2_\mathrm {odd}$ and the same happens by the multiplication by any odd multiple of the least common divisor. $\quad \square$

## Footnotes

1 It is well known that the existence of such changes of coordinates cannot be always guaranteed because of the presence of small divisor problems and / or derivatives in the nonlinear terms. At this stage, one can consider the normal form truncation as a formal “good first order” of the full equation. To show that is truly a good first order in the regions of the phase space that we consider, we adopt the strategy of performing a *weak version* of the Birkhoff normal form which does not remove all the non-resonant terms but a finite number of them.

2 Actually there exist “many sets” with such properties. See Remark 1.2 below.

3 The condition $\sum _{j=1}^k m_j\le M-k$ is just to ensure that the sequence $\{t_j\}_{j=1}^k$ defined below belongs to the interval $[0, \delta ^{-2} M \mathtt {T}]$.

4 The Birkhoff Normal Form of the cubic NLS (1.13) restricted *N* disjoint resonant tuples is significantly different from that of Eqs. (1.2), (1.1) and (1.10). Indeed, for NLS, using the gauge invariance, one can see that the “restricted normal form” is the sum of *N* uncoupled integrable 4 degrees of freedom Hamiltonian systems. On the contrary, for Eqs. (1.2), (1.1) and (1.10) (even if the Hartree equation does also have the gauge invariance), the “restricted normal form” possesses couplings between modes of different disjoint resonant tuples.

5 These periodic orbits are not fully hyperbolic since the system is Hamiltonian: the tangent to the periodic orbit and its conjugate direction are not hyperbolic.

6 Here we use the standard notation $a_j^+=a_j$, $a_{j}^-= \overline{a_{-j}}$.

7 More precisely we apply Theorem 4.5 in [15]to the flow map $f:=\Phi ^{\tau }_{{\mathcal {H}}}$, where $\tau >0$ is chosen to be not a multiple of any frequency of the periodic orbits $\mathtt {P}_{{\varepsilon ,h,i_\ell }}$. Note that the Inclination Lemma stated in [15] is stated for the unstable manifold; in order to deduce the statement for the stable manifold it suffices to replace *f* by $f^{-1}$.
